# Amyloid β Instigates Cardiac Neurotrophic Signaling Impairment, Driving Alzheimer's Associated Heart Disease

**DOI:** 10.1002/advs.202511924

**Published:** 2026-02-10

**Authors:** Andrea Elia, Rebecca Parodi‐Rullan, Rafael Vazquez‐Torres, Ashley Carey, Huaqing Zhao, Sabzali Javadov, Silvia Fossati

**Affiliations:** ^1^ Alzheimer's Center at Temple (ACT) Department of Neural Sciences Lewis Katz School of Medicine Temple University Philadelphia Pennsylvania USA; ^2^ Department of Physiology University of Puerto Rico School of Medicine San Juan USA; ^3^ Center for Biostatistics and Epidemiology Department of Biomedical Education and Data Science Temple University Lewis Katz School of Medicine Philadelphia Pennsylvania USA

**Keywords:** Alzheimer's disease, brain‐heart axis neuro‐signaling pathway, cardiac dysfunction, human iPSC‐derived cardiomyocytes, human post‐mortem heart

## Abstract

While a link between cardiovascular risk factors and increased Alzheimer's disease (AD) risk has been reported, it remains unclear whether AD pathology has a direct effect on cardiac function and myocardial innervation. AD and amyloidosis are known to impair neuronal function and affect brain neurotrophic factors (NGF and BDNF) expression. Amyloid aggregates and neuro‐signaling impairments may also expose AD patients to peripheral nervous system deficits, promoting cardiac disorders. Here, we provide novel understanding of cardiac physiological impairment, amyloid pathology, neurotrophic factors loss, and impoverishment of cardiac neuronal fibers in Tg2576‐AD mice hearts, human cardiomyocytes in culture, and human AD post‐mortem left ventricular (LV) heart tissue. We reveal that Tg2576 animals exhibit increased myocardial fibrosis, amyloid β (Aβ) deposition, and brain/heart‐axis neurotrophic deficiencies, resulting in myocardial denervation and cardiac dysfunction. Aβ oligomers challenge reduces BDNF expression in both human immortalized and iPSC‐derived cardiomyocytes, by disrupting TrkB/CREB signaling. Analysis of human LV AD post‐mortem tissue confirms cell and animal results. Our findings reveal potential pathways by which Aβ pathology may disrupt cardiac neurotrophic signaling and physiology, identifying a possible link between AD and heart degeneration.

## Graphical abstract

This research identified cardiac amyloid pathology, neurotrophic factor depletion, and reduced myocardial nerve function in a transgenic model of cerebral amyloidosis (Tg2576), Aβ‐challenged cardiomyocytes, and in human AD heart tissue. These findings carry significant diagnostic and therapeutic implications, emphasizing the role of neuro‐signaling disruption in cardiac physiology impairment linked to AD. Our study advocates for considering cardiac complications in AD management and paves the way for future precision medicine approaches to enhance systemic clinical strategies for treating AD, proposing the cardiac neurotrophic signaling pathway as a potential therapeutic target.

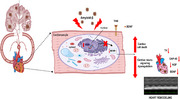

## 1 Introduction

Alzheimer's disease (AD) is an escalating global health concern, anticipated to triplicate in prevalence by 2050, primarily due to population aging [1]. This complex neurodegenerative disorder is characterized by the progressive accumulation of insoluble amyloid‐β (Aβ) plaques in the brain parenchyma together with neurofibrillary tangles formed by hyperphosphorylated tau. Besides Aβ plaques and tau buildups, about 90% of AD patients also show Aβ deposits in and around the cerebral vasculature, known as cerebral amyloid angiopathy (CAA). CAA contributes significantly to the progression of AD pathology by promoting neuroinflammation, neurovascular dysfunction, decreased brain clearance, increased blood‐brain barrier (BBB) permeability, and eventually neurodegeneration [2, 3].

Despite the main symptoms of the disease predominantly concerning the central nervous system (CNS), emerging evidence highlights extracerebral implications of AD, involving peripheral organs such as the heart [4]. Although abundant attention has been given to the impact of cardiovascular risk factors on AD risk and progression [5, 6, 7], only recent studies suggest a direct effect of AD pathology on cardiac dysfunction, and reveal Aβ peptides (Aβ40 and Aβ42) and big tau accumulation in the cardiac tissue of AD patients [8, 9].

The accumulation of Aβ in the AD brain is known to significantly affect the expression of the two main neurotrophic factors, nerve growth factor (NGF) and brain‐derived neurotrophic factor (BDNF) [10]. Numerous studies have demonstrated that AD pathology and specifically Aβ oligomeric aggregates are causal to the decreased NGF expression in basal forebrain cholinergic neurons and reduced BDNF levels in hippocampal and cortical regions, changes strongly linked to synaptic loss, neuronal vulnerability, and neuroinflammation [10, 11, 12, 13]. This neurosignaling pathway's maladaptive remodeling is associated with cognitive decline [14]. The production of neurotrophins such as BDNF is also important for peripheral organs and cells, including endothelial cells, muscle cells, and cardiomyocytes. BDNF operates through tropomyosin receptor kinase B (TrkB) stimulation, contributing to the activation of neuronal pro‐survival genes, including BDNF itself, through cAMP response element‐binding protein (CREB) signaling [15]. Additionally, BDNF exerts pleiotropic effects in endothelial cells, muscle cells, and cardiomyocytes. BDNF/TrkB/CREB signaling is crucial for proper heart development and function in adulthood [16]. It is plausible to hypothesize that the cerebral neurotrophic signaling degeneration in AD may be accompanied by a gradual decline also in peripheral levels and function of these neuromodulators. A lack of neurotrophic support may result in a severe derangement of the cardiac nervous system, potentially culminating in severe heart dysfunction. Notably, declining circulating BDNF levels have also been linked to cardiac disease progression, particularly of ischemic origin [17].

However, the effects of AD pathology on heart pathophysiology remain poorly understood, and whether and through what mechanisms AD and amyloidosis modulate NTFs levels and innervation in the cardiac tissue have not been previously explored. Also, whether specific Aβ aggregates such as toxic oligomers, which may accumulate in the AD heart, fuel cardiac neuro‐signaling dysregulation, is still to be defined.

To address these questions, we analyzed whether and how the accumulation of cardiac Aβ aggregates affected peripheral innervation and NTFs expression in the heart of Tg2576 transgenic mice, one of the most widely used models of cerebral amyloidosis. We observed a progressive decline in cardiac function, that became significant in 13 months‐old Tg mice compared to controls, and was accompanied by Aβ deposition, fibrosis, an increase in cardiac mass, and reduced contractility. We found a significant reduction in cardiac nerve fiber density and neurotrophin levels, adrenergic and neuronal fiber regeneration markers, in Tg2576 mice compared to controls. Through in vitro experiments on human AC16 cardiomyocytes, neuroblastoma cells, and validation in human‐induced pluripotent Stem Cell‐derived cardiomyocytes (hiPSC‐CMs), we showed that exposure to Aβ oligomers significantly reduced the expression of BDNF in both neural and cardiac cells. BDNF acts through tropomyosin receptor kinase B (TrkB) stimulation, contributing to the activation of pro‐survival genes, including BDNF itself, through cAMP response element‐binding protein (CREB) signaling. We demonstrated that CREB levels were reduced in cardiomyocytes exposed to Aβ oligomers, and that a TrkB agonist could mitigate caspase 3 activation and apoptosis in Aβ‐exposed cardiomyocytes. Finally, we validated these findings by analyzing the neurotrophic cardiac profile in human left ventricle (LV) post‐mortem tissue from AD patients with CAA compared to age‐matched healthy subjects, thus opening novel avenues for future investigations into the impact of AD pathology on cardiac physiology.

This study demonstrates that pathological Aβ pathology is associated with cardiac neuro‐signaling impairment in mouse models of AD and human AD hearts. Moreover, our in vitro studies directly challenging cardiomyocytes with Aβ oligomers suggest a plausible molecular mechanism that may contribute to Aβ‐mediated cardiac neurotrophic deficiency and nerve fiber loss in AD. Together, these findings contribute to filling an important gap in the understanding of the effects of AD on cardiac function and pathology.

## 2 Results

### 2.1 AD Pathology Promotes Cardiac Dysfunction, Aβ Accumulation, and Misfolding of Serum Amyloid A3 in the Heart of Tg2576 Mice

**FIGURE 1:**
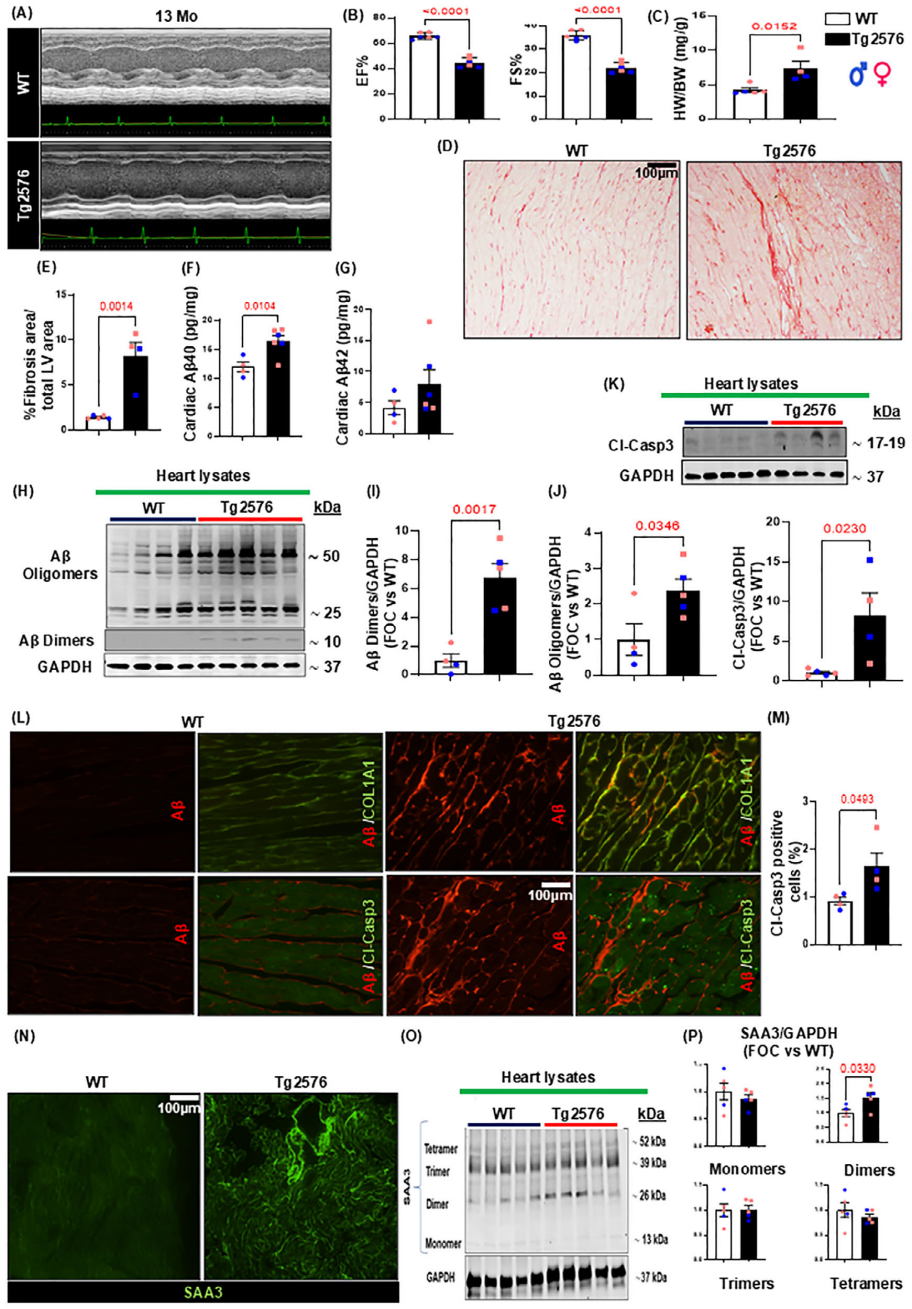
Cardiac physiological impairment, myocardial Aβ aggregates accumulation, cardiac cell apoptosis, and Serum amyloid A3 (SAA3) deposition in the heart of Tg2576 AD mice. (A) M‐mode representative images of echocardiographic analysis in 13‐month‐old WT and Tg2576 mice. (B) Left ventricle (LV) function is impaired in the AD model, as evidenced by a significant decline both in ejection fraction (EF%) and fraction shortening percentage (FS%) showed by the Tg2576 group compared to WT mice age‐matched (13‐months‐old). (n=6 vs. n=6). Student's t‐test P value <0.0001; Holm Sidak adjusted P value 0.0002. (C) The heart weight/body weight ratio is significantly increased in Tg2576 mice compared to the WT group. (n=5 vs. n=4). Student's t‐test P value 0.0152. (D,E) Representative images (left panels) and quantitative data (right panels) showing the percentage (%) of fibrotic area in cardiac sections from WT and Tg2576 mice, assessed via Picrosirius red staining (scale bar 50 µm). (n=5 vs. n=4). Student's t‐test P value 0.0014. (F,G): Aβ40 (F) and Aβ42 (G) cardiac levels (pg/mg of proteins), assessed by ELISA assays, in total cardiac lysates from WT and Tg2576 mice. (n=4 vs. n=6). Student's t‐test P value 0.0104; Holm Sidak adjusted P value 0.0206. (H–J): Representative immunoblots (left panels) and densitometric quantitative analysis (right panels) showing protein levels of Aβ dimers, oligomers (I,J) in total cardiac lysates from WT and Tg2576 mice. (n=4 vs. n=5). Student's t‐test P value 0.0017; Holm Sidak adjusted P value 0.0034. GAPDH levels were used as a loading control. (K): Representative immunoblots (upper panels) and densitometric quantitative analysis (lower panels) showing protein levels of Cleaved‐Caspase3 (Cl‐Casp3) in total cardiac lysates from WT and Tg2576 mice. (n=5 vs. n=4). Student's t‐test P value 0.0230. GAPDH levels were used as a loading control. (L): Representative digital images (upper panels, scale bar 100 µm) showing collagen type I fibers (COL1A1, in green) and Aβ (in red) and representative digital images (lower panels; scale bar 100 µm) and quantification (M) showing Cleaved‐Caspase3 (Cl‐Casp3, in green) and Aβ (in red) in cardiac sections from WT and Tg2576 mice. (n=4 vs. n=4). Student's t‐test P value 0.0493. (N): Representative WT and Tg2576 cardiac sections stained for serum amyloid A3 (SAA3, in green, scale bar 100 µm). (O,P): Representative immunoblots (O) and densitometric quantitative analysis (P) showing levels of different aggregates of SAA3 in total cardiac lysates from WT and Tg2576 mice. (n=5 vs. n=5). Student's t‐test P value 0.0330; Holm Sidak adjusted P value 0.1256. GAPDH levels were used as a loading control. Data are presented as a mean±SEM.

To assess the impact of AD amyloid pathology on cardiac tissue physiology and systolic activity, we conducted echocardiographic analyses in both male and female Tg2576 transgenic mice and age‐matched WT littermates, both before and after the development of cerebral Aβ pathology (at 4 and 8, or 13 months, respectively). Bi‐dimensional M‐mode tracings revealed a significant decrease in left ventricular ejection fraction (EF%) and fractional shortening (FS%) percentage in 13‐month‐old Tg2576 mice compared to WT animals (Figure 1A,B, and other cardiac echocardiographic parameters in Table S1). No detectable difference in diastolic measurements, as assessed by conventional echocardiography coupled with pulsed wave Doppler, was found between experimental groups (Figure S3). Moreover, radial and longitudinal strain/strain rates were also analyzed in both animal groups. Tg2576 exhibited global (average of 6 LV segments) and anterior‐apical longitudinal strain impairment compared to WT mice, with severe longitudinal contractility dysfunction that affects the anterior‐basal segment (Figure S2). Younger Tg2576 cohorts (at 4 or 8 months) did not exhibit significant changes in cardiac physiology (Figure S4), despite the presence of Aβ deposits in the brain starting at 8 months (Figure S6B,C). In line with this, no differences in cardiac BDNF expression were observed between WT and Tg‐AD mice at 8 months of age, before significant cardiac Aβ accumulation (Figure S6A). These molecular findings aligned with the echocardiographic analyses performed at the same age (Figure S4C,D). Collectively, these results suggest that BDNF reduction in the heart does not precede Aβ deposition, but instead appears to emerge only once cardiac amyloid pathology has become established. Furthermore, 13‐month‐old transgenic mice displayed notable left ventricle enlargement, evidenced by increased left ventricular end‐systolic diameter, leading to elevated left ventricular end‐systolic volume (LVESV, Table S1). The gravimetric analysis confirmed a significant increase in the heart weight/body weight ratio in 13‐month‐old Tg2576 mice compared to the WT group (Figure 1C), further corroborated by the increased cardiomyocyte cross‐sectional area assessed through WGA staining (Figure S1). Cardiac dysfunction in the Tg2576 mice coincided with a marked increase in myocardial interstitial fibrosis, as evidenced by Picrosirius red staining (Figure 1D,E), accompanied by amyloid β accumulation in Tg2576 myocardial parenchyma (Figure 1L) that contributes to a cardiac stiffness enhancement. Immunostaining results were confirmed via WB analysis, which shows high levels of fibronectin, a key component of the extracellular matrix (ECM) [18], in the Tg2576‐AD mouse heart (Figure S1). However, in line with another recent AD study [19], no significant changes in cardiac MMP‐2 levels between the WT and transgenic AD mice were observed (Figure S1).

Using peptide‐specific ELISA assays, we confirmed a significant increase in Aβ40 levels in 13‐month‐old Tg2576 cardiac tissue compared to WT mice, while no substantial changes in Aβ42 levels were noted between the two groups (Figure 1F,G). These findings align with the age dependent Aβ pathology in the Tg2576 mouse model, characterized by higher abundance and progressive increase in cerebral Aβ40 levels compared to Aβ42 [20]. Western blot analysis further validated the cardiac Aβ40 deposition detected by ELISA, confirming a significant increase in Aβ dimers and oligomers in total heart lysates of 13 months old Tg2576 mice compared to WT littermates (Figure 1H–J). Immunofluorescence staining revealed amyloid aggregates accumulating in interstitial spaces between cardiomyocytes, alongside collagen fibers, corroborating the myocardial interstitial fibrosis observed via Picrosirius red staining (Figure 1L, top panels, and Figure S8B). Besides Aβ aggregates, reports have demonstrated amyloid precursor protein (APP) expression in peripheral organs, including the myocardium, suggesting the potential production of Aβ outside the brain parenchyma [9, 21, 22]. Consistent with this, but also with the fact that the prion protein promoter may be expressed in other innervated tissue, including in the heart, we observed APP upregulation in the plasma and cardiac tissue of Tg2576 mice (Figure S9). Aβ challenge has been linked to increased apoptosis in various cell types, including cardiac cells, and in AD brains [23, 24]. Indeed, analysis of cardiac tissue by western blotting and immunohistochemistry revealed elevated levels of cleaved‐caspase 3 in Tg2576 cardiomyocytes (Figure 1K,L bottom panels, 1 M).

AD pathology is characterized by progressive protein misfolding, and interactions among different amyloid species may contribute to these processes [25]. The accumulation of misfolded Aβ oligomers in the heart tissue may contribute to increased fibrosis and could affect the behavior of other amyloidogenic peptides, such as serum amyloid A (SAA) proteins. Although high levels of SAAs typically do not lead to fibrillar deposits, chronic inflammatory states, or potentially cross‐interactions with other amyloids (e.g., such as Aβ) might favor SAA accumulation in peripheral tissues [26, 27]. Indeed, we observed Aβ aggregates around cardiac vessels in aged Tg2576 mice, accompanied by infiltration of the vascular basal lamina (Figure S5, *bottom panels*, and Figure S8D), along with increased serum amyloid A3 (SAA3) vascular deposits (Figure 1N). Immunoblot analysis confirmed increased SAA3 dimers in the cardiac tissue of Tg2576 mice (Figure 1O,P).

Overall, these findings indicate that AD pathology detrimentally affects systolic function and cardiac tissue structure. Progressive interstitial and perivascular Aβ deposits contribute to cardiac cell apoptosis and aggregation of serum amyloid proteins, culminating in cardiovascular dysfunction. Notably, 8‐month‐old Tg2576 mice exhibited evident amyloid aggregate accumulation in the prefrontal cortex vasculature and cerebral parenchyma (Figure S6C), consistent with literature describing early Aβ deposition and CAA in the Tg2576 brain [20]. However, no significant changes were observed in Aβ accumulation in the hearts of 8‐months old Tg2576 mice compared to the older transgenic cohort (Figures S6B and S7A), confirming that Aβ pathology starts in the brain of these mice, followed by a later increase in cardiac Aβ levels.

### 2.2 Neurotrophic Signaling is Impaired in the Heart of AD Transgenic Mice

**FIGURE 2:**
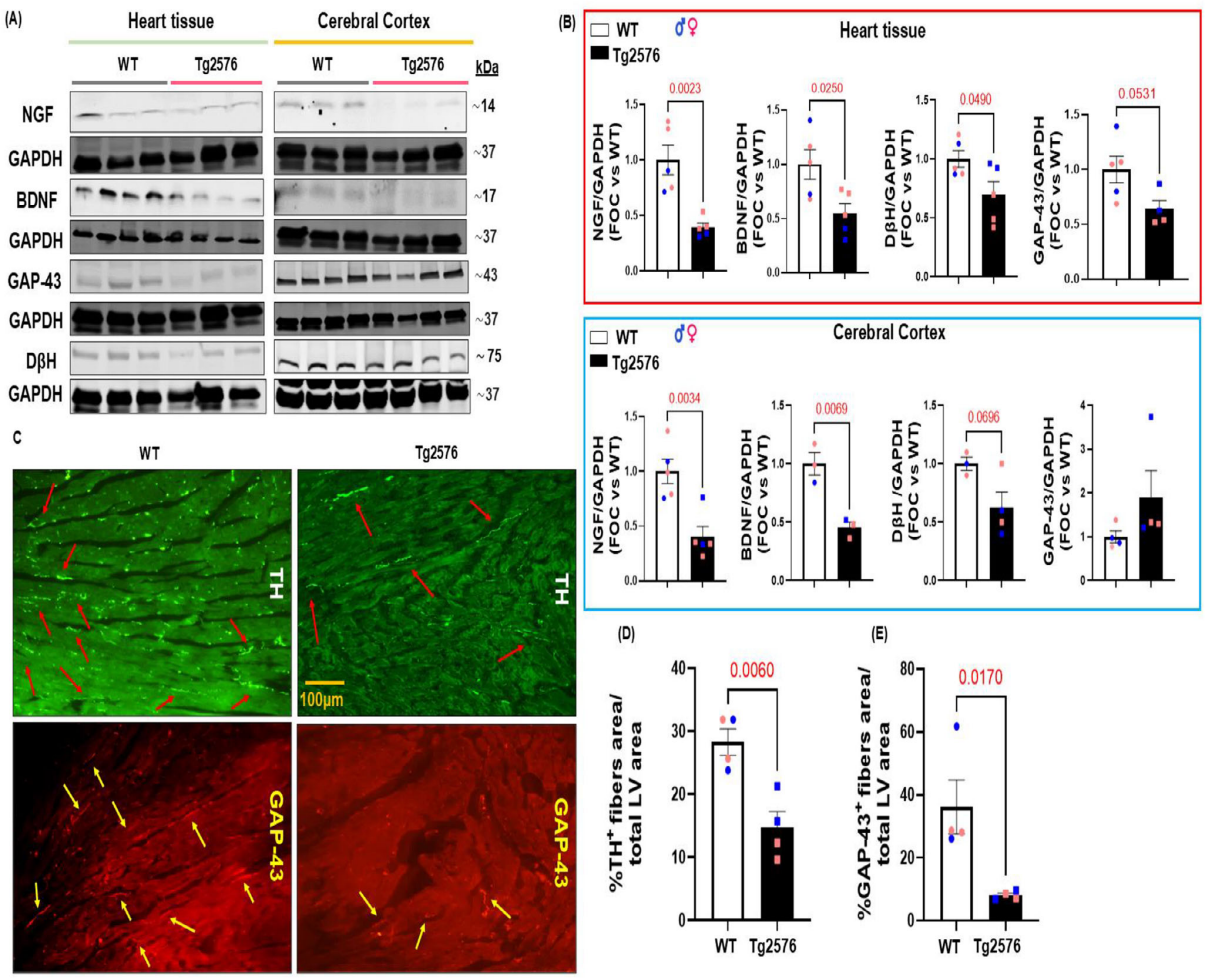
Alzheimer's disease pathology negatively impacts the neuro‐signaling pathway in the brain/heart axis of Tg2576 mice. (A,B): Representative immunoblots (A) and densitometric analysis (B) showing levels of NGF (n=5 vs. n=5, Student's t‐test P value 0.0023; Holm Sidak adjusted P value 0.0092), BDNF (n=5 vs. n=5, Student's t‐test P value 0.0250; Holm Sidak adjusted P value 0.0731), GAP‐43 (n=5 vs. n=4, Student's t‐test P value 0.0531; Holm Sidak adjusted P value 0.0531), and dβh (n=5 vs. n=5, Student's t‐test P value 0.0490; Holm Sidak adjusted P value 0.0956), in total heart tissue. In cerebral cortex lysates, NGF (n=5 vs. n=5, Student's t‐test P value 0.0034; Holm Sidak adjusted P value 0.0135), BDNF (n=3 vs. n=3, Student's t‐test P value 0.0069; Holm Sidak adjusted P value 0.0206), GAP‐43 (n=4 vs. n=4), and dβh (n=3 vs. n=4, Student's t‐test P value 0.0696; Holm Sidak adjusted P value 0.1343), from WT and Tg2576 mice. GAPDH levels were used as a loading control. (C–E): Digital images (C, scale bar 100 µm) and quantifications (D,E) showing cardiac adrenergic nerve fibers, labeled with anti‐tyrosine‐hydroxylase (TH, in green) (D), (n=4 vs. n=4, Student's t‐test P value 0.0060), and cardiac regenerating nerve endings, labeled with anti‐neuronal regeneration marker (GAP‐43, in red) (E), (n=4 vs. n=4, Student's t‐test P value 0.0170), in cardiac sections from WT and Tg2576 mice. Data are presented as a mean±SEM.

AD pathology and Aβ deposition are known to profoundly disrupt neurotrophin production in the brain, contributing to the progressive loss of key neuromodulators, including NGF and BDNF, ultimately leading to cognitive decline and dementia [28, 29]. However, whether AD pathology can modulate peripheral neurotrophic activity and cardiac innervation remained to be elucidated. Therefore, we explored the regulation of neuro‐signaling modulators and innervation factors in both cerebral cortex and cardiac lysates of 13‐month‐old Tg2576 mice. Immunoblotting analysis revealed a marked decrease in both cerebral and cardiac levels of NGF and BDNF expression in the Tg2576 group compared to WT littermates (Figure 2A,B). This reduction in neurotrophin levels observed in transgenic mice was accompanied by a significant decrease in the adrenergic marker (DβH) and the neuronal fiber regeneration marker, GAP‐43 (Figure 2A,B). Intriguingly, immunohistochemistry analysis corroborated a notable loss in cardiac nerve fiber density in Tg2576 mice compared to WT animals, including reductions in sympathetic and regenerating nerve endings labeled with TH and GAP‐43 markers, respectively (Figure 2C–E; Figure S8A–C). Importantly, under pathological conditions, neurotrophic signaling is known to shift, with downregulation of mature NGF and BDNF, as well as their Trk receptors, accompanied by upregulation of their immature isoforms, proNGF and proBDNF, and enhanced p75NTR‐mediated signaling. This pattern has been consistently reported in neurodegenerative disorders, including AD [30, 31]. Consistent with this, in our study Tg2576 hearts showed a marked reduction in mature NGF and BDNF measured by ELISA assays, accompanied by increased proBDNF levels, with proNGF unchanged (Figure S10), confirming the immunoblot results. Tg‐AD mice also exhibited a substantial decrease in cardiac TrkB phosphorylation, indicating impaired receptor activation despite ligand presence (Figure S11). This was accompanied by significant downregulation of total cardiac CREB and P‐CREB, further supporting TrkB dysfunction, and a notable upregulation of p75NTR in transgenic hearts (Figure S11). Together, these findings suggest that AD amyloid pathology may have detrimental effects on peripheral neurotrophic pathways, disrupting BDNF–TrkB–CREB signaling, and possibly increasing p75NTR pathological effects, thereby impacting the peripheral nervous system and cardiac innervation.

### 2.3 Aβ Oligomers Impair Neurotrophins Production in hiPSC‐CMs, AC16 Cardiomyocytes and Neuroblastoma Cells

**FIGURE 3:**
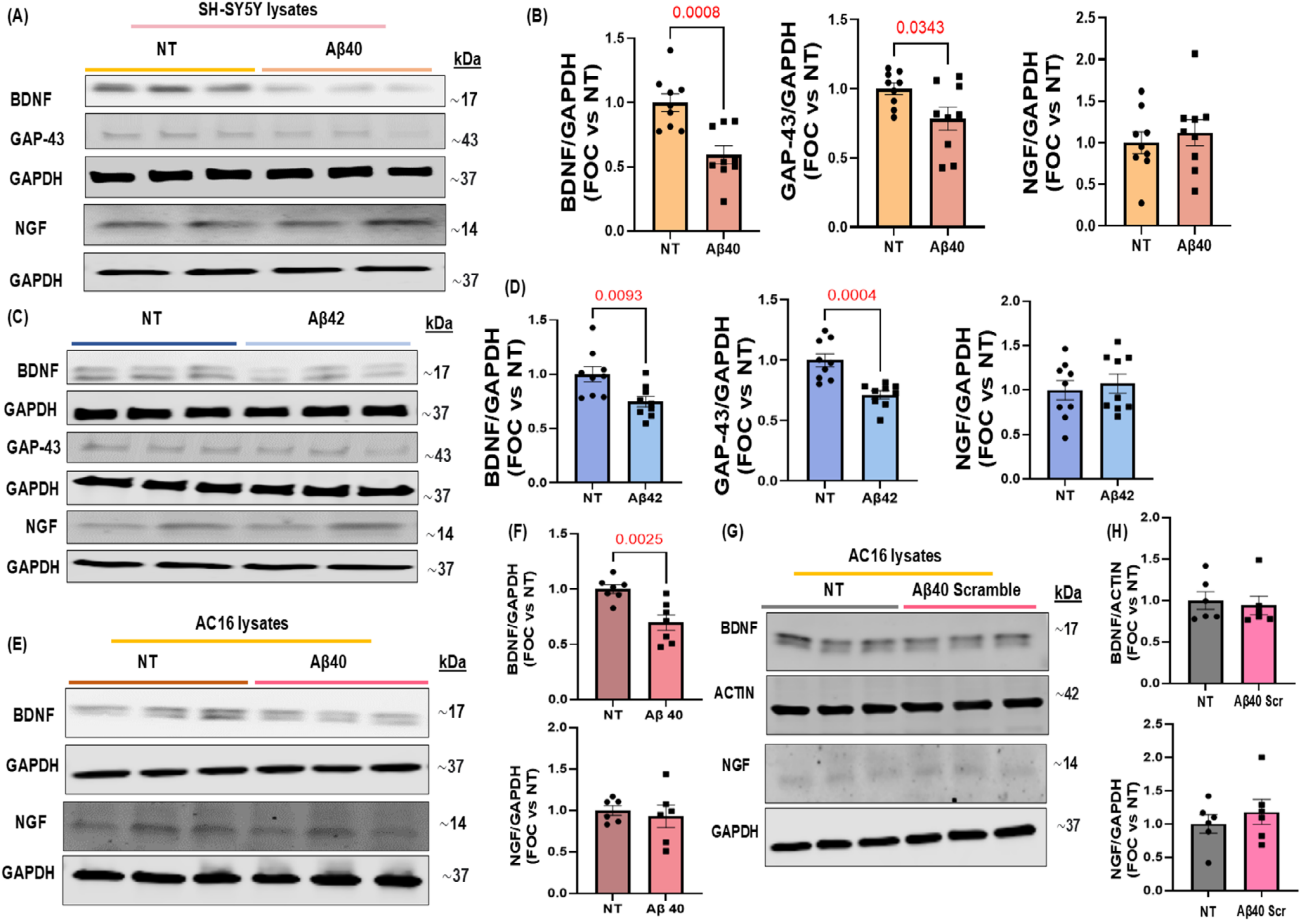
Effects of human Aβ40 or Aβ42 oligomers on neurotrophic factors in neuronal cells (SH‐SY5Y) and human cardiomyocytes (AC16). (A,B): Representative immunoblots (A) and densitometric analysis (B) of multiple independent experiments to evaluate BDNF (n=9 vs. n=9, Student's t‐test P value 0.0008; Holm Sidak adjusted P value 0.0024), GAP‐43 (n=9 vs. n=9, Student's t‐test P value 0.0343; Holm Sidak adjusted P value 0.0674), and NGF (n=9 vs. n=9, Student's t‐test P value 0.5642), protein levels (B) in human neuroblastoma cells (SH‐SY5Y) stimulated with human Aβ40 oligomers for 16 h. (C,D): Representative immunoblots (C) and densitometric analysis (D) of multiple independent experiments to evaluate BDNF (n=9 vs. n=9, Student's t‐test P value 0.0093; Holm Sidak adjusted P value 0.0185), GAP‐43 (n=9 vs. n=9, Student's t‐test P value 0.0004; Holm Sidak adjusted P value 0.0012), and NGF (n=9 vs. n=9, Student's t‐test P value 0.6389), protein levels (D) in human neuroblastoma cells (SH‐SY5Y) stimulated with human Aβ42 oligomers for 16 h. GAPDH levels were used as a loading control. (E,F): Representative immunoblots (E) and densitometric quantitative analysis (F) showing protein levels of BDNF (upper panel) (n=7 vs. n=7, Student's t‐test P value 0.0025; Holm Sidak adjusted P value 0.005), and NGF (lower panel) (n=6 vs. n=6, Student's t‐test P value 0.6470) in total protein lysates from human cardiomyocytes (AC16) stimulated with human Aβ40 oligomers for 16 h. GAPDH levels were used as a loading control. (G,H) Representative immunoblots (G) and densitometric quantitative analysis (H) showing protein levels of BDNF (upper panel) (n=6 vs. n=6, Student's t‐test P value 0.7035), and NGF (lower panel) (n=6 vs. n=6, Student's t‐test P value 0.4618), in total protein lysates from human cardiomyocytes (AC16) stimulated with scrambled Aβ40 for 16 h. ACTIN and GAPDH levels were used as a loading control. Data are presented as a mean±SEM. (NT= Not Treated).

**FIGURE 4:**
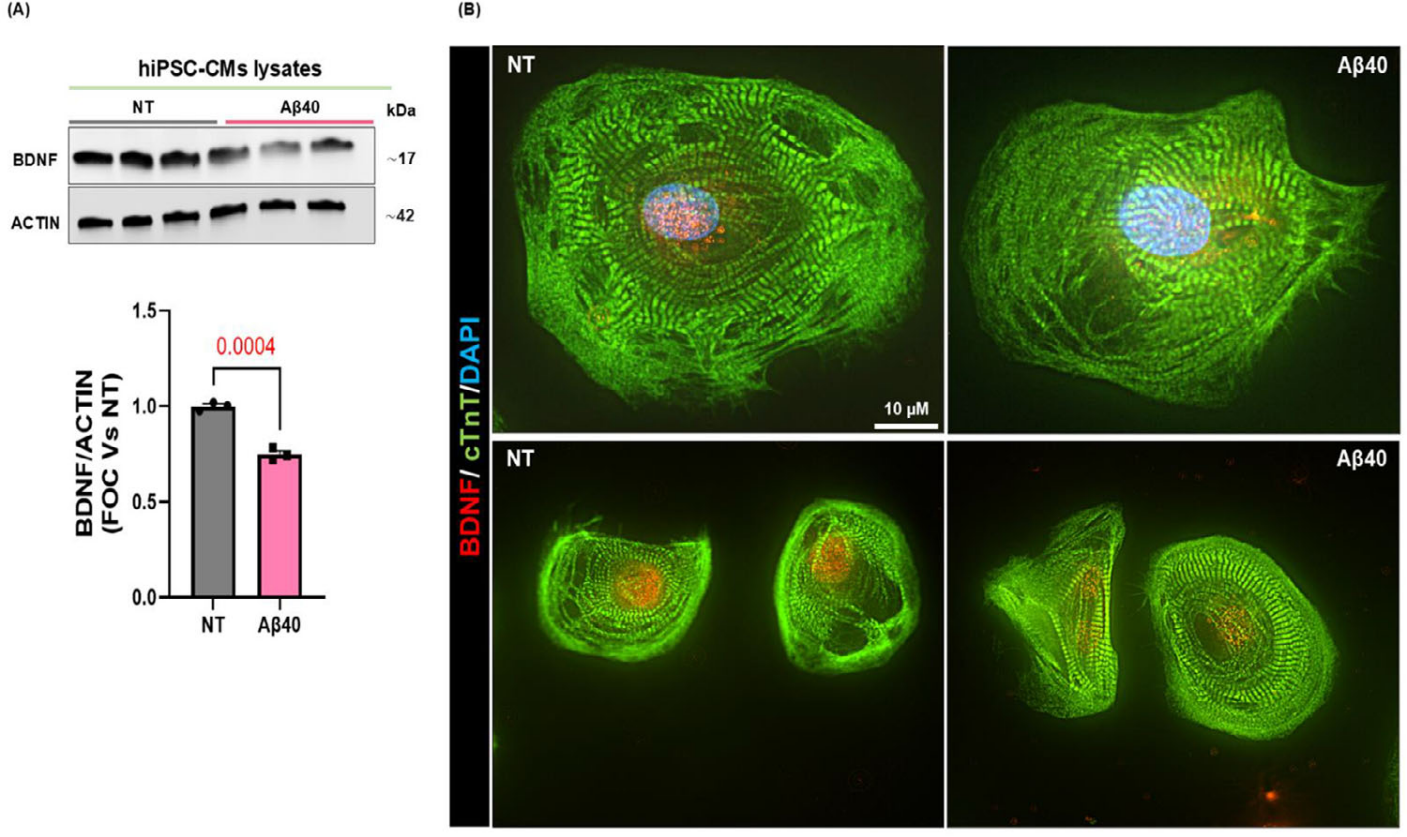
Aβ40 oligomers induce BDNF downregulation in human‐induced pluripotent stem cell‐derived cardiomyocytes (hiPSC‐CMs). (A) Representative immunoblots (upper panels) and densitometric quantitative analysis (lower panel) showing protein levels of BDNF (n=3 vs. n=3, Student's t‐test P value 0.0004), in total protein lysates from hiPSC‐CMs stimulated with human Aβ40 oligomers for 20 h. ACTIN levels were used as a loading control. (B) Representative digital images (scale bar 10 µm) of hiPSC‐CMs stained with BDNF (red), cardiac Troponin T (cTnT, in green), and DAPI (blue). Data are presented as a mean±SEM. (NT= Not Treated).

Our in vivo investigations revealed a notable decrease in neurotrophic markers and nerve fibers within the cardiac tissue of Tg2576 mice. To ascertain whether this loss of NTFs can be caused by the Aβ aggregates present in the heart, we evaluated the impact of Aβ40 oligomers, recognized as the most toxic amyloid aggregates and preferentially enriched in the AD brain vasculature, on the production of neurotrophins and neuronal regeneration markers in both human iPSC‐CMs and AC16 cardiomyocytes (Figures 3 and 4). Aβ40 oligomers significantly reduced BDNF expression in hiPSC‐CMs and AC16 cardiomyocytes (Figures 3E,F and 4). A scrambled Aβ40 peptide, used as a negative control, did not induce significant changes in BDNF or NGF levels in AC16 cardiomyocytes, compared with untreated cells (Figure 3G,H), thus corroborating that the observed responses may be specifically Aβ‐mediated. In addition, the effects of Aβ42 oligomers, the main toxic species coming in contact with neurons in the brain parenchyma, were examined on neuronal SHSY‐5Y cells. Following a 16‐h incubation with Aβ oligomers, a significant reduction in protein levels of GAP‐43 and BDNF was observed in neuronal cells, with no substantial changes in NGF expression (Figure 3A–D). These findings support the hypothesis that Aβ toxic aggregates contribute to the impairment of the cardiac neuro‐signaling and neural regeneration pathways by diminishing the production of BDNF in both neurons and cardiomyocytes and reducing levels of GAP‐43 in neurons.

### 2.4 Neurotrophic factors are reduced in the Heart of Alzheimer's disease patients with cerebral amyloid angiopathy

**FIGURE 5:**
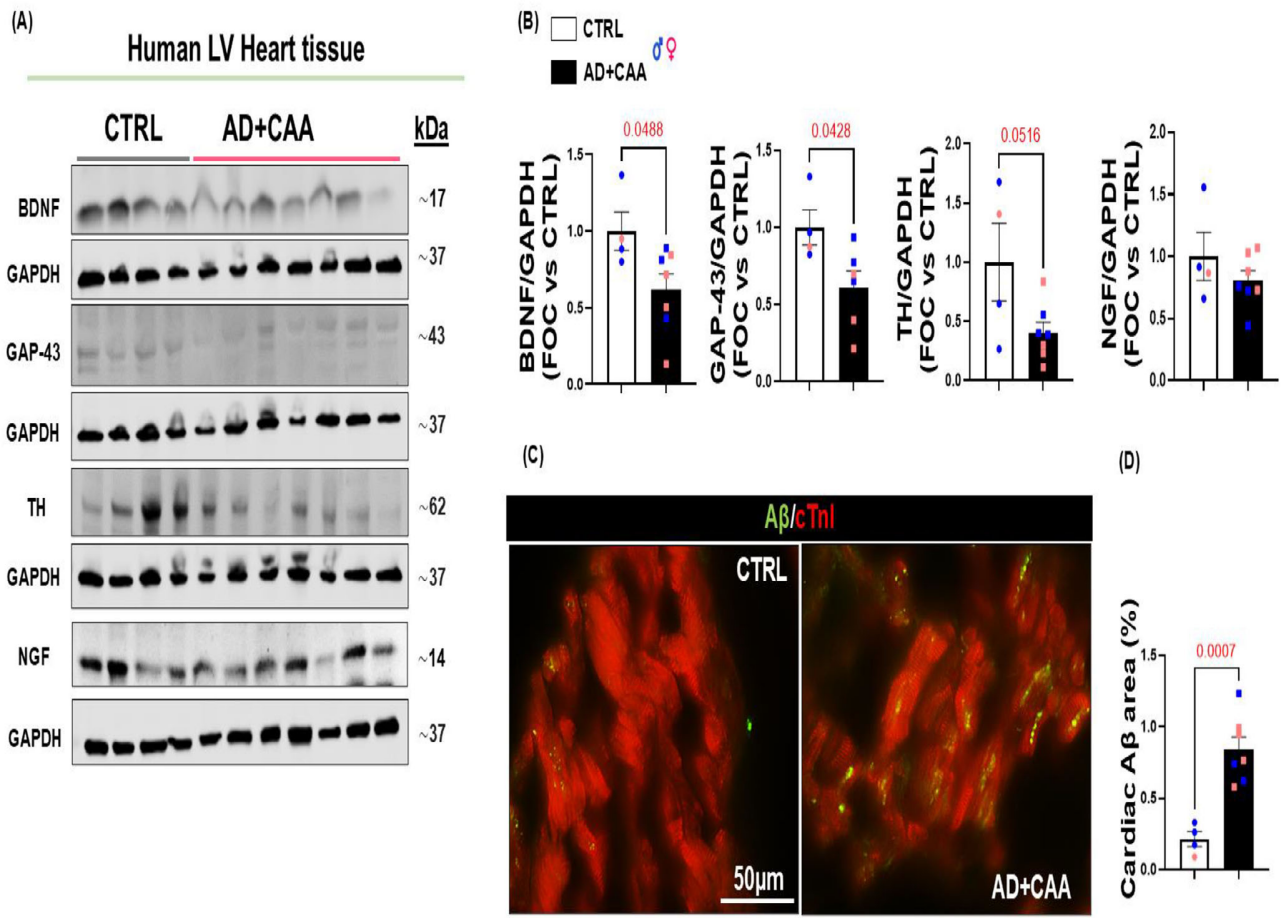
Myocardial neuro‐signaling impairment and Aβ deposits in human post‐mortem cardiac tissue of AD subjects. (A,B): Representative immunoblots (A) and densitometric analysis (B) showing reduced levels of BDNF (n=4 vs. n=7, Student's t‐test P value 0.0488; Holm Sidak adjusted P value 0.1605), GAP‐43 (n=4 vs. n=7, Student's t‐test P value 0.0428; Holm Sidak adjusted P value 0.1394), TH (n=4 vs. n=7, Student's t‐test P value 0.0516; Holm Sidak adjusted P value 0.1005), but not NGF (n=4 vs. n=7, Student's t‐test P value 0.3015), in total human LV lysates of patients with AD+CAA compared to healthy donors (CTRL). GAPDH levels were used as a loading control. (C,D): Representative digital images (C, scale bar 50 µm), and quantification (D) of the LV sections from CTRL and AD+CAA subjects stained with Aβ aggregates (in green) and cardiac Troponin I (cTnI, in red). (n=4 vs. n=7, Student's t‐test P value 0.0007). Data are presented as a mean±SEM.

To confirm the clinical and translational relevance of our findings in human AD, we investigated the cardiac neurotrophic profile in LV biopsies obtained from patients affected by AD along with CAA. Our study cohort comprised individuals clinically diagnosed with AD, confirmed through anatomopathological examination, excluding cases affected by other conditions that could significantly impair cardiac function, such as high blood pressure, secondary amyloidosis, stroke, brain and cardiac neoplastic conditions, prior myocardial infarction, and endocarditis. Healthy volunteers and AD+CAA patients were matched based on age, sex, and ethnicity (Table 1). Our analysis revealed significantly reduced levels of BDNF, downregulation of the sympathetic marker tyrosine hydroxylase (TH), and a decline in GAP‐43 protein expression in the heart of AD+CAA subjects, confirming the deterioration of neurotrophic factors and the neuro‐signaling pathway in the human AD heart (Figure 5A,B), with enormous clinical implications. Immunostaining analysis showed amyloid‐β deposits in the myocardial parenchyma of human AD patients with CAA (Figure 5C). In aggregate, these findings demonstrate a myocardial neuro‐signaling impairment associated to Aβ deposition in human AD hearts, supporting our experimental in vitro and in vivo results.

**TABLE 1:** Anatomopathological and demographic characteristics, medications, cause of death, and post‐mortem interval of the selected clinical cohort. (n=4 vs. n=7). Student's t‐test P value 0.1253.

Age	Ethnicity/Gender	Group	Associated Diseases	Medications	Cause of death	PMI (hours)
53	CM	CTRL	Adenocarcinoma; Status post‐Whipple procedure and liver transplantation; Hydroceles; Pulmonary vascular congestion; Hepatic congestion	N/A	Cholangiocarcinoma	4
75	CM	CTRL	Renal collecting duct carcinoma; Status post‐Whipple procedure with gastric bypass and cholecystectomy; Mild cardiomegaly; Aorta atherosclerosis; Testicular atrophy	Hydroxychloroquine, methotrexate, folic acid, prednisone, ferrous sulfate, acetaminophen‐hydrocodone, etodolac, simvastatin, omeprazole, clopidogrel, aspirin	Renal cell carcinoma with metastases	3
81	CM	CTRL	Prostatic adenocarcinoma; Bilateral end‐stage lung with honeycombing fibrosis (idiopathic pulmonary fibrosis with bronchiectasis by history); Cardiomegaly with left ventricular myocardial hypertrophy; Hepatomegaly; Hepatitis; Acute tubulointerstitial nephritis	Atenolol, fluoxetine, omeprazole, atorvastatin, bicalutamide, terazosin, prednisone, albuterol, aspirin, multivitamin, acetaminophen‐diphenhydramine, calcium carbonate, ergocalciferol, multivitamin	Acute respiratory failure due to idiopathic pulmonary fibrosis	2
90	CF	CTRL	Bilateral renal atrophy with mild nephrosclerosis and cortical cysts; Generalized atherosclerosis; Chronic esophagitis; Cardiomegaly with left ventricular myocardial hypertrophy; Hepatic centrilobular congestion; Chronic thyroiditis	Ferrous sulfate, goserelin, epoetin alfa, omega‐3 polyunsaturated fatty acids, methocarbamol, senna, lorazepam, docusate lysine	Congestive heart failure, mitral stenosis	7
74	CF	AD+CAA	Clear cell renal cell carcinoma; Cardiomegaly with left ventricular myocardial hypertrophy; Atherosclerosis; Thoracic and abdominal aorta; Pulmonary vascular congestion; Hepatic atrophy; Status post thyroidectomy; Status post hysterectomy and bilateral salpingo‐oophorectomy	Benazepril, levothyroxine, multivitamin, folic acid, calcium‐vitamin D, vitamin E, ascorbic acid, zinc sulfate, doxazosin, metoprolol, cyanocobalamin, aspirin, valsartan, ginkgo, chondroitin‐glucosamine, donepezil, acetaminophen, calcium carbonate, hydralazine‐hydrochlorothiazide, amoxicillin	Respiratory arrest, supranuclear palsy with dementia	5
85	CM	AD+CAA	History of prostatic carcinoma; Status post prostatectomy; Aortic atherosclerosis; Left ventricular myocardial hypertrophy; Pulmonary fibrosis and bronchial metaplasia; Chronic esophagitis; Benign renal cysts; Bilateral pancreatic and testicular atrophy; Thyroid nodular goiter; Status post cholecystectomy and Status post appendectomy	Doxepin, meclizine, donepezil, allopurinol, fluticasone, aspirin, multivitamin, chondroitin‐glucosamine, folic acid, lisinopril, ascorbic acid, vitamin E, memantine, multivitamin‐minerals, diphenhydramine	Alzheimer's dementia	3
86	CF	AD+CAA	Acute bronchopneumonia; Mild cardiomegaly with left ventricular myocardial hypertrophy; Aortic atherosclerosis; Benign endometrial polyp; Nodular goiter; Osteopenia; Hepatic chronic passive congestion; Enchondroma; lumbar vertebra; Renal cortical cysts; History of plasmacytoma	Albuterol, beclomethasone, conjugated estrogens, calcitonin, levothyroxine, multivitamin, ascorbic acid, calcium carbonate, salmeterol, ranitidine, metoclopramide, budesonide, citalopram, Al hydroxide/Mg hydroxide/simethicone, aspirin, acetaminophen, oxybutynin, escitalopram, magnesium chloride, vitamin E, polycarbophil, travoprost ophthalmic	Multiple myeloma	2.5
87	CM	AD+CAA	Acute bronchopneumonia; Pulmonary interstitial fibrosis; Cardiomegaly with left ventricular myocardial hypertrophy; Aortic atherosclerosis; Chronic pyelonephritis; Bilateral renal atrophy; Hepatic atrophy; Cholelithiasis; Parathyroid adenoma; S/P radical prostatectomy; history of prostate cancer; S/P Cardiac pacemaker placement	N/A	End‐stage Alzheimer's disease	2.5
87	CM	AD+CAA	Acute bronchopneumonia; Aortic atherosclerosis; Hepatic chronic passive congestion; Prostatic glandular hyperplasia; Testicular atrophy; Mild chronic thyroiditis; Status post Cardiac pacemaker; Diverticulosis; Benign renal cortical cyst; right	Warfarin, metoprolol, lisinopril, digoxin, gabapentin, terazosin, finasteride, niacin, bethanechol, omeprazole, Al hydroxide/Mg hydroxide/simethicone, hydroxyzine, acetaminophen‐codeine, donepezil, valproic sodium, cyanocobalamin, albuterol; divalproex sodium	Aspiration pneumonia, vascular dementia	2
89	CF	AD+CAA	Acute bronchopneumonia; Calcified granuloma; Aortic atherosclerosis; Pleural fibrotic plaque; Mild calcification; Aortic valve; Atherosclerotic stenosis; Cardiomegaly; Hepatic atrophy; Mild chronic lymphocytic thyroiditis; Status post hysterectomy and bilateral salpingo‐oophorectomy	Estrone, metoprolol, aspirin, APAP/ASA/caffeine, amoxicillin, glycerin, multivitamin, ascorbic acid, vitamin E, calcium carbonate, hydrochlorothiazide‐triamterene, tamoxifen, levothyroxine, exemestane, triamterene, nitroglycerin, citalopram, mirtazapine, bupropion, folic acid, warfarin, rivastigmine, memantine, cyanocobalamin	Failure to thrive	3
90	CF	AD+CAA	Pancreatic ductal adenocarcinoma; Cardiomegaly; Atherosclerosis; thoracic and abdominal aorta; Emphysema; Diverticulosis; Hashimoto's thyroiditis; Status post supracervical hysterectomy; Benign atrophic and fibrotic breast tissue; Osteopenia	Rofecoxib, clonazepam, calcium carbonate, diphenhydramine, hydrochlorothiazide‐triamterene, calcium carbonate, hydrochlorothiazide‐triamterene, vitamin E, diphenhydramine ranitidine, clonazepam, ginkgo, acetaminophen, aspirin calcium carbonate, risperidone, escitalopram	Failure to thrive, cholangitis, presumed pancreatic neoplasm	2

Abbreviations: CF = Caucasian Female, CM = Caucasian Male, Ctrl = Control, AD = Alzheimer's Disease, CAA = Cerebral Amyloid Angiopathy, PMI = post‐mortem interval.

### 2.5 Aβ40 Oligomers Impair BDNF/TrkB/CREB Signaling in Human Cardiomyocytes

**FIGURE 6:**
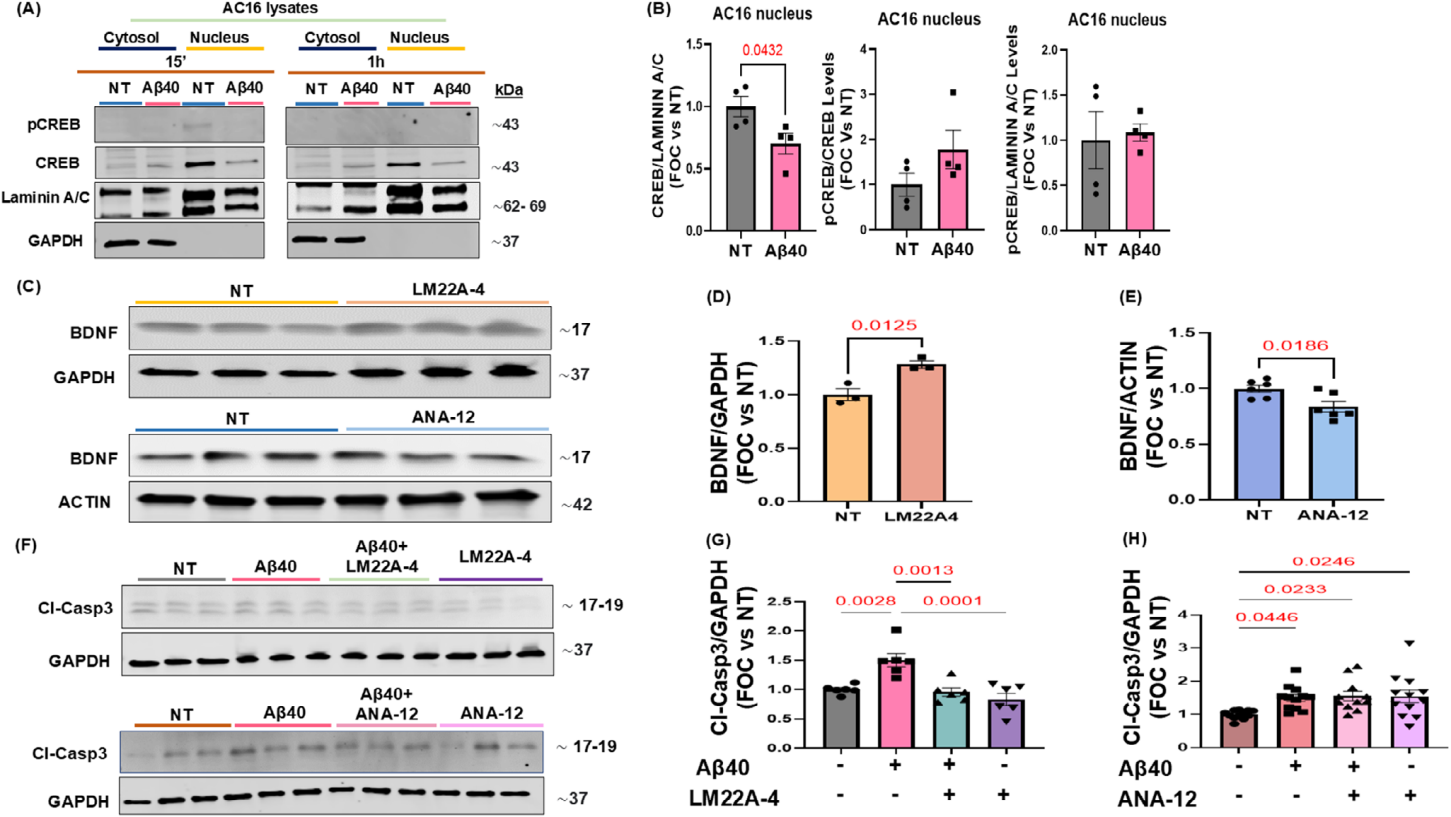
CREB signaling is impaired by Aβ40 oligomers, and a TrkB agonist reduces Aβ40‐mediated caspase 3 activation in human cardiomyocytes (AC16 cells). (A,B): Representative immunoblots (A) and densitometric quantitative analysis (B) of multiple independent experiments to evaluate pCREB and CREB levels (n=4 vs. n=4, Student's t‐test P value 0.0432; Holm Sidak adjusted P value 0.1241), in nuclear extracts isolated from human cardiomyocytes (AC16) stimulated with human Aβ‐40 oligomers for 15 min or 1 h. LAMININ A/C levels were used as a nuclear loading control. (C–E): Representative immunoblots (C) and densitometric quantitative analysis (D,E) showing protein levels of BDNF in total protein lysates from human cardiomyocytes (AC16) stimulated with TrkB agonist LM22A‐4 (100 nM) (n=3 vs. n=3, Student's t‐test P value 0.0125); or TrkB antagonist ANA‐12 (30 µM) (n=6 vs. n=6, Student's t‐test P value 0.0186), for 16 h. GAPDH or ACTIN levels were used as loading controls. Data are presented as a mean±SEM. (F–H): Representative immunoblots (F) and densitometric quantitative analysis (G,H) of multiple independent experiments to evaluate Cleaved‐Caspase 3 (Cl‐Casp3) in AC16 cells stimulated with human Aβ‐40 oligomers for 16 h. Before Aβ‐40 oligomers treatment, cells were pretreated with either LM22A‐4 (100 nM, top panel), or ANA‐12 (30 µM, bottom panel) for 1 h. GAPDH levels were used as a loading control. n=6‐12. One‐way ANOVA with Tukey's multiple comparisons post hoc test: *P<0.05 and vs NT. (NT= Not Treated).

The downregulation in BDNF expression observed in our in vitro and in vivo experiments prompted us to investigate the mechanism by which Aβ affects the cardiac BDNF pathway. BDNF/TrkB/CREB signaling impairment is involved in age‐related synaptic deterioration and neurodegenerative disorders, culminating in cognitive decline and neuronal loss. Indeed, AD patients exhibit significant depletion of BDNF expression in cerebrospinal fluid, post‐mortem brain tissue, and bloodstream [32]. The detrimental impact of Aβ oligomers on BDNF exon IV transcript in neuronal cells is associated with decreased CREB levels, the critical mediator of BDNF transcription [33, 34]. Consistent with these findings, here we observed a significant reduction in nuclear CREB levels in human cardiomyocytes following treatment with Aβ40 oligomers (Figure 6A,B), suggesting that a reduction in CREB transcriptional function mediates both the reduced levels of BDNF and the impact on the other neurotrophic markers. These results support a direct role of Aβ challenge on the BDNF/CREB axis. To confirm the protective effect of TrkB activation in our model, we stimulated human cardiomyocytes with the selective TrkB receptor agonist, LM22A‐4 [35] (Figure 6C,D), in the presence or absence of Aβ40 oligomers [36]. Remarkably, LM22A‐4 stimulation reduced Aβ40‐mediated caspase 3 activation, thereby mitigating apoptosis, and confirming the protective effects of BDNF signaling on cardiac cells (Figure 6F,G). Conversely, ANA‐12, a specific BDNF/TrkB receptor antagonist [37] (Figure 6C–E), exacerbated cardiomyocyte caspase 3 activation, (Figure 6F–H). Overall, these findings shed light on the effects of Aβ on neurotrophic signaling pathology in the heart (Figure S12), highlighting a potential protective contribution of BDNF/TrkB/CREB signaling to cardiomyocyte homeostasis.

## 3 Discussion

This study demonstrates that, similarly to what was previously observed in the brain, Aβ deposition in the AD heart leads to neurotrophic signaling impairment, which contributes to increased cardiomyocyte apoptosis and severe cardiac morphological and functional impairment observed in the hearts of Tg2576 mice. Confirming the clinical validity of these findings, the neurotrophic changes and the presence of Aβ pathology were validated in human post‐mortem heart samples from patients with AD+CAA. Our data suggest that cardiac amyloidosis (evidenced by the presence of Aβ and SAA3 oligomers) contributes to interstitial fibrosis and to the loss of neurotrophins, resulting in cardiac innervation deficits and neuro‐signaling pathway impairment, which appear to be essential mediators of the observed cardiac physiological dysfunction. The heart‐brain axis constitutes a complex and highly regulated bi‐directional interconnection between the central and peripheral nervous systems [38]. Neurodegenerative pathologies, such as AD, may have severe and debilitating effects on various peripheral organs, including the heart, through the modulation of both the central and peripheral nervous systems. However, the impact of AD pathology and Aβ accumulation on cardiac neuro‐signaling pathways in AD patients and animal models has not been previously elucidated. An increasing number of studies are beginning to highlight the involvement of cardiac tissue in neurodegenerative diseases, revealing a compelling interplay between AD and cardiac disorders [39], such as atrial fibrillation [40], heart failure [41], and coronary artery disease [22]. It has recently been shown that AD patients exhibit myocardial dysfunction following the deposition of Aβ aggregates in cardiac tissue [9], and the presence of cardiac N‐terminal cleavage products of APP was correlated with levels of CAA and myocardial fibrosis [42]. In line with this, Sanna and coworkers described the presence of structural and functional abnormalities in the cardiac tissue of a small cohort of AD subjects [4].

AD pathology has been shown to severely impact the production and release of the main NTFs, NGF and BDNF, in the brain [43, 44]. These two neurotrophins are mainly involved in the maturation and differentiation of neurons, regulating neurogenesis and synaptogenesis [45, 46]. The progressive decline in NTFs expression due to aging, exacerbated by AD pathology, may lead to an adverse remodeling of the whole neuro‐signaling pathway, driving, together with other AD pathological factors such as amyloid oligomers, an impairment of the peripheral innervation [29].

This study reveals cardiac physiological, pathological, and neurotrophic impairments using a transgenic mouse model of AD with CAA, human cardiomyocytes in culture, including hiPSC‐derived cardiomyocytes, challenged with Aβ, and AD+CAA human post‐mortem heart tissue. It also describes pathways that may contribute to impaired peripheral innervation and reduced neurotrophin levels in the AD heart, providing evidence that Aβ oligomers can disrupt neurotrophic signaling in cardiomyocytes. We report that Tg2576 mice show an age‐dependent deterioration in cardiac function and a gradual replacement of viable myocardial with fibrotic tissue, resulting in interstitial fibrosis. This cardiac maladaptive remodeling, which develops after the brain Aβ deposition, is accompanied by an increased cross‐sectional area alongside a significant accumulation of Aβ, particularly Aβ40, localized in cardiac perivascular and interstitial spaces with collagen deposits. Caspase 3 activation within cardiomyocytes is also increased, suggesting that Aβ aggregates in the heart tissue contribute to the development of interstitial fibrosis and apoptotic cardiac cell death. The preferential accumulation of Aβ40, rather than Aβ42, in Tg2576 mice hearts is in line with previous studies describing the progressive increase mostly of Aβ40 levels in both serum and brain parenchyma of Tg2576 mice during aging [20]. Additionally, the Aβ40 peptide is preferentially associated with vascular deposits and CAA in the human brain, resulting in degenerative phenotypes in cerebral endothelial cells [24], which culminate in vessel wall dysfunction [23], contributing to BBB permeability and microhemorrhages [47]. This increased BBB permeability in AD brains may enhance the systemic diffusion of Aβ40 (including aggregated forms such as oligomers) through the blood and its accumulation in peripheral organs, including the heart. Indeed, Aβ40 is the most abundant peptide in the cerebrospinal fluid and blood of AD patients, and multiple biomarker studies have shown that this peptide does not decrease in peripheral organs and tissues with AD progression [48]. Conversely, as Aβ42 fibrils accumulate in the brain parenchyma, Aβ42 levels decrease in the cerebrospinal fluid and in the peripheral blood [49].

It is known that Aβ oligomers may influence the aggregation of other amyloid species through seeding processes [50]. Some of these aggregation species fuel cell stress and inflammation, resulting in degenerative processes in multiple cell types, including endothelial and cardiac cells, as our group and others have shown [51, 52]. Here, we show that SAA3 also accumulates in the heart of Tg2576 mice, likely contributing to amorphous and misfolded protein accumulation and to progressive cardiac impairment. SAA3 is involved in vascular injury as a promoter of atherosclerosis, and it is a prognostic factor for coronary artery syndromes, also proposed as a novel inflammatory marker for ischemic heart disorders [53, 54, 55]. Notably, we observed SAA3 aggregates accumulating primarily at the cardiac vessel walls, where also Aβ deposits are found, suggesting that misfolded Aβ peptides or oligomers may diffuse through the vascular system, reach the heart, and influence the behavior of other systemic amyloids surrounding the myocardial vessels, although more studies are needed to support a possible functional interaction Interestingly, no changes in cardiac physiology and no evidence of amyloid deposits were found in younger (4 or 8 months) Tg2576 mice. However, Aβ deposits were already detected around brain vessels in the prefrontal cortex of 8‐month‐old Tg2576 mice. Thus, it is reasonable to think that, in this model and presumably in AD patients, cardiac dysfunction may be subsequent to the progressive accumulation of amyloid aggregates in the brain, particularly as CAA, in and around the brain vessels. Given the extensive literature available on the vascular contributions to cognitive impairment (VCID) field [5, 56], we postulate that the cardiac disease and maladaptive heart remodeling resulting from progressive amyloid deposition in the heart also contribute to cognitive decline, thereby exacerbating AD pathology in a destructive feed‐forward loop.

Notably, we observed pro‐apoptotic markers (active caspase 3) in cardiomyocytes exposed to Aβ oligomers in vitro and in vivo, suggesting a direct effect of Aβ pathological aggregates on the impairment of cardiac cell function. Interestingly, caspase 3 activation is also associated with tau cleavage at position 421, which promotes increased tau phosphorylation [57], suggesting possible downstream effects that may precipitate tau pathology, which was recently described in the heart by the Del Monte group [8, 58]. Notably, we revealed a depletion in both adrenergic nerve endings and regenerated nerve terminal fibers in the transgenic AD mouse hearts. This myocardial denervation was associated with a reduction of NGF and BDNF at both the cerebral and cardiac levels, accompanied by significant impairment in the neuronal sprouting marker GAP‐43. Cardiac depletion of mature neurotrophins, coupled with upregulation of immature isoforms such as proBDNF, mirrors the well‐documented pattern in neurodegenerative diseases, including AD [11, 30, 31, 59]. Additionally, Tg2576 mice hearts exhibited increased p75NTR levels, suggesting Aβ‐mediated proapoptotic and toxic effects via p75NTR, also found to be directly activated by Aβ in neuronal cells [60, 61, 62]. These findings further corroborate our in vitro and in vivo evidence that AD‐related pathology may impair cardiac neurotrophic signaling. Importantly, post‐mortem AD human hearts also exhibited a reduction in BDNF expression, as well as in the cardiac nerve fiber markers TH and GAP‐43. In AD patients, the decline of neuromodulators in the brain is known to accelerate age‐related synaptic loss, resulting in cognitive impairment [63, 64]. NTFs are powerful biomolecules with significant pleiotropic effects involved in cellular growth and survival pathways in both the brain and peripheral cells/tissues, including endothelial cells, muscle cells, and the cardiovascular system [65]. While peripheral innervation contributes to BDNF delivery to the heart, our cardiomyocyte experiments indicate that local cardiac BDNF loss also accounts forthe impairments observed in our models [16, 66, 67]. Notably, exposure to Aβ oligomers significantly reduced BDNF and GAP‐43 expression in human neurons as well as in hiPSC‐derived and immortalized cardiomyocytes, suggesting that Aβ may diminish NTFs levels in peripheral neurons, cardiomyocytes, and possibly endothelial cells. Such reductions may cause AD patients to be more susceptible to developing cardiovascular disorders of various etiologies [68, 69]. In addition to Aβ, tau, another key hallmark of AD, may also disrupt cardiac neuro‐signaling. Clarifying the contribution of tau pathology to cardiac innervation will therefore be an important focus of future studies.

To further investigate the Aβ‐mediated neuro‐signaling deterioration, we pharmacologically modulated BDNF signaling in human cardiomyocytes. Cells treated with LM22A‐4, a competitive agonist of the BDNF receptor TrkB, showed a reduction in Aβ40 oligomers‐mediated caspase 3 activation, suggesting that a TrkB‐dependent pathway may link reduced BDNF to the Aβ‐mediated cardiomyocyte toxicity. LM22A‐4 was also reported to alleviate cardiac ischemia‐induced apoptosis and to improve neuronal outgrowth and myocyte physiology with endothelial cell proliferation, thus arresting chronic heart failure [17]. Moreover, LM22A‐4 showed effects similar to BDNF in preventing Aβ‐induced neuronal cell death [35]. These beneficial effects suggest the initiation of a myocardial autocrine/paracrine protective pathway through TrkB activation, which may include the increased transcription of BDNF itself, via CREB activation [17].

In line with this concept, we demonstrated a significant downregulation of CREB nuclear expression (necessary for its transcriptional function) in cardiomyocytes challenged with Aβ40 oligomers. CREB is the leading transcription factor activated upon BDNF‐TrkB binding, which also promotes, in a feed‐forward loop, the transcription of BDNF itself. Interestingly, Tg2576 mouse hearts displayed a marked reduction in TrkB phosphorylation, suggesting impaired TrkB activation despite ligand presence. This was accompanied by a significant downregulation of total CREB and P‐CREB, further supporting TrkB dysfunction and reinforcing our in vitro and in vivo evidence that AD‐related pathology may disrupt heart BDNF/TrkB/CREB signaling. Our results provide a mechanistic explanation of how Aβ oligomers can promote a CREB‐mediated downregulation of BDNF expression in cardiomyocytes, uncovering a previously unknown molecular pathway underlying the AD‐mediated cardiac neuro‐signaling dysregulation. Notably, we demonstrated that the cardiac tissue of AD patients with CAA exhibits significantly reduced neurotrophic markers, including BDNF, together with Aβ deposits within the myocardial tissue. The presence of Aβ aggregates was also previously revealed in the human AD heart [9], reinforcing the relevance of our findings.

To our knowledge, this is the first study that investigated the impact of Aβ pathology on the neurotrophic signaling pathway in the AD heart and its effect on cardiovascular physiology in an AD animal model, with a validation of the loss of neurotrophic signaling mediators in human AD post‐mortem cardiac tissue. These findings uncover a previously unrecognized dysfunction in the BDNF/TrkB/CREB signaling pathway, driven by Aβ accumulation within the cardiac nervous system. This disruption highlights neurotrophic signaling as a promising and novel pharmacological target for combating AD‐induced cardiac dysfunction. While further clinical and translational studies are needed to validate these conclusions fully, our results strongly suggest that treating AD‐related heart failure (HF) may require a therapeutic strategy specifically targeting the cardiac neuronal system, which extends beyond conventional HF pharmacotherapy.

This emerging perspective calls for deeper investigation into the neuro‐cardiac axis in AD. Importantly, our study opens a new translational avenue that may help personalize AD therapy in subjects with cardiac pathology, as well as characterize AD as a multi‐organ neurodegenerative syndrome.

### 3.1 Limitations

While our study provides valuable insights into the cardiac implications of AD, several limitations should be acknowledged. Due to the frailty of our AD mouse model and the desire to minimize stress‐induced effects, we refrained from performing hemodynamic analysis, which could have provided further insights but may have impacted our animal population adversely [70]. Other neurotrophic factors, such as NT3 and NT4, may also contribute to cardiac innervation and function. Future investigations should explore the potential involvement of these factors in the AD myocardium to provide a comprehensive understanding of the cardiac neuro‐signaling dysregulation. The potential of the BDNF/TrkB/CREB signaling reactivation to reverse or prevent cardiac dysfunction remains to be tested in vivo. Studies employing pharmacological strategies to increase cerebral amyloid clearance are also under development [71], to determine if reducing brain amyloid pathology may ameliorate heart function. Additionally, generating AD mouse models with BDNF cardiomyocyte‐specific deletion could significantly enhance future research approaches. However, the combined mutations may lead to early morbidity and reduced lifespan [72, 73], therefore careful experimental design and substantial investment in resources and technology will be needed. Also, we acknowledge that in vivo validation of restored BDNF/TrkB signaling would strengthen the translational impact of our work. Pharmacologic and genetic interventions (LM22A‐4 or AAV‐BDNF) in an age‐dependent neurodegenerative model such as Tg2576, carry unresolved toxicological and dosing uncertainties that need evaluation and optimization, thus demanding extensive monitoring and resources. Although we postulate that TrkB stimulation will rescue cardiac BDNF signaling, thereby attenuating AD‐mediated cardiac maladaptive remodeling and improving cardiac function in Tg‐AD mice, the in vivo validation will require further research. Cross‐model validation would also strengthen generalizability. To our knowledge, this is the first report of cardiac alterations in Tg2576 mice [19]. Other AD models, including APP/PS1 and 3xTg‐AD, similarly require advanced age (>13 months) to manifest relevant phenotypes, further prolonging colony generation and introducing unpredictable delays to the overall study timeline. Yet, to expand the translational scope of our work, we are actively assessing cardiac outcomes in other AD models to support future cross‐model validation. In our study, we demonstrated that SAA3, a known inflammatory marker for ischemic heart disease [53, 54, 55], predominantly accumulates in the cardiac vessel walls of Tg2576‐AD mice, together with Aβ deposits. We also show that misfolded Aβ peptides or oligomers reach the heart. Aβ aggregates could potentially affect local misfolding‐related changes of other systemic amyloids around myocardial vessels. However, any role in seeding of systemic amyloid by Aβ in the heart remains speculative and is not supported by biochemical evidence in the current study, thus opening promising avenues for future studies. Differential trafficking may also underlie why Aβ40 is more likely to be recycled or deposited in vascular compartments, providing a plausible mechanism for accumulation in the heart. Indeed, at the blood‐brain barrier, Aβ40 preferentially accumulates in recycling endosomes, whereas Aβ42 is more sequestered in lysosomes [74]. These findings underscore the need for further investigation into the mechanistic causes of Alzheimer's heart pathology, an important avenue for future research. Lastly, the sample size of our human cardiac post‐mortem specimens is limited, as obtaining human cardiac samples from AD patients without significant cardiovascular diseases is extremely challenging. Future, more comprehensive clinical studies, including longitudinal studies in living subjects with AD or mild cognitive impairment (MCI), are recommended to confirm AD‐related cardiac dysfunction and validate the Aβ‐mediated myocardial denervation observed in our study. Despite these limitations, our findings offer novel key insights into the pathophysiology of AD‐related cardiac dysfunction, underscoring the need for further research to improve the clinical management of AD patients.

## 4 Conclusions

In summary, our study reveals that AD amyloid pathology can impact cardiac function, highlighting a maladaptive cardiac remodeling characterized by amyloid deposition, fibrosis, and a previously unknown dysregulation of the neurotrophic signaling pathway in AD models and human hearts. The results further suggest a potential mechanism whereby Aβ‐related decreases in cardiomyocyte CREB activation could reduce BDNF transcription, thereby contributing to myocardial denervation, pro‐apoptotic signaling, and physiological dysfunction. These findings significantly contribute to our understanding of AD pathophysiology, particularly highlighting the need to better understand cardiac dysfunction in AD. Moreover, our study identifies the neurotrophic signaling pathway as a potential therapeutic target within i the cardiovascular system in AD and related dementias. Finally, this study suggests that cardiac complications may warrant consideration in the broader context of AD management and highlights promising avenues for future translational investigation and therapeutic exploration.

## 5 Experimental Section

### 5.1 Animal Model

Male and female Tg2576 mice, a model of cerebral amyloidosis bearing the Swedish mutation (KM670/671NL), and age‐matched wild‐type (WT) littermates were bred in‐house. Mice were maintained under controlled conditions (∼22°C and in a 12‐h light‐dark cycle, lights from 7 am to 7 pm) with unrestricted access to food and water. The generation of B6; SJL‐Tg (APPSWE)2576Kha mice (Tg2576) on a B6; SJL Mixed Background was described in [75]. Tg2576 mice are known to develop numerous cerebral parenchymal amyloid‐β plaques at 11–13 months and CAA at 10‐11 months [76]. All experiments and animal protocols were performed in equal numbers of male and female mice, according to procedures approved by the Institutional Animal Care and Use Committee of Temple University School of Medicine and conformed to the National Research Council Guide for the Care and Use of Laboratory Animals published by the US National Institutes of Health (2011, eighth edition).

### 5.2 Assessment of Cardiac Function by Transthoracic Echocardiography

Transthoracic bi‐dimensional M‐mode echocardiographic analysis was accomplished in 4, 8, and 13 months‐old mice (before and after the development of brain Aβ pathology), under anesthesia, using a VisualSonics Vevo 2100 system (VisualSonics, Toronto, Canada). Before echocardiographic evaluation, the chest of the mice was carefully depilated by an operator. Subsequently, the mice were positioned in a clinostatic orientation on a temperature‐controlled surface, with ECG leads embedded for monitoring, ensuring a constant temperature of 37°C. Anesthesia was then administered to the animals via an anesthetic chamber, utilizing isoflurane (Zoetis IsoFlo, Kalamazoo, MI), with an induction concentration of 3.0% and a maintenance range of 1–2.5%. This anesthesia regimen was meticulously managed to keep the animals within the desired anesthesia depth throughout the procedure, optimizing their physiological stability. Cardiac function and structure were evaluated by measuring left ventricle (LV) wall thickness, end‐diastolic diameter (LVEDD), end‐systolic diameter (LVESD), end‐diastolic volume (LVEDV), end‐systolic volume (LVESV), ejection fraction (EF%), and fraction shortening (FS%) percentage [77]. In accordance with established protocols [78], speckle tracking‐based strain echocardiography (STE) was conducted using a parasternal long‐axis B‐mode loop captured through the VisualSonics Vevo 2100 system (VisualSonics). High‐speed imaging was ensured, with all recordings comprising 300 frames at a rate exceeding 200 frames per second, digitally stored as cine loops. Subsequently, the images obtained underwent comprehensive analysis using Vevo Strain Software (Vevo LAB 1.7.1). LV endocardial strain was computed in both radial and longitudinal axes and expressed as peak strain percentage. Additionally, LV deformation rate over time was evaluated and presented as strain rate (SR; peak 1/s). Global and regional assessments of LV endocardial longitudinal and radial strain, along with SR, were performed based on the average measurements derived from six LV segments (comprising basal, mid, and apical anterior segments, as well as basal, mid, and apical posterior segments). Regional LV endocardial longitudinal and radial strain, along with SR, were analyzed individually for each LV segment and then averaged across both the anterior and posterior walls. Vector diagrams illustrating the direction and magnitude of endocardial deformation were acquired from parasternal long‐axis B‐mode images and subjected to detailed analysis. Diastolic function was assessed using conventional echocardiography integrated with pulsed‐wave Doppler imaging. From the Doppler spectral waveforms, we quantified peak early‐ and late‐diastolic Mitral Valve transmitral velocities (E and A waves) to calculate the E/A ratio, along with Mitral Valve E‐wave deceleration time and isovolumetric relaxation time (IVRT). To further refine the evaluation of diastolic dysfunction, we applied the reverse‐peak analysis to derive reverse global longitudinal strain (GLS) and global radial strain (GRS) rates, which were derived from long‐axis B‐mode traces [78, 79], providing a more comprehensive characterization of myocardial relaxation [78, 79]. Lastly, animals were euthanized at 13 months for organ explant and biomolecular and histological analysis.

### 5.3 Tissue Processing and Morphological Evaluations

Cardiac specimens were fixed in Zamboni's solution (2% paraformaldehyde and picric acid) overnight at 4°C, then cryoprotected with 20% sucrose in PBS for 24 h at 4°C and cut in 5‐µm‐consecutive thick longitudinal sections using a cryotome (Leica 2000R, Germany). Interstitial fibrosis was assessed via Picrosirius Red Stain Kit (Abcam, ab150681), and images were acquired using a Nikon NiE Fluorescence Microscope with an 20X objective [80]. Cardiac fibrosis density was measured using automated threshold‐based algorithms through ImageJ software (NIH, version 1.30) and expressed as a percentage of fibrotic area normalized to the total LV area. A single operator blindly executed all the quantifications.

### 5.4 Immunostaining

The cardiac sections were processed via the indirect immunofluorescence method using rabbit polyclonal antibodies against tyrosine hydroxylase (TH, Millipore, AB152; 1:100) to stain adrenergic nerve fibers and growth association protein 43 (GAP‐43, Millipore, #AB5220; 1:100) for regenerating nerve terminals. Cardiac nerve fibers showed a regular pattern along cardiomyocytes [81]. Heart nerve ending density was quantified using automated threshold‐based algorithms through ImageJ software (NIH, version 1.30). Images were acquired using a Nikon Eclipse Ti fluorescence microscope with 20X and 60X objectives. Five areas were randomly chosen for quantification from each sample's three sections. Cardiac nerve fiber density was measured and expressed as a percentage of cardiac nerve fibers normalized to the total LV area. A single operator assessed all the measurements blindly. Next, to evaluate amyloid‐β accumulation, 5‐ µm‐thick cardiac sections from each experimental group were marked with 6E10 anti‐Aβ antibody (Covance, SIG‐39320; 1:500) and co‐stained with cardiac troponin T (cTnT, Thermo Scientific, #MA5‐12960; 1:250), collagen type I (Rockland Immunochemicals, #600‐401‐103‐0.1; 1:100), cardiac Troponin I (cTnI, Abcam, ab47003; 1:200), or cleaved caspase‐3 (Asp175) (Cl‐Casp‐3, Cell Signaling, #94530; 1:200) antibodies. Additionally, to characterize βsheet (fibrillar) amyloid deposits, cardiac sections were stained with Thioflavin‐S solution (Acros organics, 213150250; 0.5 g of Thioflavin‐S powder in 100 mL dH_2_O) for 30 min at room temperature shielding from the light. Next, the sections were dehydrated in 80% ethanol and thus washed in PBS 1X buffer. Nuclei were labeled with 4', 6‐diamidino‐2‐phenylindole (DAPI, Sigma–Aldrich, D9542; 1:5000), and sections were mounted using an aqueous mounting medium. Digital images were captured and analyzed by a Nikon Eclipse Ti fluorescence microscope with a 60X objective. To evaluate vascular components, after fixation, cardiac specimens were cut into 5‐µm‐thick longitudinal sections using a cryotome (Leica 2000R, Germany). Next, tissue sections were incubated for 30' min at 4°C in a blocking solution (10% donkey serum supplemented with 0.3% Triton X‐100 in phosphate‐buffered saline). Via indirect immunofluorescence procedure, sections were analyzed using goat polyclonal antibodies against CD31 (R&D Systems, AF3628‐; 1:30) and α‐smooth muscle actin (α‐SMA, Abcam, ab32575; 1:500) at 4°C overnight, followed by specific secondary antibodies conjugated with cyanine 3 or cyanin 5, respectively, to stain vascular network. For serum amyloid A3 immunostaining (SAA3), fixed 5‐µm‐thick cardiac sections were retrieved with formic acid 70% solution (Thermo Scientific, #270480010) for twenty minutes at room temperature, they were then stained using mouse monoclonal antibody against SAA (Abbexa, abx018140, 1:50) for 2 h at 37°C, followed by specific secondary antibodies combined with Alexa Fluor 488‐conjugated anti‐mouse IgG. DAPI was used to counterstain nuclei and cardiac sections were mounted with a Vectashield vibrance antifade mounting medium (H‐1000). To assess the changes in the cross‐sectional area (CSA) of cardiomyocytes, 5µm‐thick cardiac sections from each experimental group were incubated for 30 min at 4°C in a blocking solution (10% donkey serum) and permeabilized with 0.3% Triton X‐100 in phosphate‐buffered saline. The tissue sections were then stained with 5 µg/mL wheat germ agglutinin (WGA, #W11261, Thermo Fisher Scientific, Waltham, MA, USA) as previously described [65]. Afterward, the sections were mounted with Vectashield Vibrance antifade mounting medium (#H‐1000). Digital images were captured and analyzed using a Nikon Eclipse Ti fluorescence microscope with a 20X objective. Five to six randomly selected fields per sample were analyzed, totaling 50‐60 cardiomyocytes per section (10 cells per area). The mean cardiomyocyte diameter (µm) was measured using the ImageJ software. Lastly, to assess amyloid‐β deposits in the human heart, 5‐ µm‐thick LV sections from healthy donors and AD with CAA patients were stained with 6E10 anti‐Aβ antibody (Covance, SIG‐39320; 1:500) and co‐stained with cardiac Troponin I (cTnI, Abcam, ab47003; 1:200). Images were acquired and examined using a Nikon Eclipse Ti fluorescence microscope with 20X and 60X objectives. A single operator blindly performed all the procedures.

### 5.5 Preparation of Cell and Tissue Lysates and Immunoblotting Analysis

Cellular and cardiac samples were homogenized using RIPA cell lysis buffer (Thermo Scientific) enriched with phosphatases and protease inhibitors (Thermo Scientific). Then, the lysates underwent sonication and were centrifuged for 10 min at 4°C at 13,000 rpm to discard the insoluble debris. Next, total protein amounts were quantified via a dye‐binding Pierce BCA protein assay kit (Thermo Scientific) and detected using a spectrophotometer reader (SpectraMax i3x Multi‐mode Microplate Reader, Molecular Devices) at a wavelength of 562 nm. Equal yields of protein (20–40 µg) were separated through SDS‐PAGE and identified by western blot (WB) analysis. Total lysates were used to evaluate the protein levels of NGF (Alomone labs, AN‐240; 1:1000), BDNF (Alomone labs, ANT‐010; 1:1000), GAP‐43 (Millipore, #AB552; 1:1000), Cleaved Caspase‐3‐ Asp175 (Cl‐Casp‐3, Cell Signaling, #94530; 1:500), dopamine β hydroxylase (DβH, Millipore, #AB1536; 1:1000), human Aβ [mouse monoclonal anti‐Aβ antibodies mixture composed of 4G8 epitope (residues Aβ18‐22, Covance, SIG‐39320; 1:1000) and 6E10 epitope (residues Aβ3‐8, Covance, SIG‐39220; 1:1000)], serum amyloid A3 (SAA3, Abcam, #ab231680; 1:500), TrkB [p Tyr816] (Novus Bio, #NBP1‐03499SS; 1:1000), TrkB (80E3) (Cell Signaling, #4603; 1:1000), phospho‐CREB (Ser133) (1B6) (Cell Signaling, #9196; 1:1000), CREB (Cell Signaling, #9197; 1:1000), p75NTR (Sigma Aldrich, 07‐476; 1:1000), fibronectin (Abcam, #ab2413; 1:1000), matrix metalloproteinases‐ 2 (MMP‐2, Cell Signaling, #35814; 1:1000), Laminin A/C (Santa Cruz Biotechnology, sc‐376248; 1:500), Actin (Millipore, MAB1501; 1:1000) and GAPDH (Santa Cruz Biotechnology, sc‐32233,6C5; 1: 2000), the three latter of which were used as loading controls. Protein bands were detected using Odyssey CLx Imaging System according to the manufacturer's instructions and quantified with Image Studio Lite Software [82].

### 5.6 ELISA

Aβ40 and Aβ42 peptide levels were detected using a commercial ELISA kit (Invitrogen KHB3481 and KHB3441, respectively). Briefly, cardiac specimens for each mouse were collected at the end of the experimental study and homogenized with RIPA cell lysis buffer (Thermo Scientific), supplemented with phosphatases and protease inhibitors (Thermo Scientific, Waltham, MA). Then, ELISA was performed using an equal amount of total cardiac protein following the company's specifications, and the plate was evaluated at 450 nm with a spectrophotometer reader (SpectraMax i3x Multi‐mode Microplate Reader, Molecular Devices).

Cardiac mature BDNF, mature NGF, and their pro‐neurotrophin isoforms (proBDNF and proNGF) were quantified using commercial ELISA kits (biosensis BEK‐2211/2237 and BEK‐2213/2236). Cardiac tissue from each mouse was collected at study completion, homogenized in RIPA buffer (Thermo Scientific) supplemented with phosphatase and protease inhibitors, and processed according to the manufacturer's protocol. Equal amounts of total cardiac protein were loaded for ELISA, and absorbance was measured at 450 nm using a SpectraMax i3x multimode microplate reader (Molecular Devices).

### 5.7 Aβ peptides

Pre‐aggregated Aβ40 and Aβ42 oligomers were generated from HFIP‐pretreated and lyophilized peptides as previously described [83]. Briefly, oligomer enriched Aβ40 and Aβ42 preparations were obtained after a dilution of the lyophilized peptide in DMSO at a concentration of 5 mM, then diluted to 100 µM in ice‐cold DMEM/F12 media and incubated at 4°C for 16 h. Next, Aβ40 and Aβ42 oligomers were resuspended in DMEM/F12 medium to 10 µM as a final concentration for the cell treatments.

### 5.8 Cell culture and treatments

Human ventricular myocardial cardiomyocytes (AC16, purchased from Millipore, #SCC109; RRID: CVCL_4U18) were expanded in 10 cm^2^ dishes not further than the 10th passage, in a humidified environment at 37°C with 5% CO_2_ in DMEM/F12 medium (Lonza Ltd. Basel, Switzerland) supplemented with 12.5% fetal bovine serum (FBS) and 1% penicillin‐streptomycin (P/S, Lonza). AC16 cells were grown in the pre‐contractile developmental stage, thus allowing us to better track molecular events and cellular responses [84, 85], without the complications and variability associated with isolated adult cardiomyocyte cultures. Human SH‐SY5Y neuroblastoma cells (ATCC, CRL‐2266; RRID: #CVCL_0019, a neuronal cell line used widely in experimental neurological studies [86, 87, 88]) were grown in 10 cm^2^ dishes within the 10th passage in a humidified environment at 37°C with 5% CO_2_ in DMEM/F12 medium (Lonza Ltd. Basel, Switzerland) supplemented with 10% fetal bovine serum (FBS) and 1% penicillin‐streptomycin (P/S, Lonza). For both cell types, AC16 and SH‐SY5Y, an equal number of cells were seeded in 6‐well plates. After 24 h, when the cells reached approximately 70–80% confluency, they were challenged with human Aβ40 or Aβ42 oligomers (10 µM) for 16 h. In some studies, cells were exposed to human Aβ40 oligomers (10 µM) for either 15 min or 1 h or treated with Scrambled‐Aβ (1‐40) for 16 h, purchased from ANASPEC (AS‐24625), as a control. For other experiments, cells were exposed to the BDNF/TrkB receptor agonist LM22A‐4 (100 nM) or the antagonist ANA‐12 (30 µM) for 16 h. LM22A‐4 (4607) and ANA‐12 (SML0209) were obtained from Tocris Bioscience and Sigma Aldrich. All cells used in the in vitro experiments were free of contamination.

### 5.9 Maintenance and Treatment of Human‐Induced Pluripotent Stem Cell‐Derived Cardiomyocytes (hiPSC‐CMs)

A commercially available hiPSC‐CM line, iCell Cardiomyocytes^2^ (Catalog number R1220, Kit/lot 12,012; FUJIFILM Irvine Scientific, Inc., United States; RRID: SCR_016538), was utilized for this study [89]. The cells were seeded into a 0.1% gelatin‐coated 12‐well plate at a density optimized to form a monolayer (≈210K cells per well) and cultured in a stage incubator (37°C, 5% CO2) following the provider's guidelines. Experiments were conducted 5–7 days post‐plating, ensuring the cells formed a viable, beating monolayer, in accordance with the manufacturer's instructions (Video S1). Cells from this specific lot were from the same donor (healthy, female, Caucasian, age <18), as verified by FUJIFILM Cellular Dynamics. For the experiment, hiPSC‐CMs were treated with human Aβ40 oligomers (10 µM) for 20 h, after which the cells were collected for biochemical analysis. For imaging, cells were fixed with 4% paraformaldehyde (PFA) for 10 min, washed with 1X PBS, and then blocked and permeabilized for 30 min using PBS containing 0.1% Triton X‐100 and 2% normal donkey serum. hiPSC‐CMs were subsequently labeled with a BDNF antibody (Abcam, ab226843; 1:250) and co‐stained for cardiac troponin T (cTnT, Thermo Scientific, #MA5‐12960; 1:250), with nuclei labeled using DAPI (Sigma Aldrich, D9542; 1:5000). Imaging was conducted using a Nikon Eclipse Ti fluorescence microscope with a 100X objective, with all procedures performed by a single operator in a blinded manner. All the cells used for the in vitro experiments were contamination‐free.

### 5.10 Nuclear Protein Isolation

AC16 human cardiomyocytes were cultured in 6 well plates until they reached confluence and treated with human Aβ40 oligomers for 15 min. To extract the nuclear fractions, cells were collected using buffer A, containing 10 mmol/L HEPES at pH 7.9, 10 mmol/L KCl, 0.1 mmol/L EDTA, integrated with 25% NP‐40X‐100 non‐ionic surfactant detergent solution, enriched with protease inhibitors. The cell lysate was transferred in a 1.5 mL tube, placed in ice, and centrifuged for 10 min at 4°C at 13,000 rpm to discard the insoluble debris. The supernatant was transferred to a new 1.5 mL tube to preserve the cytosolic fraction, while the pellet was resuspended in a fresh prepared buffer B composed of 20 mmol/L HEPES at pH 7.9; 0.4 mol/L NaCl; 1 mmol/L EDTA; 10% glycerol; supplemented with protease inhibitor. Next, the tube containing the nuclei pellet dissolved in buffer B was placed on a rotator for 2 h at 4°C and then centrifuged at 13,000 rpm for 5 min at 4°C. Lastly, the supernatant (nuclear fraction) was transferred to a new 1.5 mL tube and stored at −80°C, ready for the following protein quantification and immunoblot analysis [82].

### 5.11 Human LV specimens

Frozen human LV post‐mortem tissue from healthy donors and patients with clinical diagnoses of AD and CAA were obtained from the Banner Sun Health Research Institute clinical biobank to investigate the impact of AD pathology on myocardial innervation. The selection criteria of the study population excluded individuals with comorbidities known to substantially affect cardiac function, including high blood pressure, cerebrovascular accidents (e.g., strokes and lacunes), brain/cardiac neoplastic conditions, secondary amyloidosis, prior myocardial infarction, and endocarditis. Healthy volunteers and AD+CAA patients were matched based on age, sex, and ethnicity. Human cardiac specimens were used for biochemical and histological characterization.

### 5.12 Statistical Methods and Analysis

Data were expressed as means±SEM. Distributions of all parameters were examined using histograms. Outliers were identified and excluded from the original dataset using the ROUT (*Q* = 1%) method with GraphPad Prism 9.1.0 software. For experiments with a single parameter, two‐sample Student's t‐tests were used to determine statistical significance between the two groups. For experiments with multiple parameters, two‐sample Student'st‐tests with Holm‐Šidák multiple comparison adjustment were used to determine statistical significance between groups. For experiments with multiple groups, one‐way or two‐way analysis of variance (ANOVA) with Tukey's multiple comparison test was used to determine statistical significance between groups. Differences between groups were considered statistically significant when p≤0.05. All data were analyzed and graphically represented using GraphPad Prism software version 9 (GraphPad, La Jolla, CA).

## Author Contributions

AE and SF designed the study. AE performed and conceptualized the experiments and analyzed the data. AE wrote the paper draft and did the literature search. RPR, AC, and RVT performed experiments. SF provided relevant insights, scientific supervision, critically revised the manuscript and acquired funding. HZ advised in the statistical analysis and edited the manuscript. SJ revised the study, edited the manuscript, and offered additional literature searches.

## Funding

This work was supported by NIH R01NS104127 and R01AG062572 grants, the Alzheimer's Association (AARG‐20‐685663), and the Pennsylvania Department of Health Collaborative Research on Alzheimer's Disease (PA Cure) Grant awarded to SF, by the NIH SC1GM128210 grant awarded to SJ, by American Heart Association Postdoctoral fellowship 24POST1240115 awarded to AE, by the Karen Toffler Charitable Trust, and the Lemole Center for Integrated Lymphatics research.

## Conflicts of Interest

The authors declare no conflicts of interest.

## Supporting information




**Supporting File 1**: advs74141‐sup‐0001‐SuppMat.docx.

**Supporting File 2**: advs74141‐sup‐0002‐Uncropped WB file.pdf.

**Supporting File 3**: advs74141‐sup‐0003‐Video.mp4.

## Data Availability

The data that support the findings of this study are available from the corresponding author upon reasonable request.
