# Supplementary material for: Amyloid β Instigates Cardiac Neurotrophic Signaling Impairment, Driving Alzheimer's Associated Heart Disease
Source: Adv Sci (Weinh). 2026 Feb 10;13(20):e11924. doi: 10.1002/advs.202511924 (PMC13067842; doi:10.1002/advs.202511924)
Supplement: Supplementary file 1 — Supporting File 1: advs74141‐sup‐0001‐SuppMat.docx. [file ADVS-13-e11924-s001.docx]

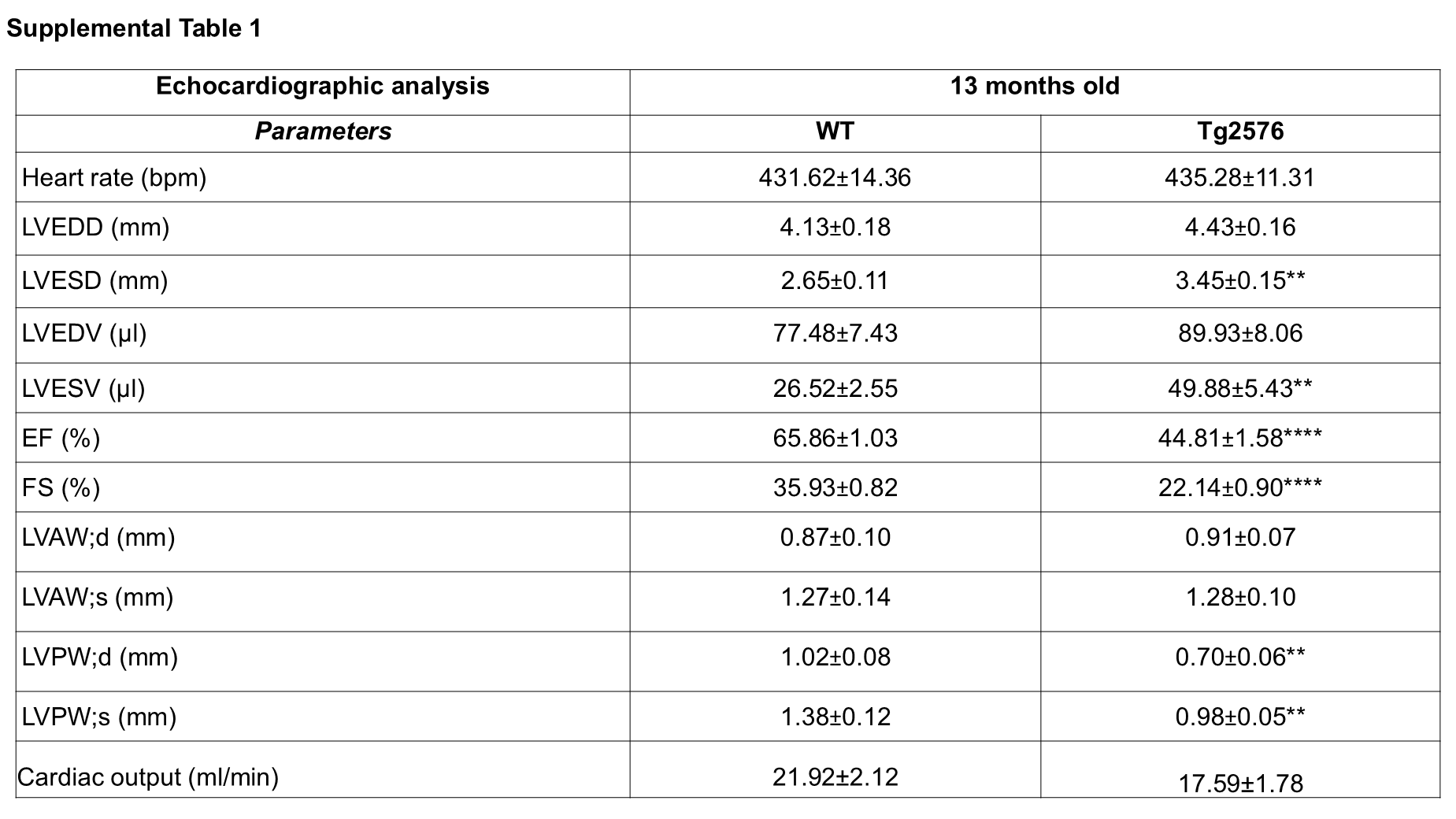


**Supplemental Table 1. Echocardiographic evaluation.** Conventional echocardiographic parameters were measured in 13-month-old WT and Tg2576 mice: heart rate, left ventricular (LV) end diastolic diameter (LVEDD), end systolic diameter (LVESD), end-diastolic volume (LVEDV), end systolic volume (LVESV), ejection fraction (EF), fractional shortening (FS), anterior wall in diastole (LVAW;d) and systole (LVAW;s), posterior wall in diastole (LVPW;d) and systole (LVPW;s), and cardiac output. (n=6 vs. n=6). Data are presented as a mean±SEM. *P<0.05, ^**^p<0.01, and ***p<0.001 vs WT. Student t-tests were performed between the groups.


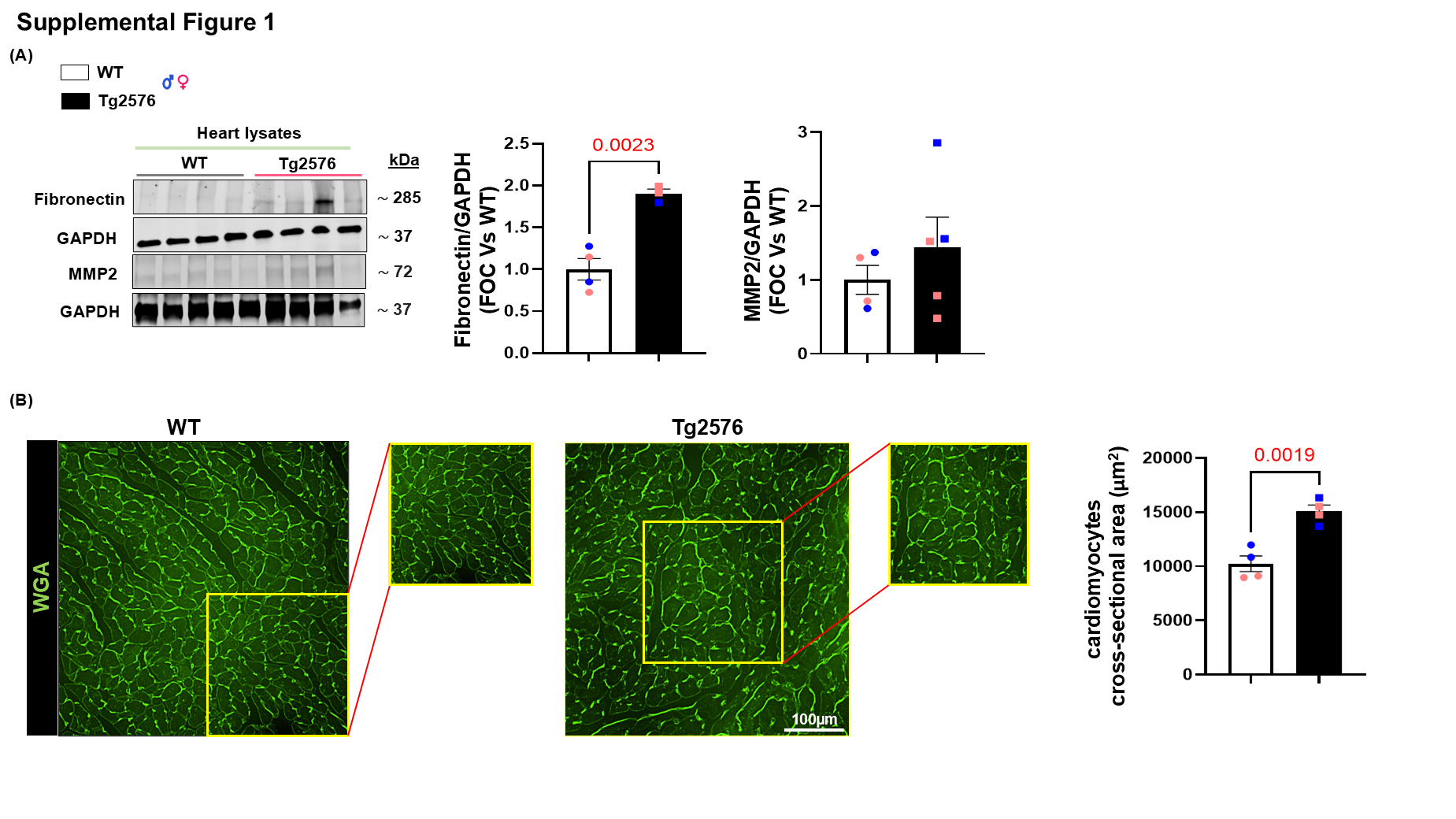
**Supplemental Figure 1. Cardiac adverse remodeling in 13-month-old Tg2576-AD mouse heart.** (**A**) Representative immunoblots (left panels) and densitometric quantitative analysis (right panels) showing protein levels of Fibronectin (n=4 vs. n=3, Student’s t-test P value 0.0023; Holm Sidak adjusted P value 0.0046), and MMP2 (n=4 vs. n=5, Student’s t-test P value 0.4058), in total cardiac lysates from WT and Tg2576 mice. GAPDH levels were used as a loading control. (**B**) Representative digital images (left panels, scale bar 100μm), and quantification (right panel) of the cardiomyocyte cross-sectional area (CSA) in cardiac sections from WT and Tg2576 mice stained with WGA. (n=4 vs. n=4, Student’s t-test P value 0.0019). Data are presented as a mean±SEM.


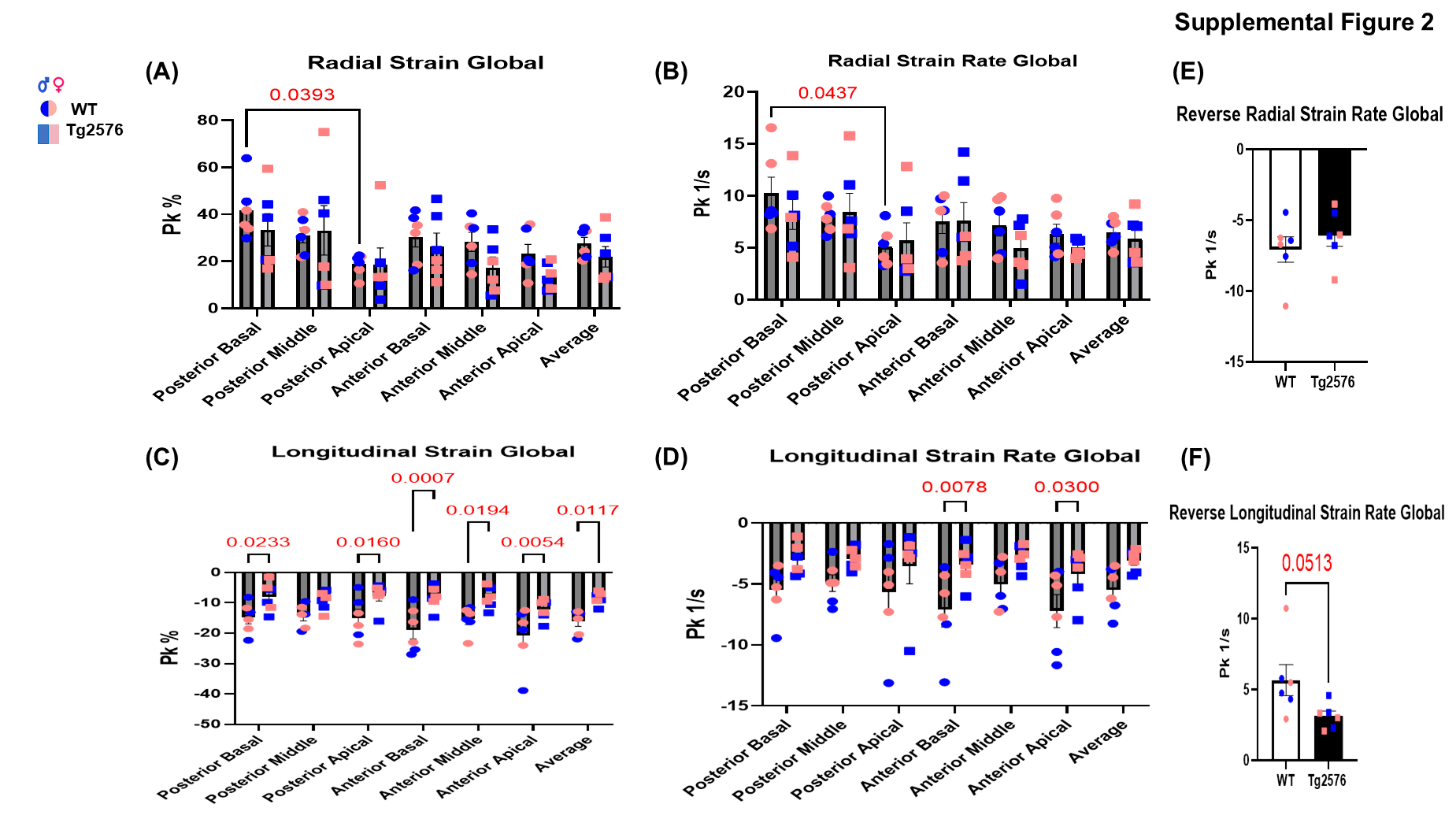
**Supplemental Figure 2. Longitudinal strain and strain rate LV contractility are impaired in Tg2576 AD mice.** Radial and longitudinal strain or strain rate measured at six LV segments as well as their average (**A**-**D**), and reverse (diastolic) radial (**E**) and longitudinal (**F**) SR global in 13 months old WT and Tg2576 mice were evaluated using speckle-tracking-based strain echocardiography. (n=6 vs. n=6). Data are presented as a mean±SEM. *P<0.05 and vs WT. Two-way ANOVA with Tukey’s multiple comparisons post hoc test (**A**-**D**), and student t-tests (**E**-**F**) have been performed between the groups.


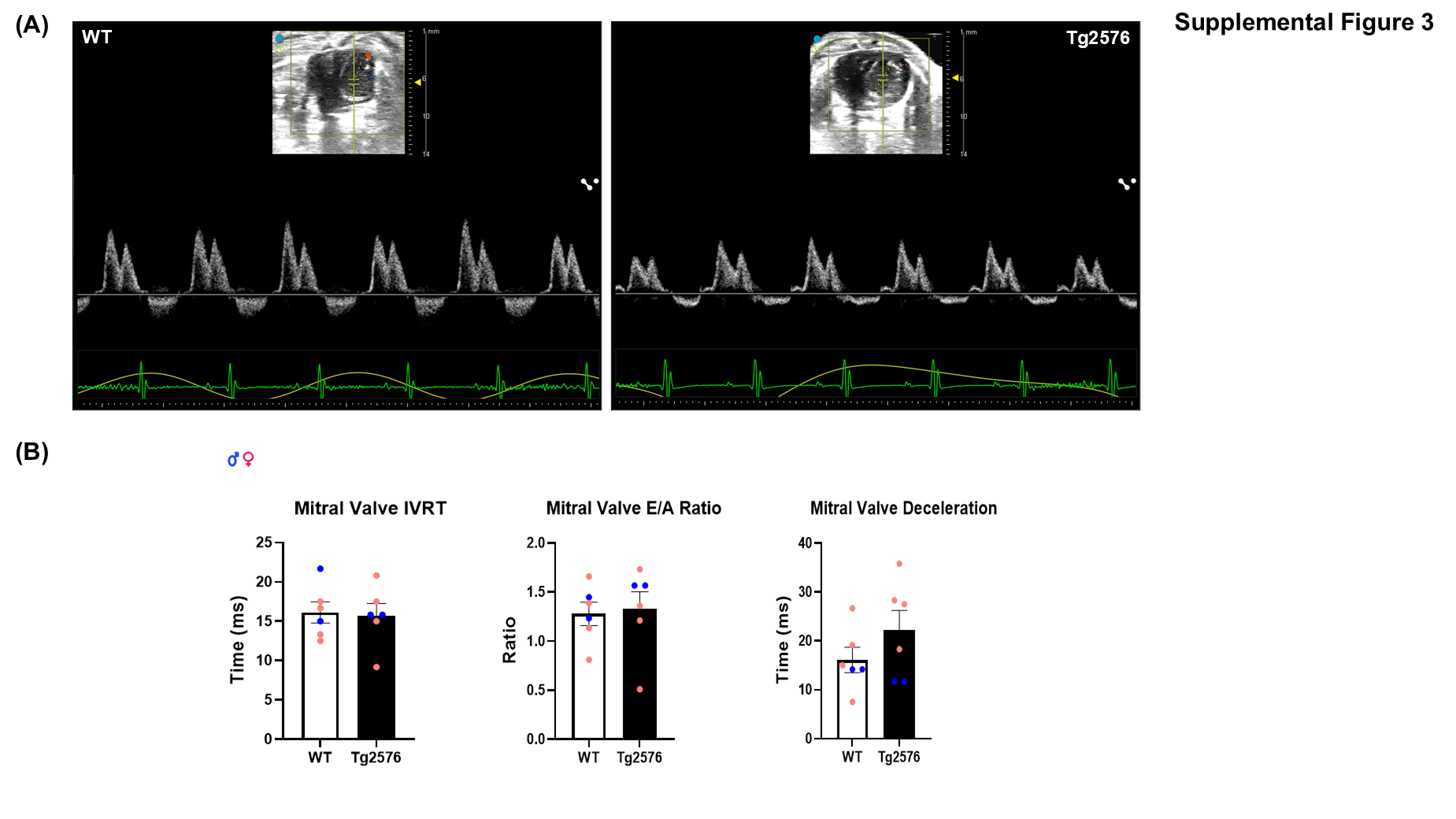


**Supplemental Figure 3. Evaluation of left ventricular diastolic function in 13-month-old mouse cohorts.** Conventional echocardiographic measurements were analyzed in 13-month-old male and female WT and Tg2576 mice to assess diastolic function. (**A**) Representative echocardiographic images of pulsed-wave Doppler of the Mitral Valve. (**B**) Quantification of the diastolic function parameters: Mitral Valve isovolumetric relaxation time (IVRT), Mitral Valve E/A ratio, and Mitral Valve deceleration time. (n=6 vs. n=6). Data are presented as mean ± SEM. *P<0.05 compared to WT. Student t-tests were performed between groups.


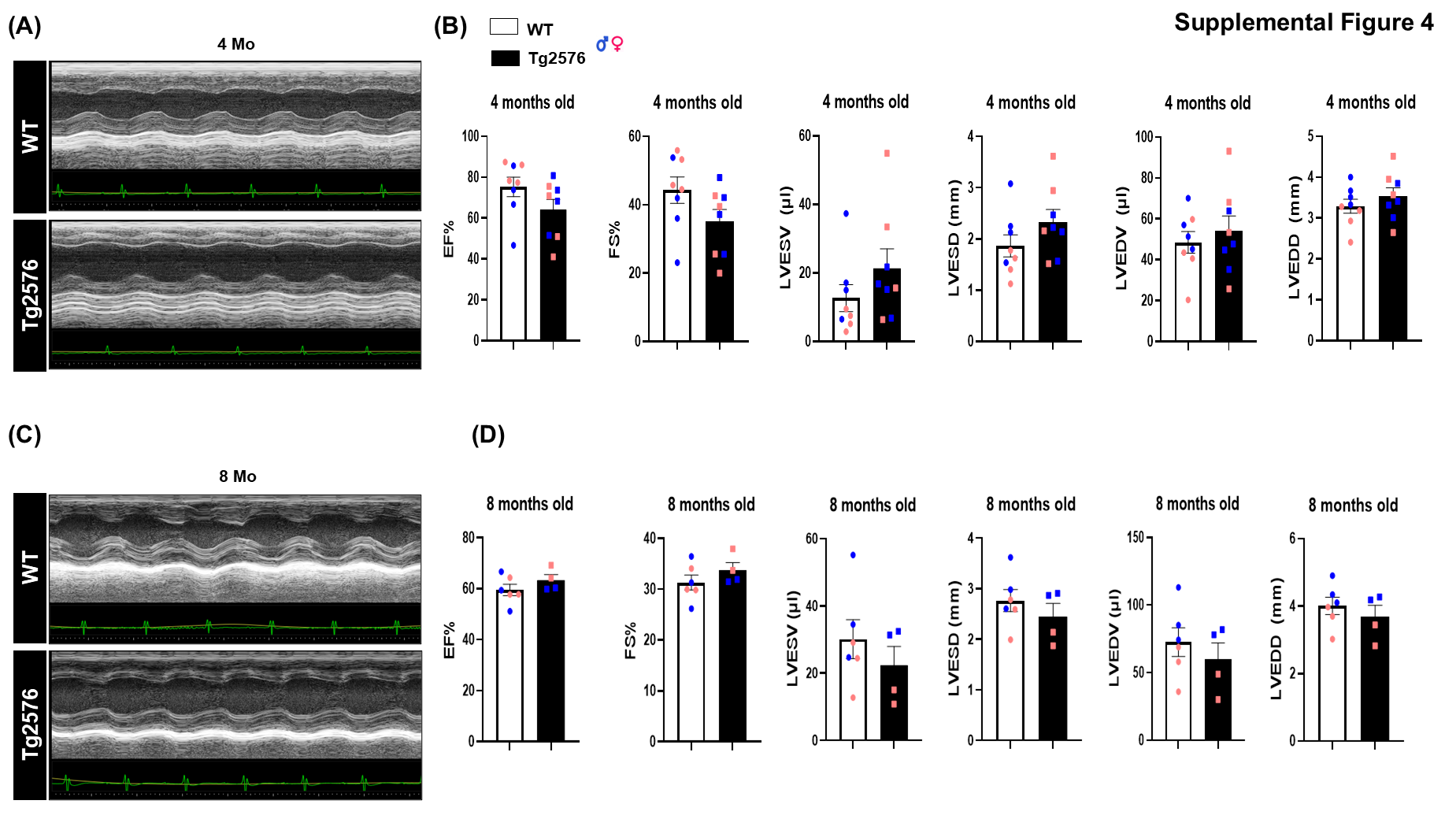
**Supplemental Figure 4. Cardiac function is not affected in 4 or 8 months old Tg2576 mice.** (**A**-**C**) Representative M-mode echocardiography images of WT and Tg2576 mice show no difference between the experimental groups. (**B**) Ejection fraction (EF%) and fractional shortening (FS%) percentage, left ventricular end-systolic and diastolic diameters (LVESD, LVEDD), and left ventricular end-systolic and diastolic volumes (LVESV, LVEDV) of age-matched (4-month-old) Tg2576 mice and WT littermates. (n=8 vs. n=8). (**D**) The same echocardiographic parameters were evaluated in 8-month-old animals from both experimental groups. (n=6 vs. n=4) Data are presented as a mean±SEM. *P<0.05 and vs WT. Student t-tests have been performed between the groups.


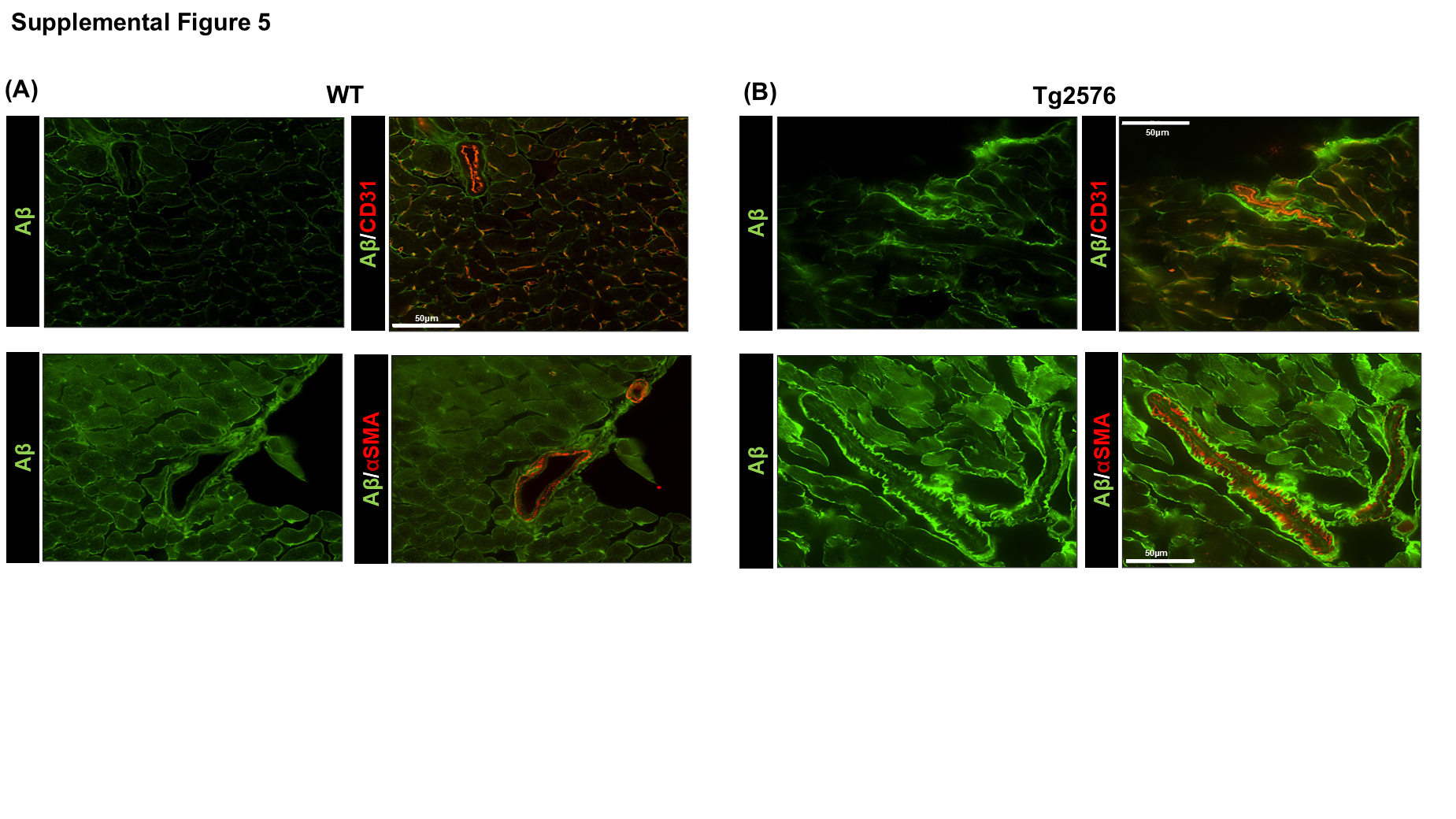
**Supplemental Figure 5. Amyloid-β surrounds cardiac vessels and infiltrates the vascular basal lamina.** (**A**-**B**) Representative digital images (upper panels, scale bar 50μm) showing vessels (endothelial cells stained with CD31, in red) and Aβ (in green), and representative digital images (lower panels; scale bar 50μm) showing α-smooth muscle actin (αSMA, staining vascular smooth muscle cells, in red) and Aβ (in green) in cardiac sections from 13 months old WT and Tg2576 mice.


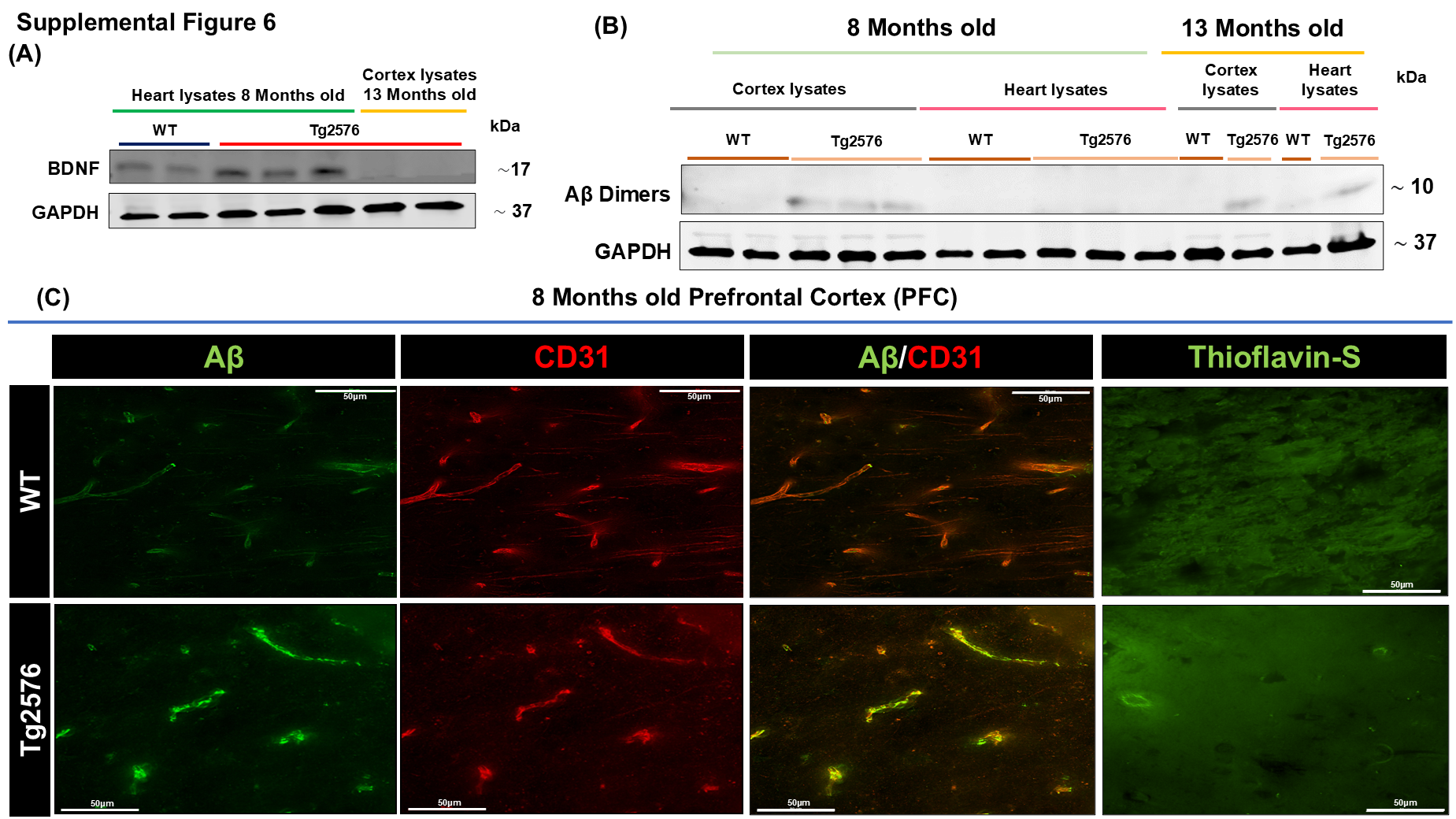


**Supplemental Figure 6. Amyloid-β deposits in vessels of 8-month-old Tg2576 prefrontal cortex (PFC), but not in heart tissue, with preserved cardiac BDNF expression.** (**A**-**B**) Representative immunoblots show BDNF (**A**) and Aβ dimers (**B**) levels in total cardiac lysates from WT and Tg2576 mice. A 13-month-old Tg2576 cerebral cortex lysate was used as an internal positive control. GAPDH levels were used as a loading control. (**C**) Representative digital images (scale bar 50μm) showing vessels (stained with CD31, in red) and Aβ deposits (in green). Right panels: representative digital images (right panels; scale bar 50μm) showing β- sheet conformation (fibrillar) amyloid marked with Thioflavin-S (in green) in the prefrontal cortex (PFC), in 8-months old WT and Tg2576 mice.


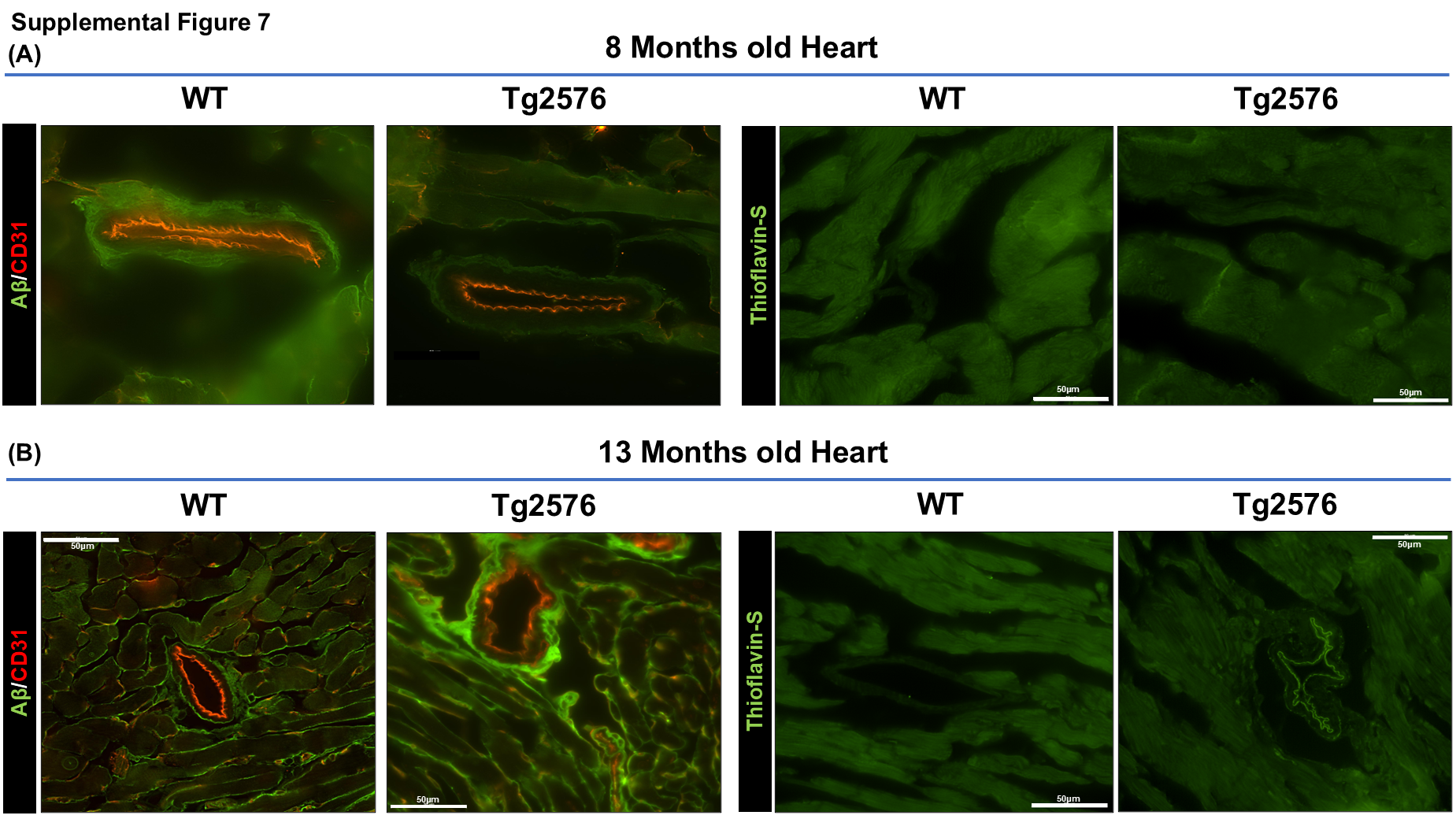


**Supplemental Figure 7. AD pathology affects the cardiac vascular network in 13-month-old Tg2576 mice**. (**A**-**B**) Representative digital images (left panels, scale bar 50μm) showing vessels (stained with CD31, in red) and Aβ deposits (in green), and representative digital images (right panels; scale bar 50μm) showing β-sheet conformation of amyloid marked with Thioflavin-S (in green) in cardiac sections from WT and Tg2576 mice.


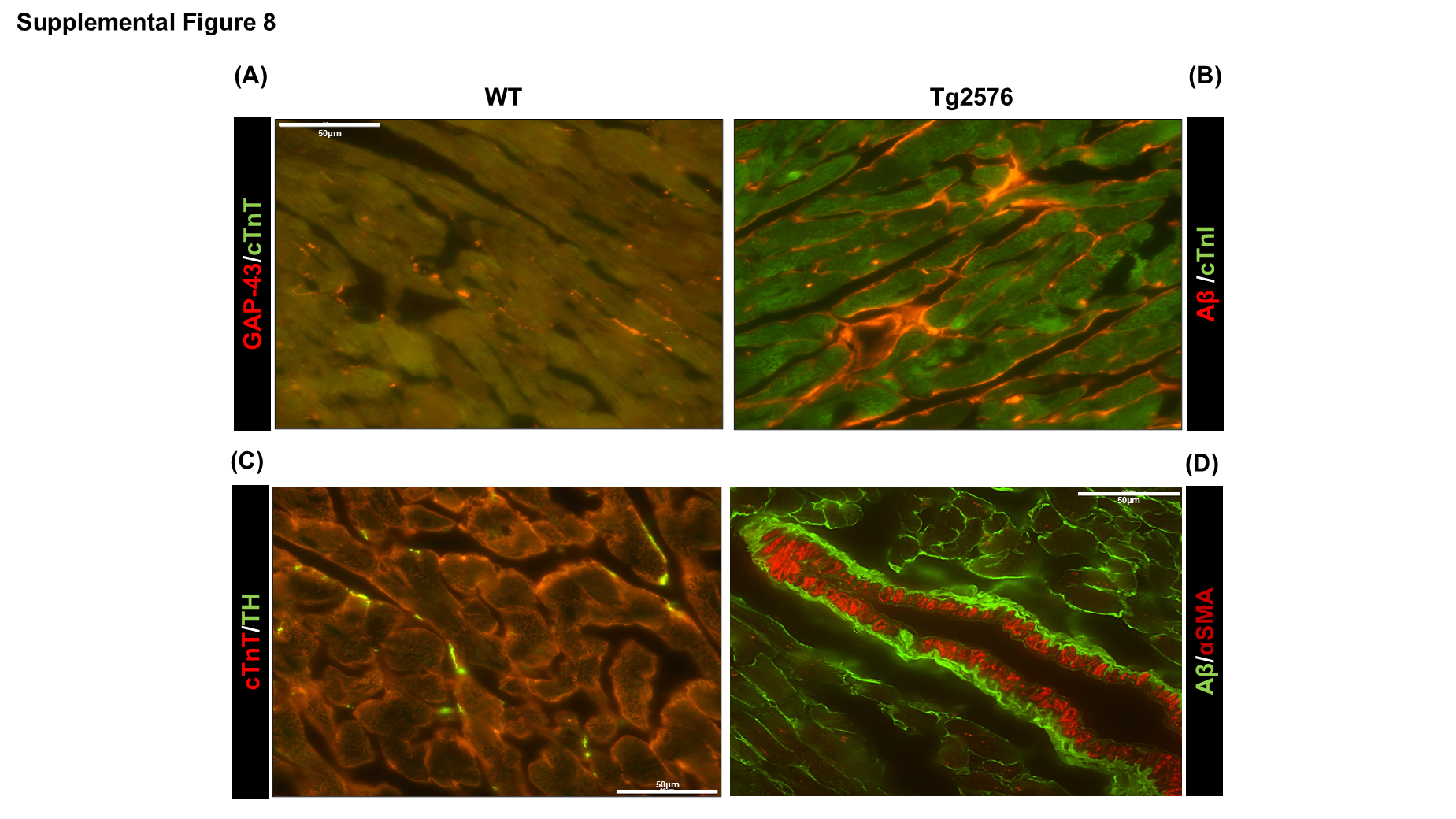


**Supplemental Figure 8.** Representative digital images (scale bar 50μm) of cardiac sections from 13-month-old WT and Tg2576 mice stained with (**A**) anti-neuronal regeneration marker (GAP-43, in red) and cardiac Troponin T (cTnT, in green). (**B**) Aβ (in red) and cardiac Troponin I (cTnI, in green). (**C**) anti-tyrosine-hydroxylase (TH, in green) and cardiac Troponin T (cTnT, in red). (**D**) Aβ (in green) and α-smooth muscle actin (α-SMA, in red).


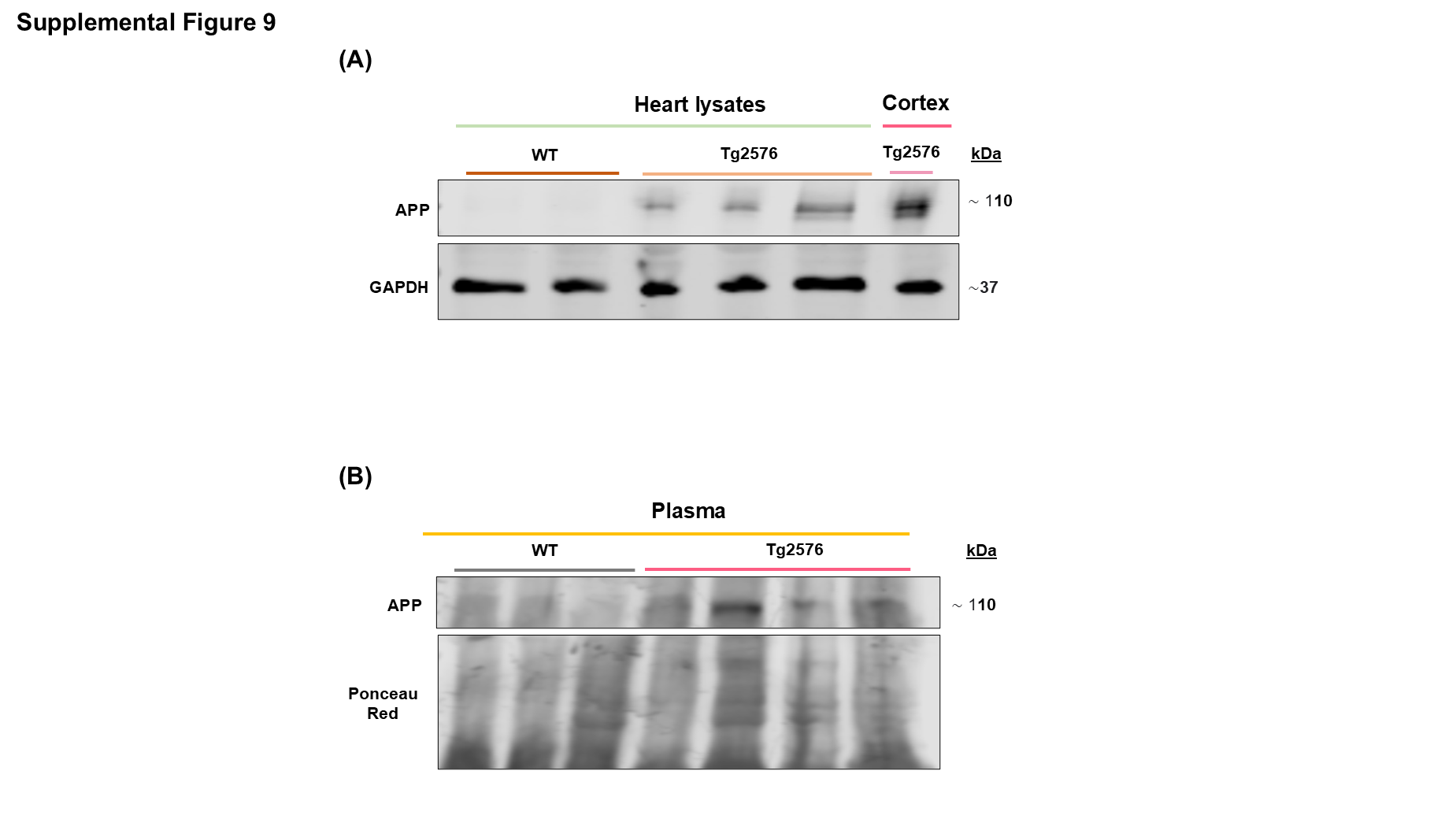


**Supplemental Figure 9. The Amyloid precursor protein (APP) is present in plasma and heart tissue of the 13-month-old Tg2576-AD model.** (**A**-**B**) Representative immunoblots showing protein levels of APP in total cardiac lysates (upper panel) and plasma APP levels (lower panel) in 13-month-old WT and Tg2576 mice. Tg2576 cortex lysate was used as an internal positive control. GAPDH levels or ponceau red were used as respective loading controls.


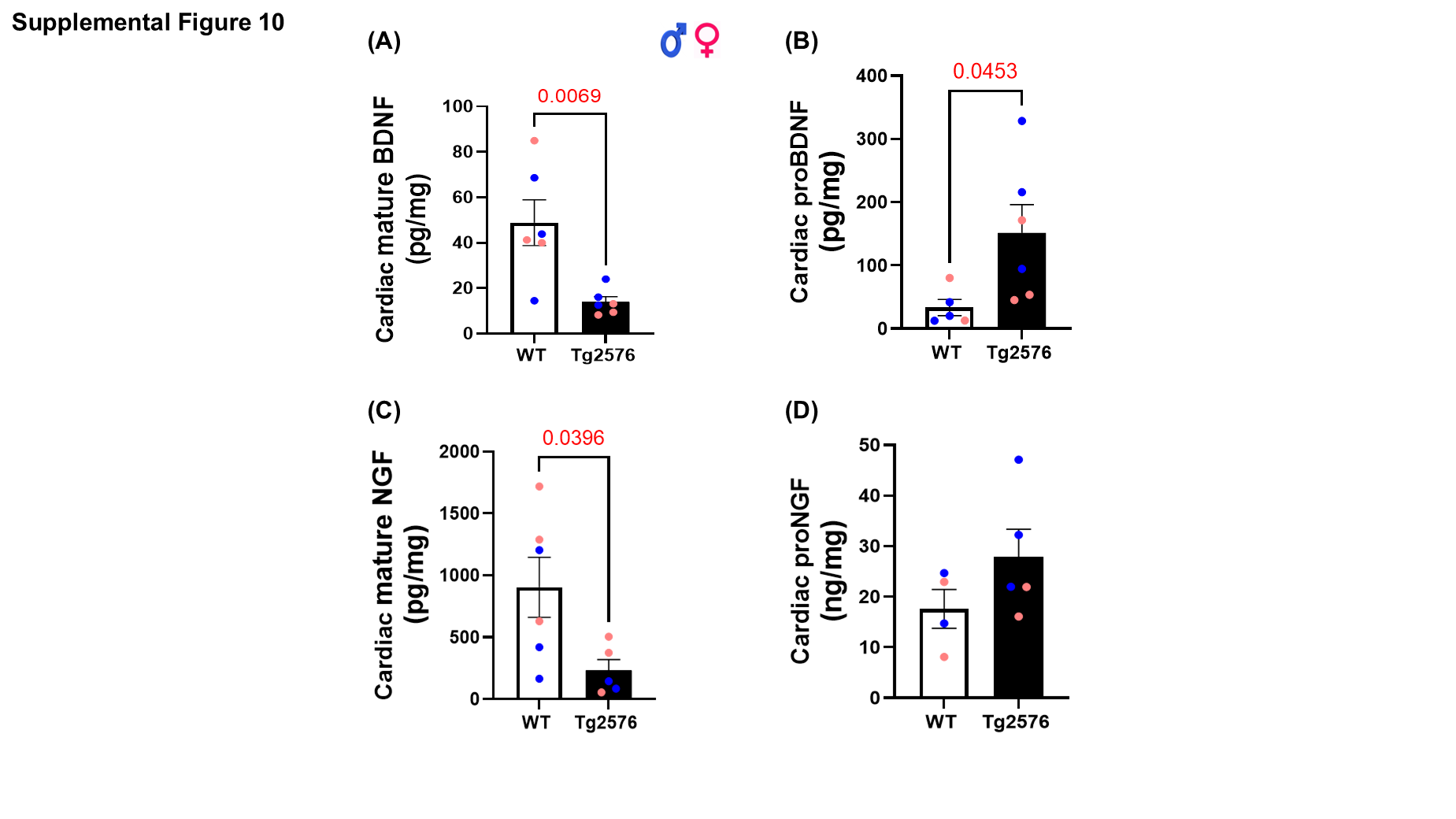


**Supplemental Figure 10. Impact of Alzheimer’s disease on cardiac neurotrophins profile.** (**A**-**D**): mature BDNF cardiac levels (pg/mg of proteins) (**A**) (n=6 vs. n=6). Student’s t-test P value 0.0069, proBDNF cardiac levels (pg/mg of proteins) (**B**) (n=5 vs. n=6). Student’s t-test P value 0.0453, mature NGF cardiac levels (pg/mg of proteins) (**C**) (n=6 vs. n=5). Student’s t-test P value 0.0396, and proNGF cardiac levels (ng/mg of proteins) (**D**) (n=4 vs. n=5). Student’s t-test P value 0.1889, assessed by ELISA assays, in total cardiac lysates from WT and Tg2576 mice. Data are presented as a mean±SEM.


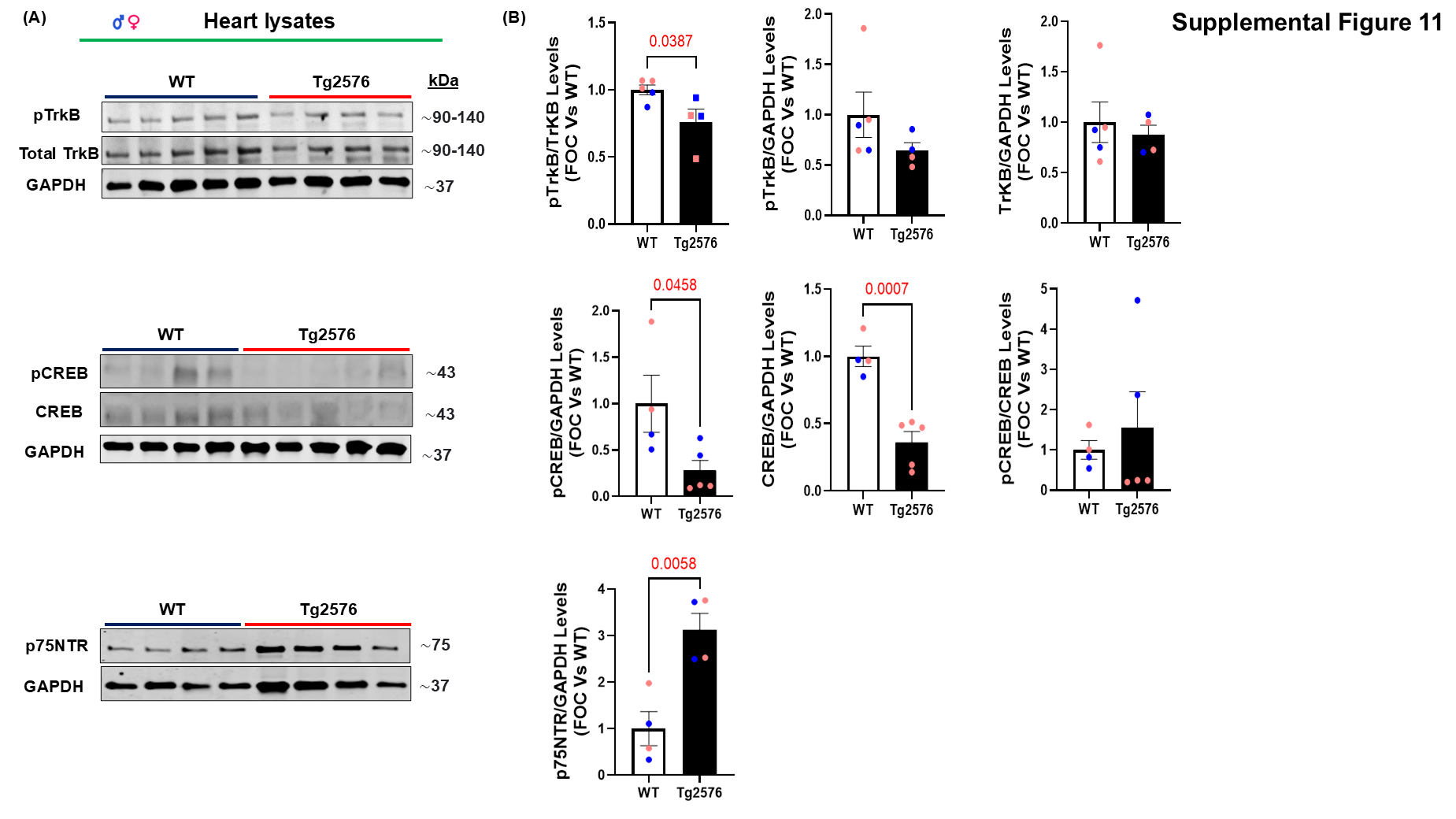
**Supplemental Figure 11. Alzheimer’s amyloid pathology impairs cardiac neuro-signaling pathway in Tg2576 mice.** (**A**-**B**): Representative immunoblots (**A**) and densitometric analysis (**B**) showing levels of pTrkB (n=5 vs. n=4, Student’s t-test P value 0.0387; Holm Sidak adjusted P value 0.1117), TrkB, pCREB (n=4 vs. n=5, Student’s t-test P value 0.0458; Holm Sidak adjusted P value 0.0895), CREB (n=4 vs. n=5, Student’s t-test P value 0.0007; Holm Sidak adjusted P value 0.0021), and p75NTR (n=4 vs. n=4, Student’s t-test P value 0.0058), in total heart lysates from WT and Tg2576 mice. GAPDH levels were used as a loading control. Data are presented as a mean±SEM.


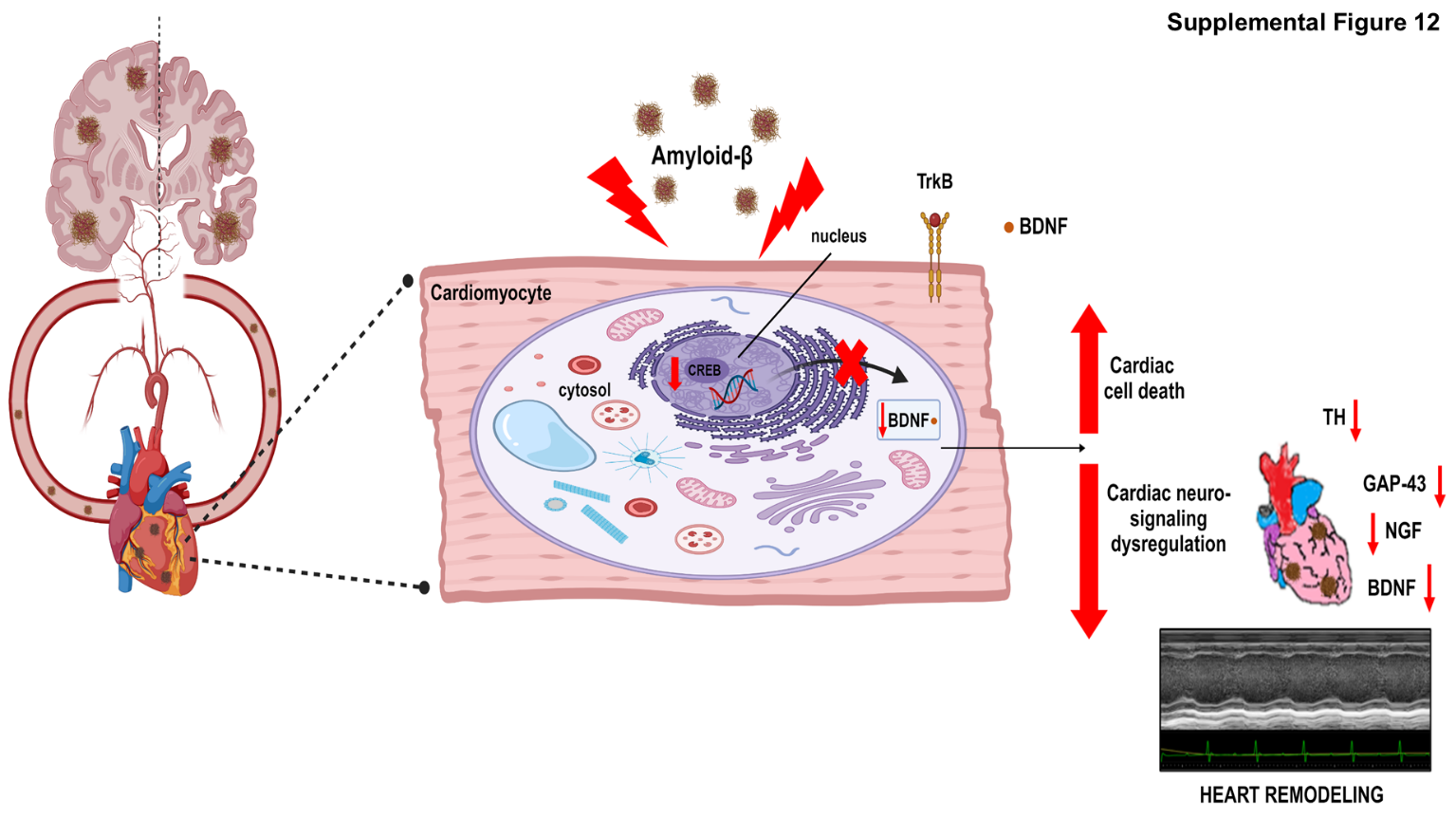
**Supplemental Figure 12. Conceptual scheme of the study**. Amyloid- β aggregates, present in the heart parenchyma of AD mouse models and AD human hearts, induce adverse cardiac remodeling and myocardial denervation through cardiac TrkB/CREB/BDNF neurotrophic signaling axis dysregulation, ultimately resulting in impaired cardiac physiology.
