# Supplementary material for: Amyloid β Instigates Cardiac Neurotrophic Signaling Impairment, Driving Alzheimer's Associated Heart Disease
Source: Adv Sci (Weinh). 2026 Feb 10;13(20):e11924. doi: 10.1002/advs.202511924 (PMC13067842; doi:10.1002/advs.202511924)
Supplement: Supplementary file 2 — Supporting File 2: advs74141‐sup‐0002‐Uncropped WB file.pdf. [file ADVS-13-e11924-s002.pdf]

Figure 1H

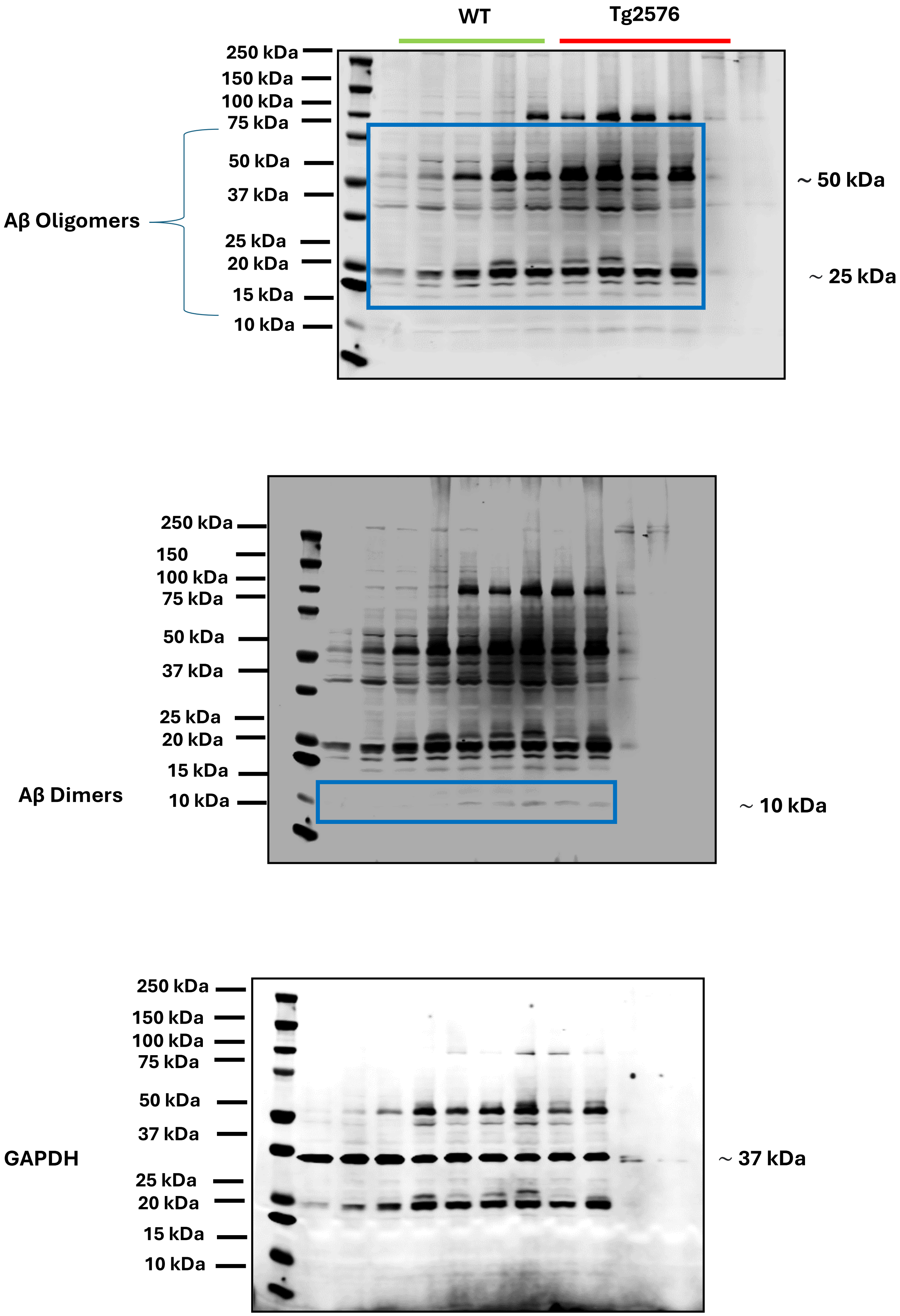
WT
Tg2576
250 kDa
150 kDa
100 kDa
75 kDa
50 kDa
37 kDa
25 kDa
20 kDa
15 kDa
10 kDa
Aβ Oligomers
~ 50 kDa
~ 25 kDa
250 kDa
150
100 kDa
75 kDa
50 kDa
37 kDa
25 kDa
20 kDa
15 kDa
10 kDa
Aβ Dimers
~ 10 kDa
250 kDa
150 kDa
100 kDa
75 kDa
50 kDa
37 kDa
25 kDa
20 kDa
15 kDa
10 kDa
GAPDH
~ 37 kDa

Figure 1K

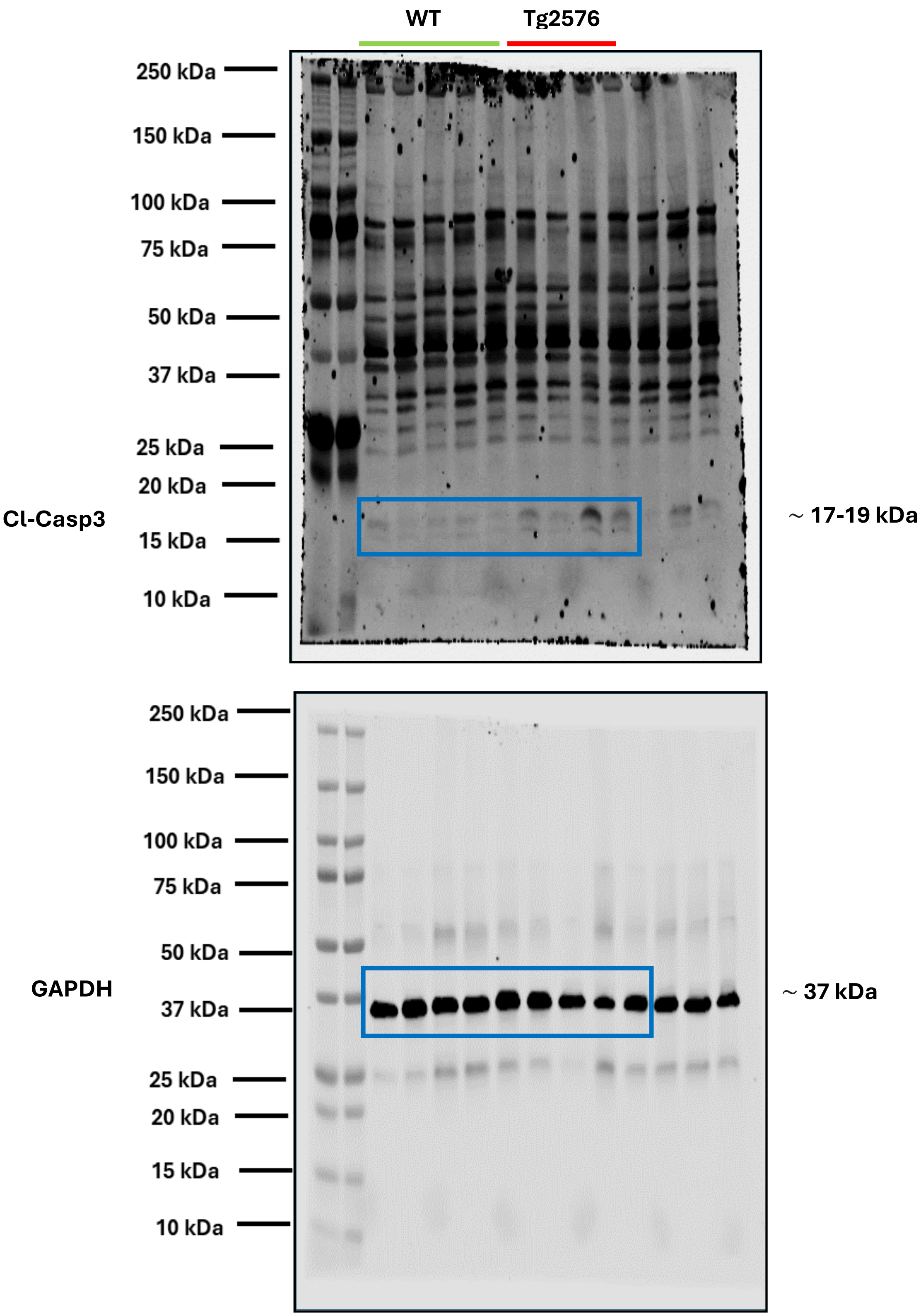

WT
Tg2576
250 kDa
150 kDa
100 kDa
75 kDa
50 kDa
37 kDa
25 kDa
20 kDa
15 kDa
10 kDa
Cl-Casp3
~ 17-19 kDa
250 kDa
150 kDa
100 kDa
75 kDa
50 kDa
37 kDa
25 kDa
20 kDa
15 kDa
10 kDa
GAPDH
~ 37 kDa

Figure 1O

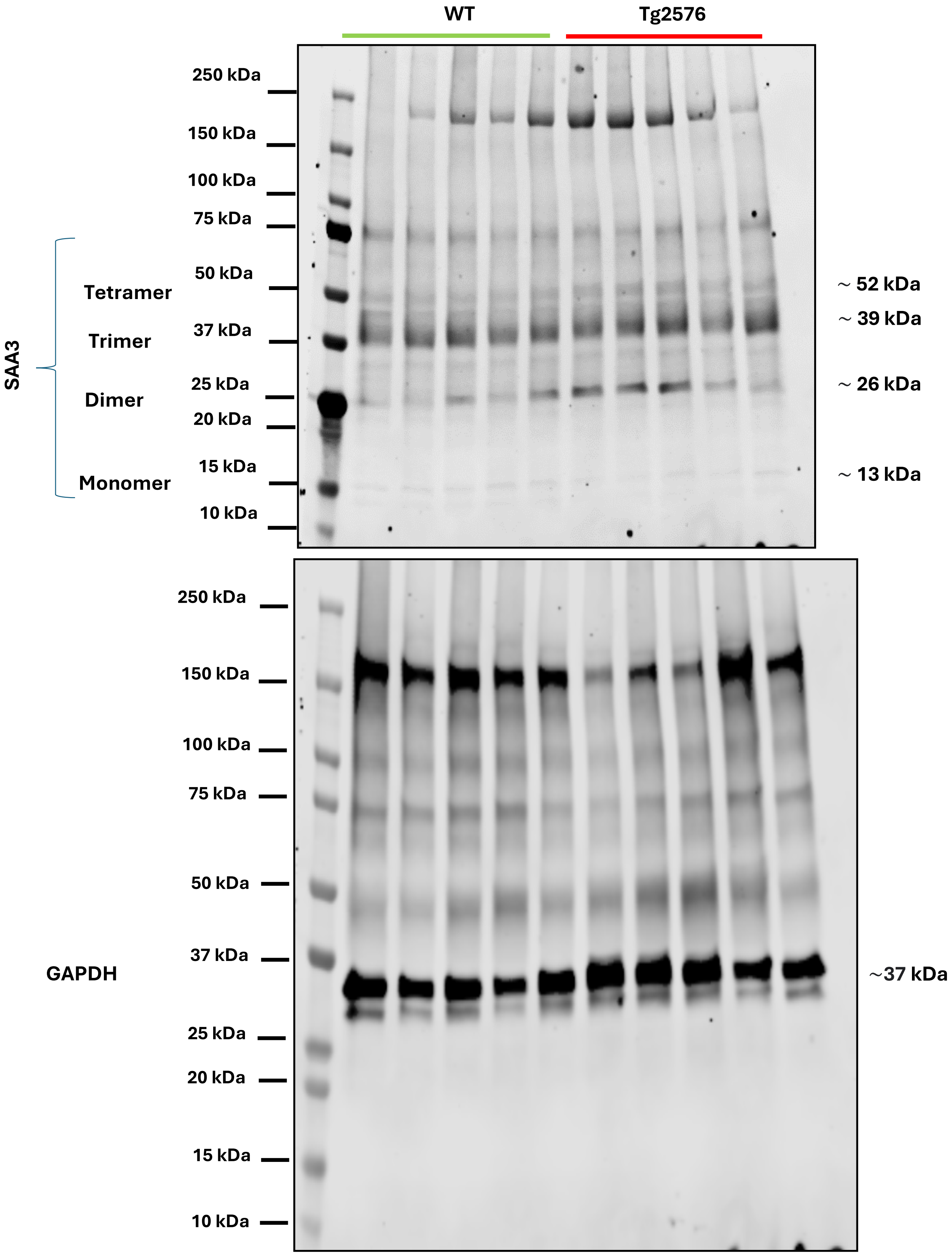
WT
Tg2576
250 kDa
150 kDa
100 kDa
75 kDa
50 kDa
37 kDa
25 kDa
20 kDa
15 kDa
10 kDa
SAA3
Tetramer
Trimer
Dimer
Monomer
~ 52 kDa
~ 39 kDa
~ 26 kDa
~ 13 kDa
GAPDH
250 kDa
150 kDa
100 kDa
75 kDa
50 kDa
37 kDa
25 kDa
20 kDa
15 kDa
10 kDa
~37 kDa

Figure 2A - Heart tissue

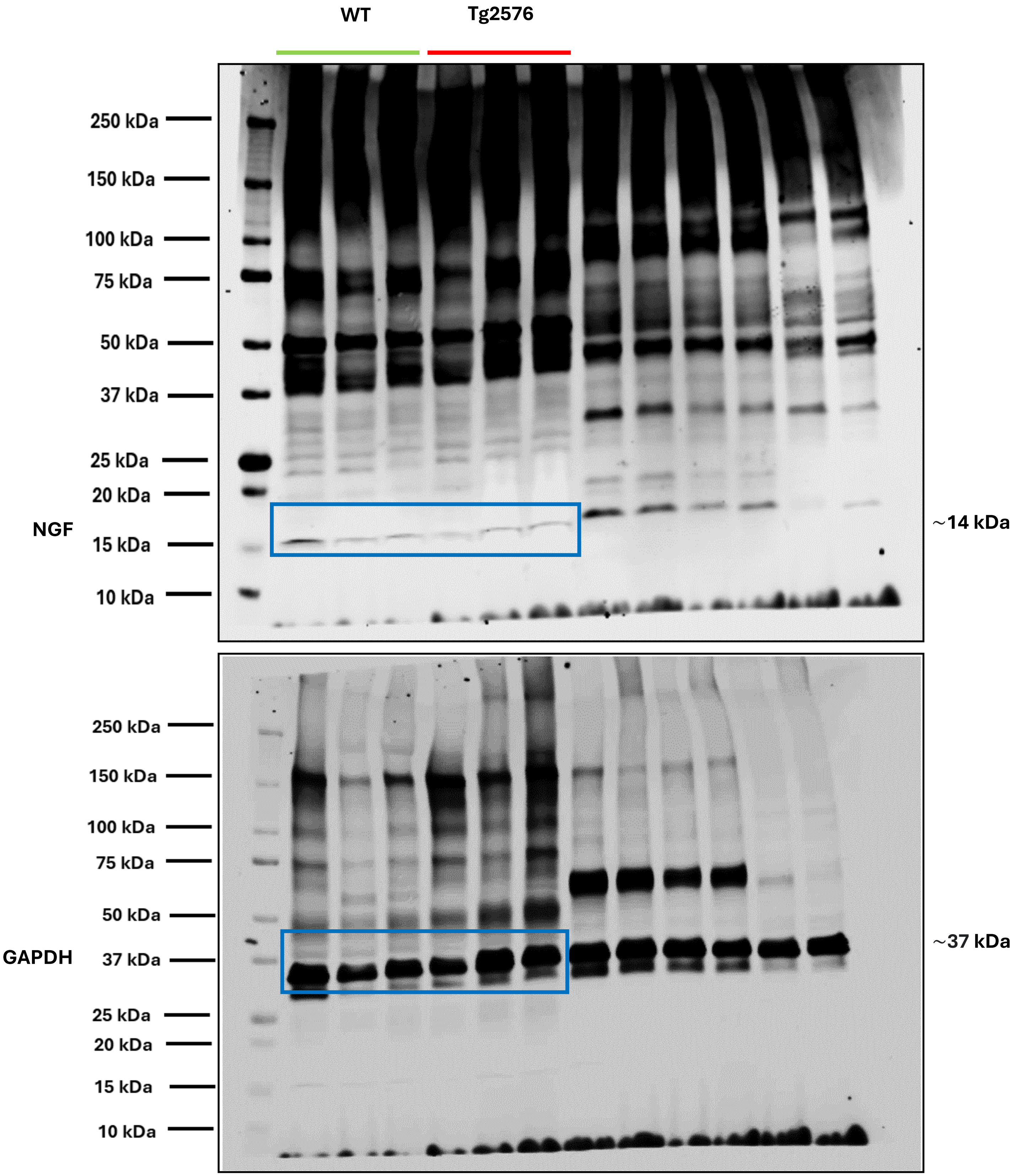

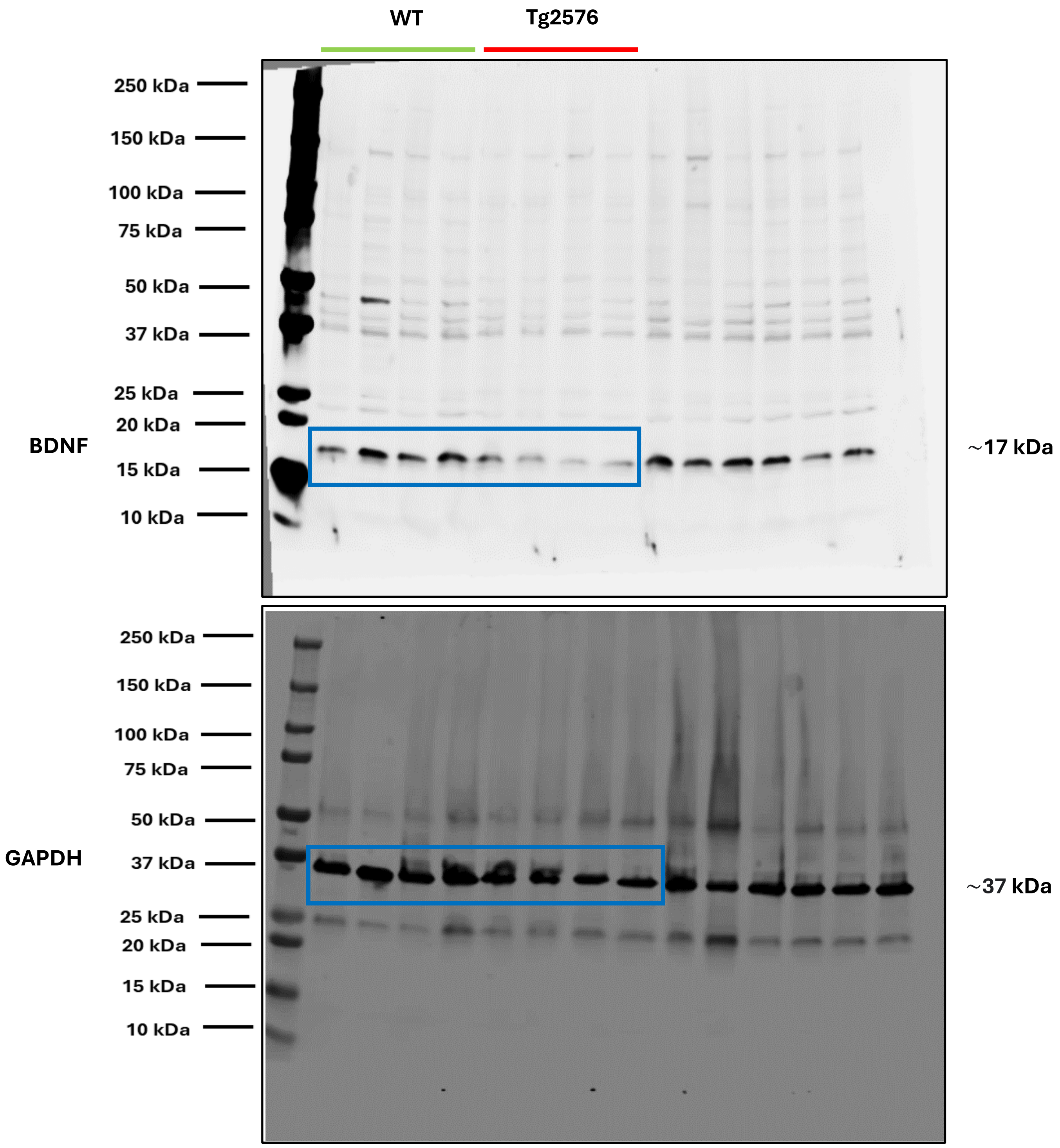
WT
Tg2576
250 kDa
150 kDa
100 kDa
75 kDa
50 kDa
37 kDa
25 kDa
20 kDa
BDNF
15 kDa
10 kDa
~17 kDa
250 kDa
150 kDa
100 kDa
75 kDa
50 kDa
GAPDH
37 kDa
25 kDa
20 kDa
15 kDa
10 kDa
~37 kDa

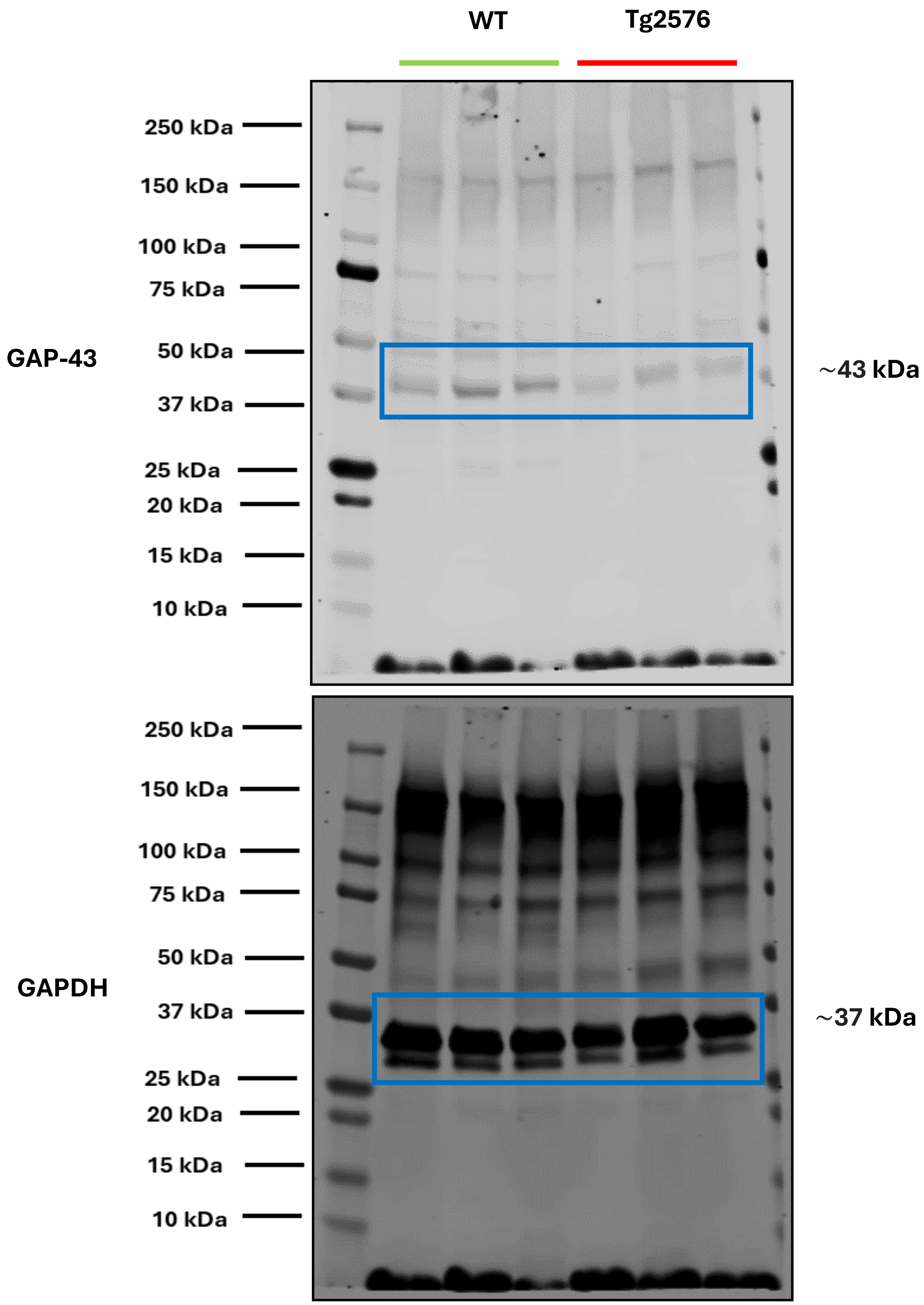
WT
Tg2576
GAP-43
250 kDa
150 kDa
100 kDa
75 kDa
50 kDa
37 kDa
25 kDa
20 kDa
15 kDa
10 kDa
~43 kDa
GAPDH
250 kDa
150 kDa
100 kDa
75 kDa
50 kDa
37 kDa
25 kDa
20 kDa
15 kDa
10 kDa
~37 kDa

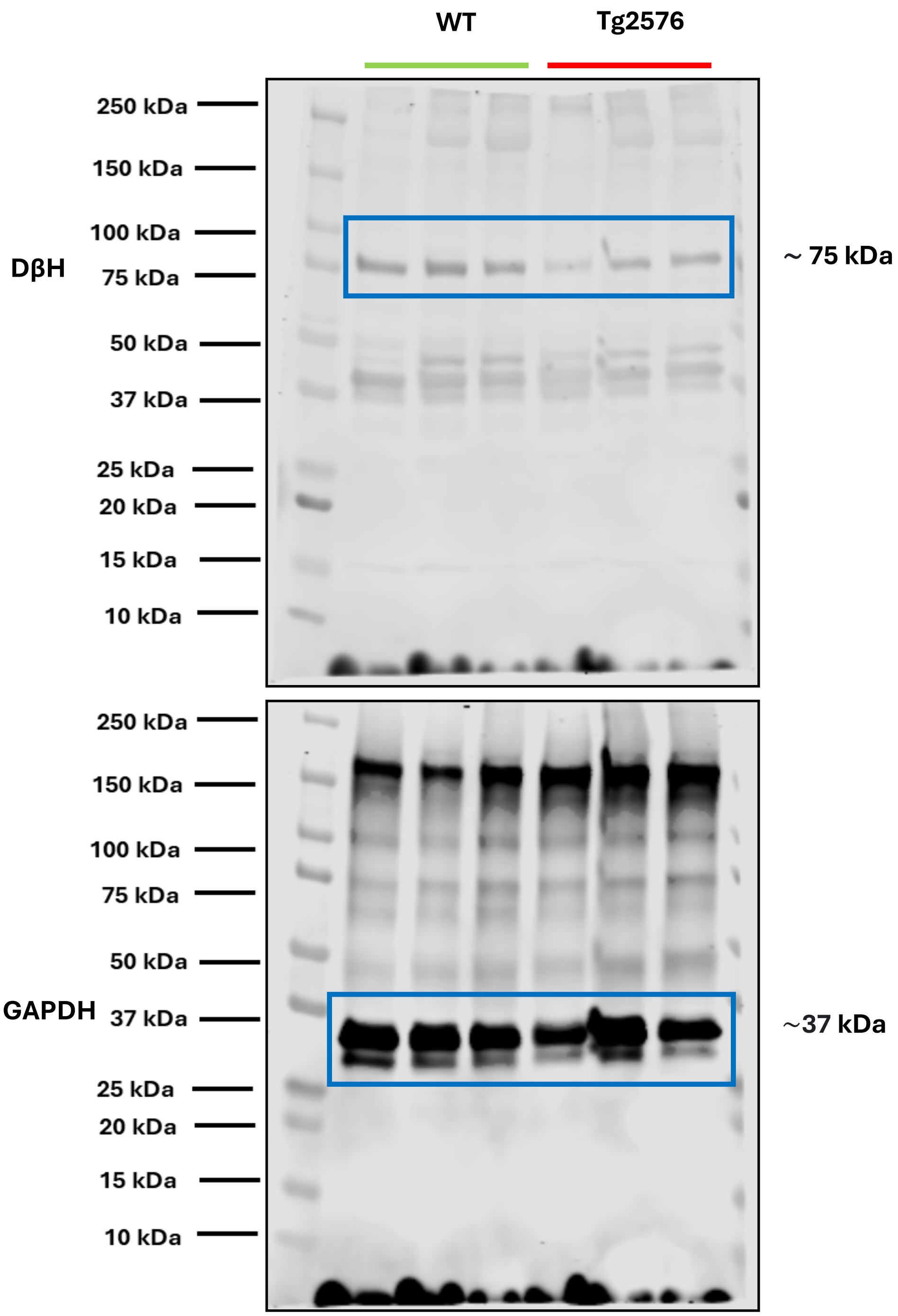
WT
Tg2576
DβH
250 kDa
150 kDa
100 kDa
75 kDa
50 kDa
37 kDa
25 kDa
20 kDa
15 kDa
10 kDa
~ 75 kDa
GAPDH
250 kDa
150 kDa
100 kDa
75 kDa
50 kDa
37 kDa
25 kDa
20 kDa
15 kDa
10 kDa
~37 kDa

Figure 2A - Cerebral Cortex

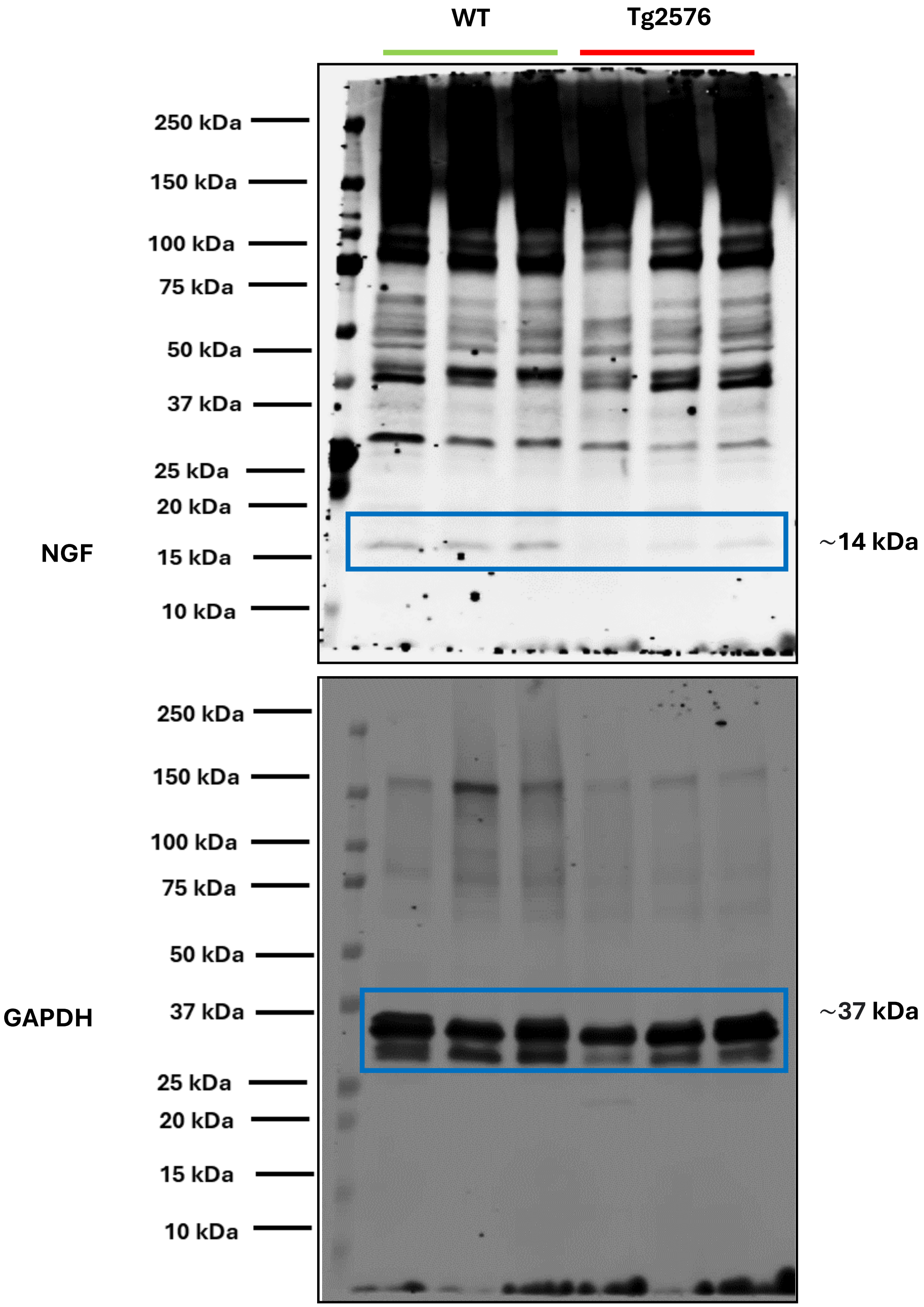

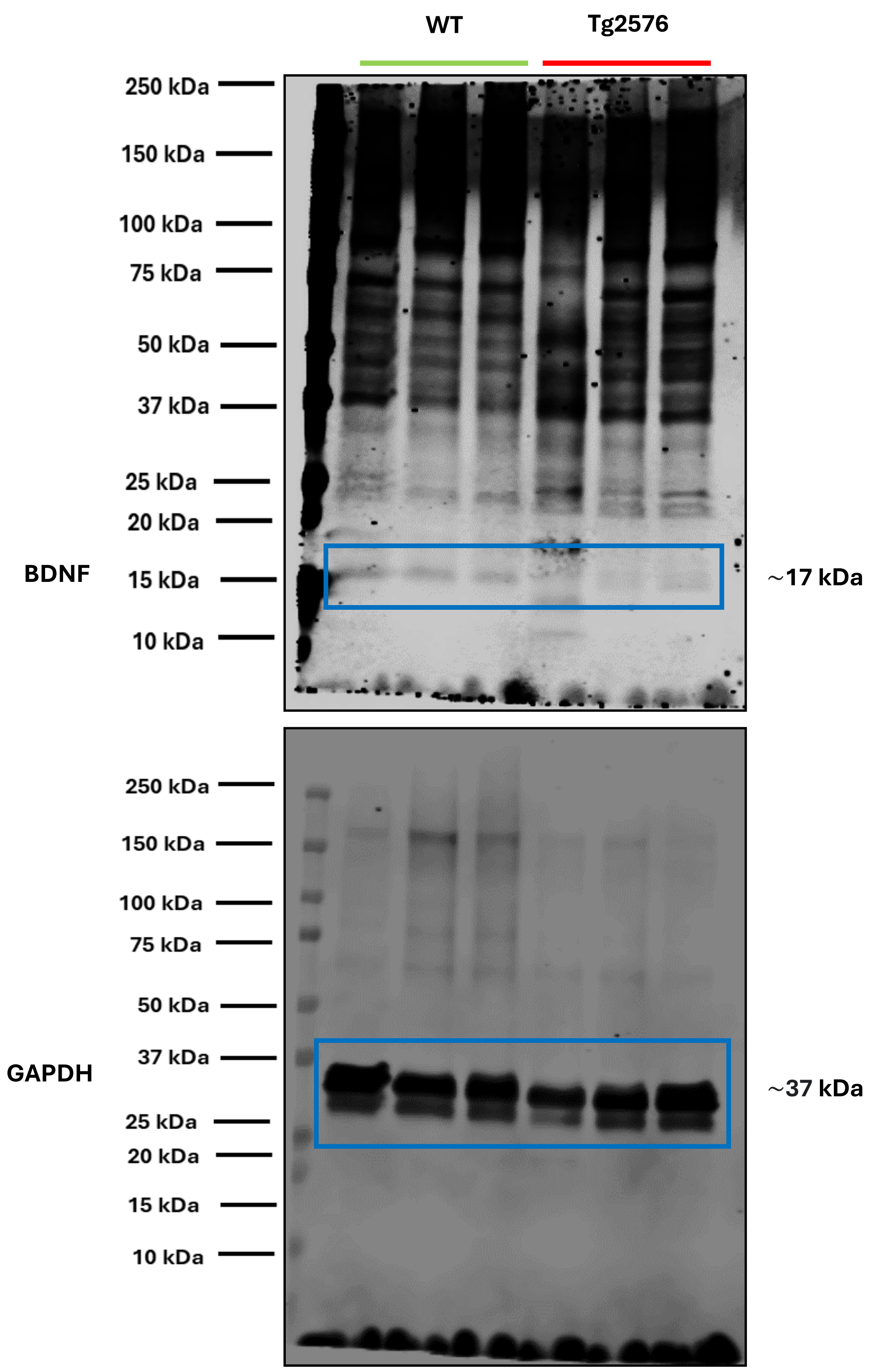
WT
Tg2576
250 kDa
150 kDa
100 kDa
75 kDa
50 kDa
37 kDa
25 kDa
20 kDa
15 kDa
10 kDa
BDNF
~17 kDa
250 kDa
150 kDa
100 kDa
75 kDa
50 kDa
37 kDa
25 kDa
20 kDa
15 kDa
10 kDa
GAPDH
~37 kDa

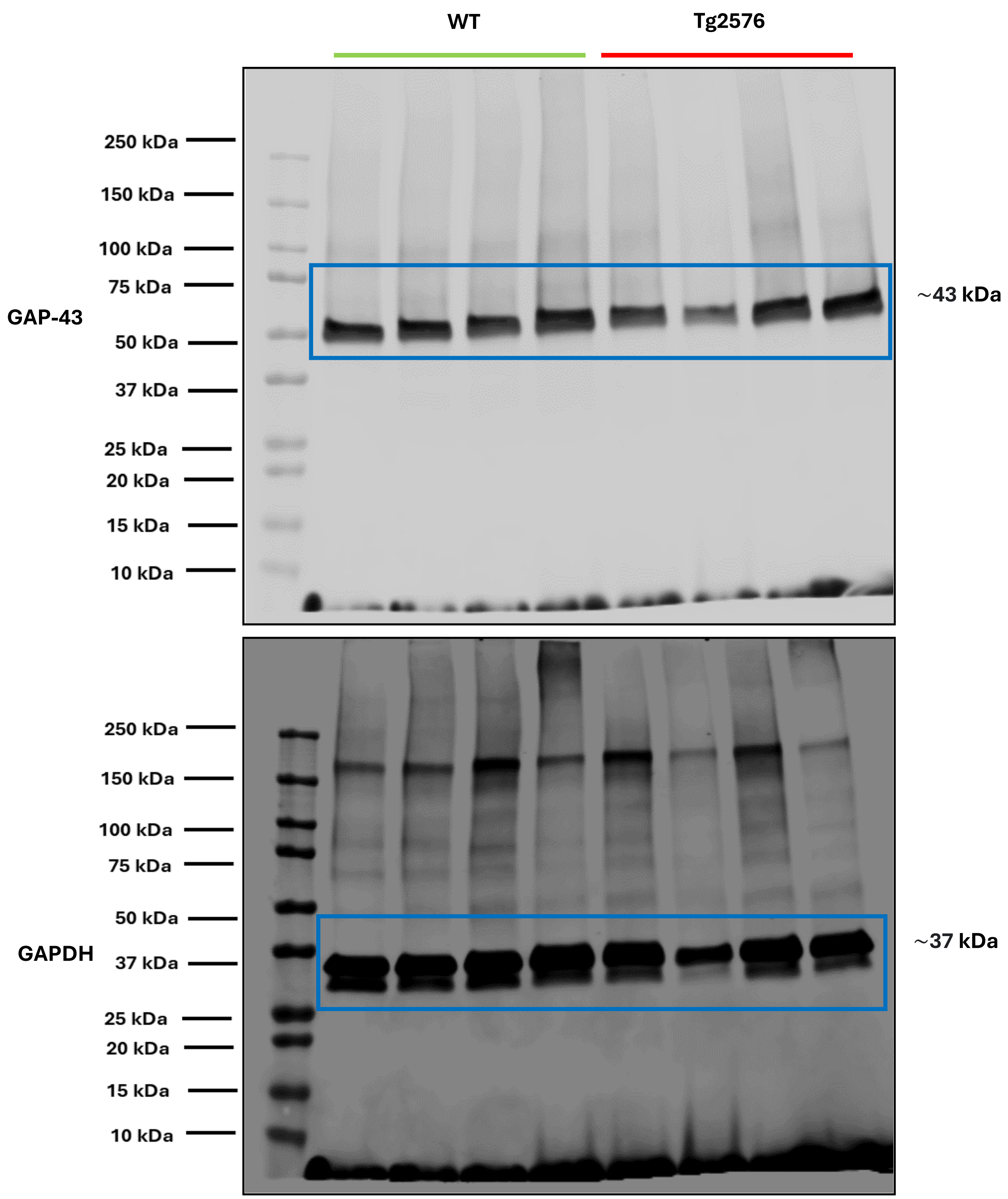
WT
Tg2576
GAP-43
250 kDa
150 kDa
100 kDa
75 kDa
50 kDa
37 kDa
25 kDa
20 kDa
15 kDa
10 kDa
~43 kDa
GAPDH
250 kDa
150 kDa
100 kDa
75 kDa
50 kDa
37 kDa
25 kDa
20 kDa
15 kDa
10 kDa
~37 kDa

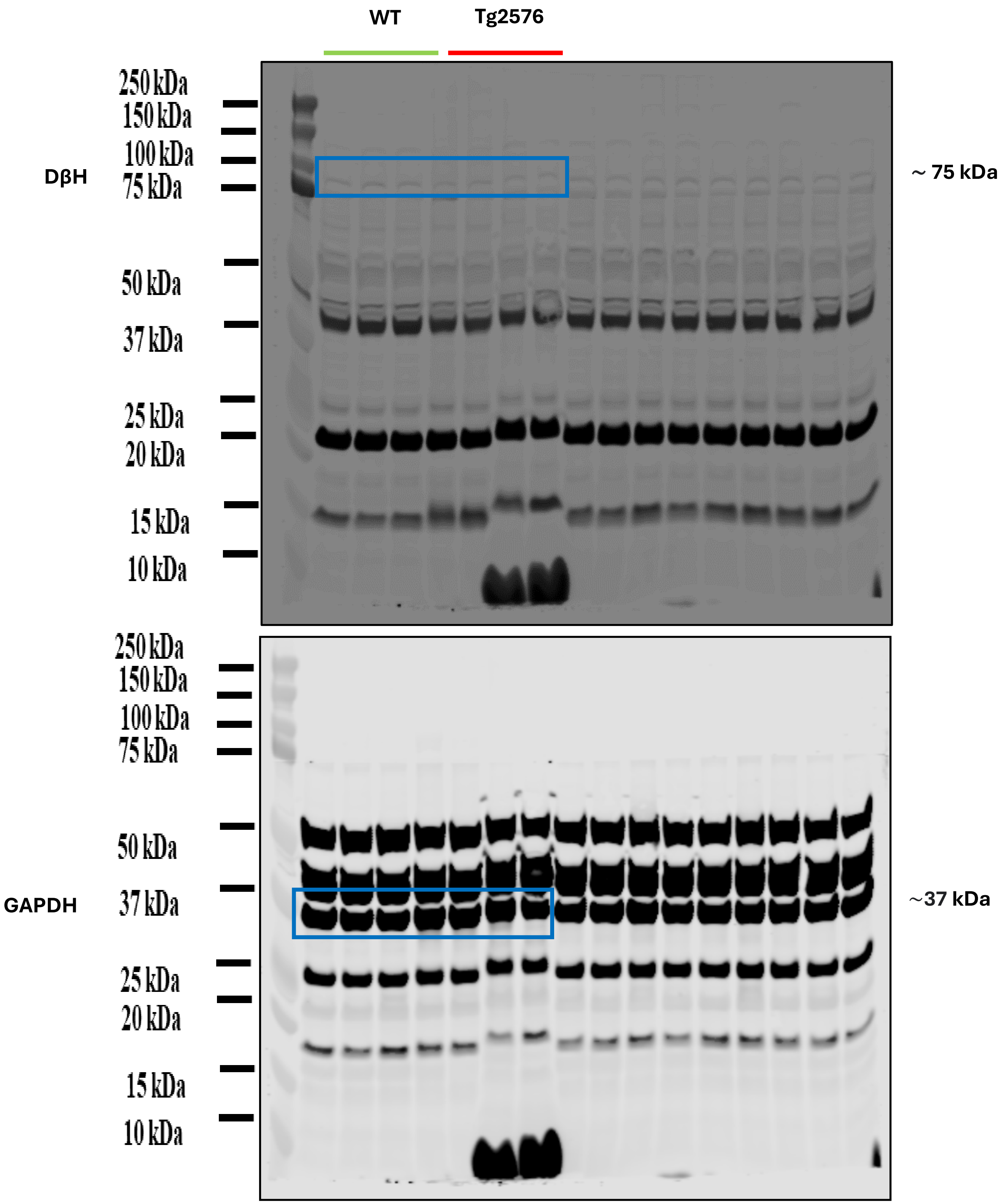

WT
Tg2576
DβH
250 kDa
150 kDa
100 kDa
75 kDa
50 kDa
37 kDa
25 kDa
20 kDa
15 kDa
10 kDa
~ 75 kDa
GAPDH
250 kDa
150 kDa
100 kDa
75 kDa
50 kDa
37 kDa
25 kDa
20 kDa
15 kDa
10 kDa
~37 kDa

Figure 3A

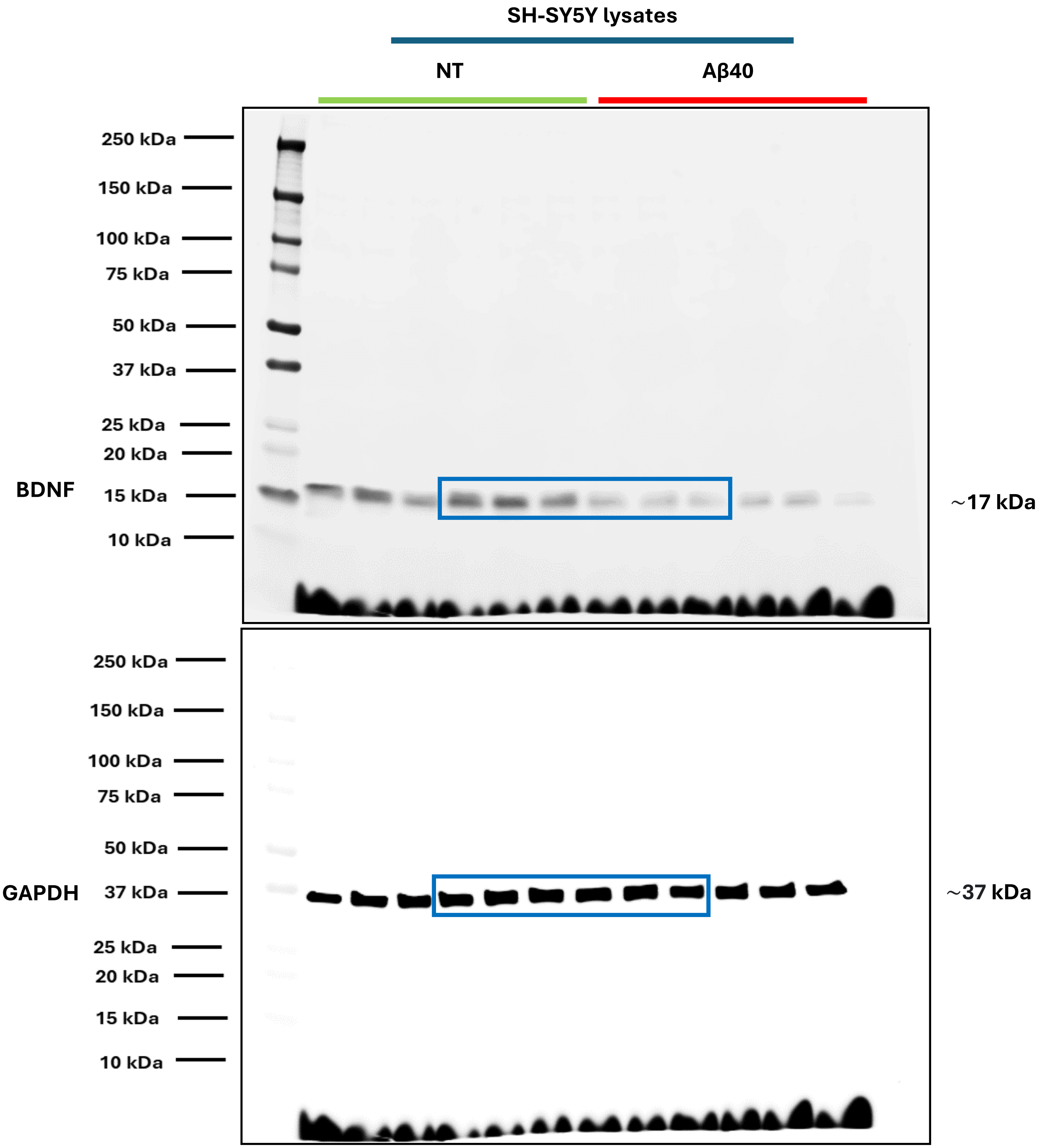

SH-SY5Y lysates
NT
Aβ40
250 kDa
150 kDa
100 kDa
75 kDa
50 kDa
37 kDa
25 kDa
20 kDa
BDNF
15 kDa
10 kDa
~17 kDa
250 kDa
150 kDa
100 kDa
75 kDa
50 kDa
GAPDH
37 kDa
25 kDa
20 kDa
15 kDa
10 kDa
~37 kDa

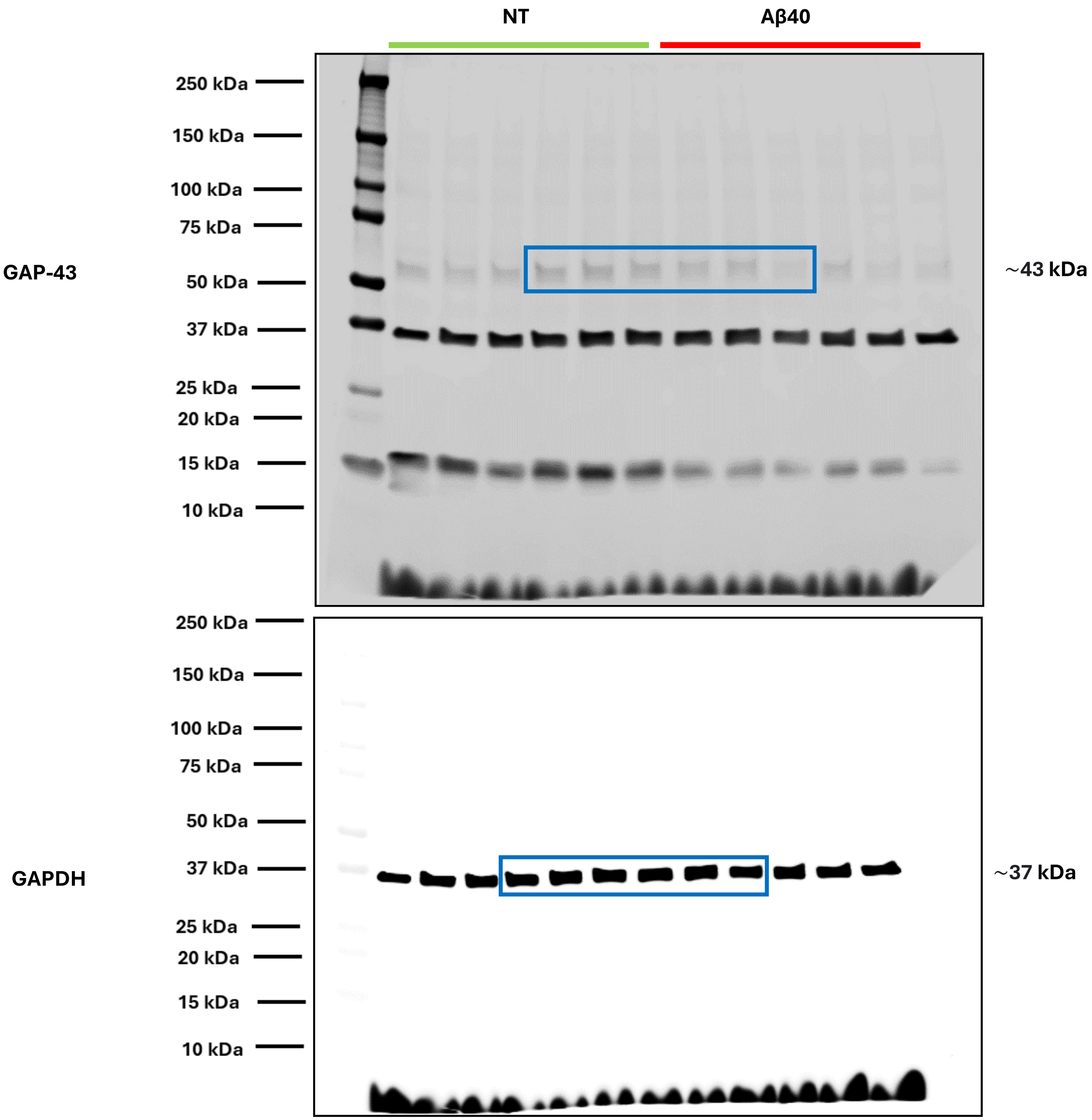

NT
Aβ40
GAP-43
250 kDa
150 kDa
100 kDa
75 kDa
50 kDa
37 kDa
25 kDa
20 kDa
15 kDa
10 kDa
~43 kDa
250 kDa
150 kDa
100 kDa
75 kDa
50 kDa
37 kDa
25 kDa
20 kDa
15 kDa
10 kDa
GAPDH
~37 kDa

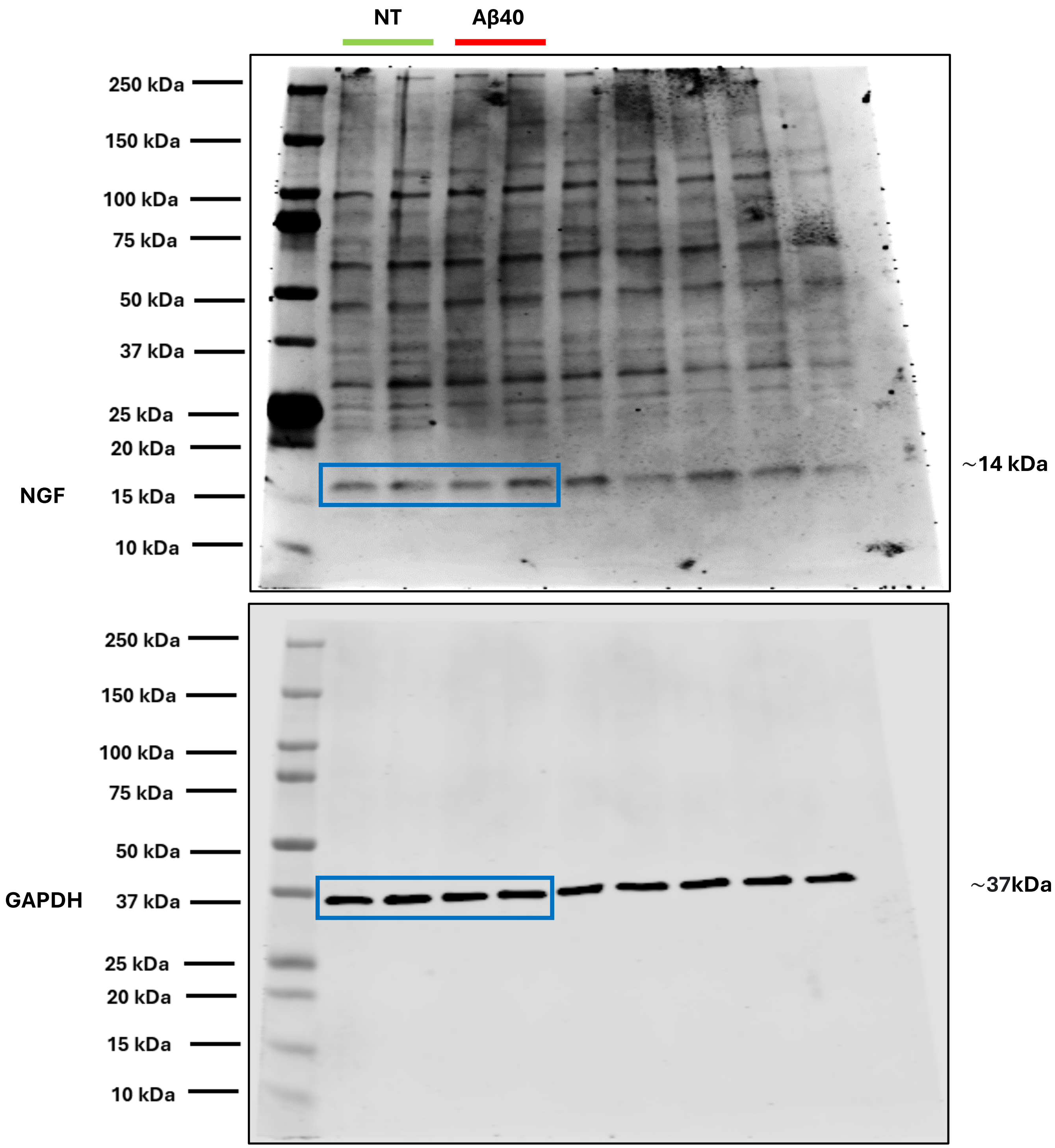

NT
Aβ40
250 kDa
150 kDa
100 kDa
75 kDa
50 kDa
37 kDa
25 kDa
20 kDa
15 kDa
10 kDa
NGF
~14 kDa
250 kDa
150 kDa
100 kDa
75 kDa
50 kDa
37 kDa
25 kDa
20 kDa
15 kDa
10 kDa
GAPDH
~37kDa

Figure 3C

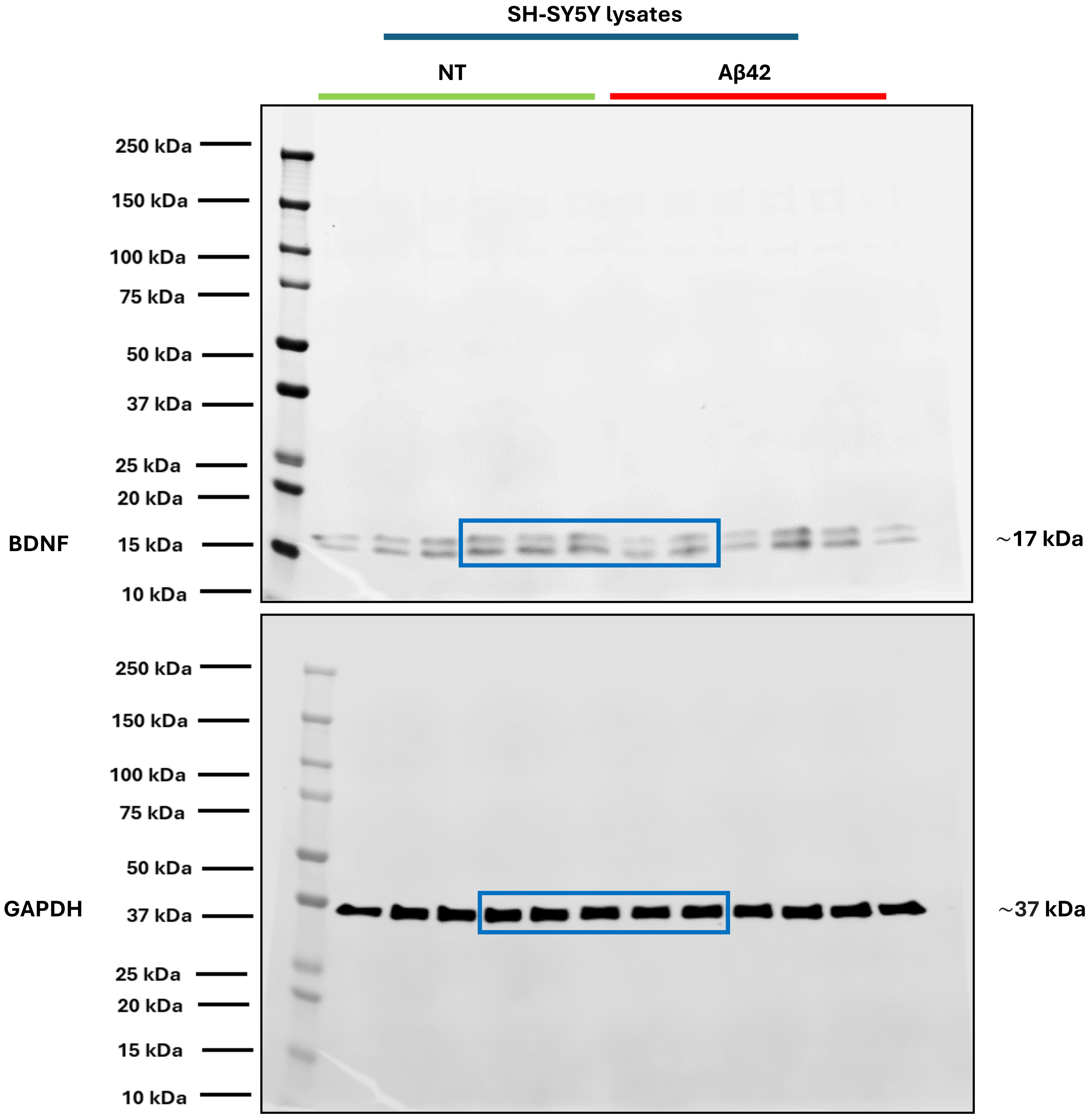
SH-SY5Y lysates
NT
Aβ42
250 kDa
150 kDa
100 kDa
75 kDa
50 kDa
37 kDa
25 kDa
20 kDa
BDNF
15 kDa
10 kDa
~17 kDa
250 kDa
150 kDa
100 kDa
75 kDa
50 kDa
GAPDH
37 kDa
25 kDa
20 kDa
15 kDa
10 kDa
~37 kDa

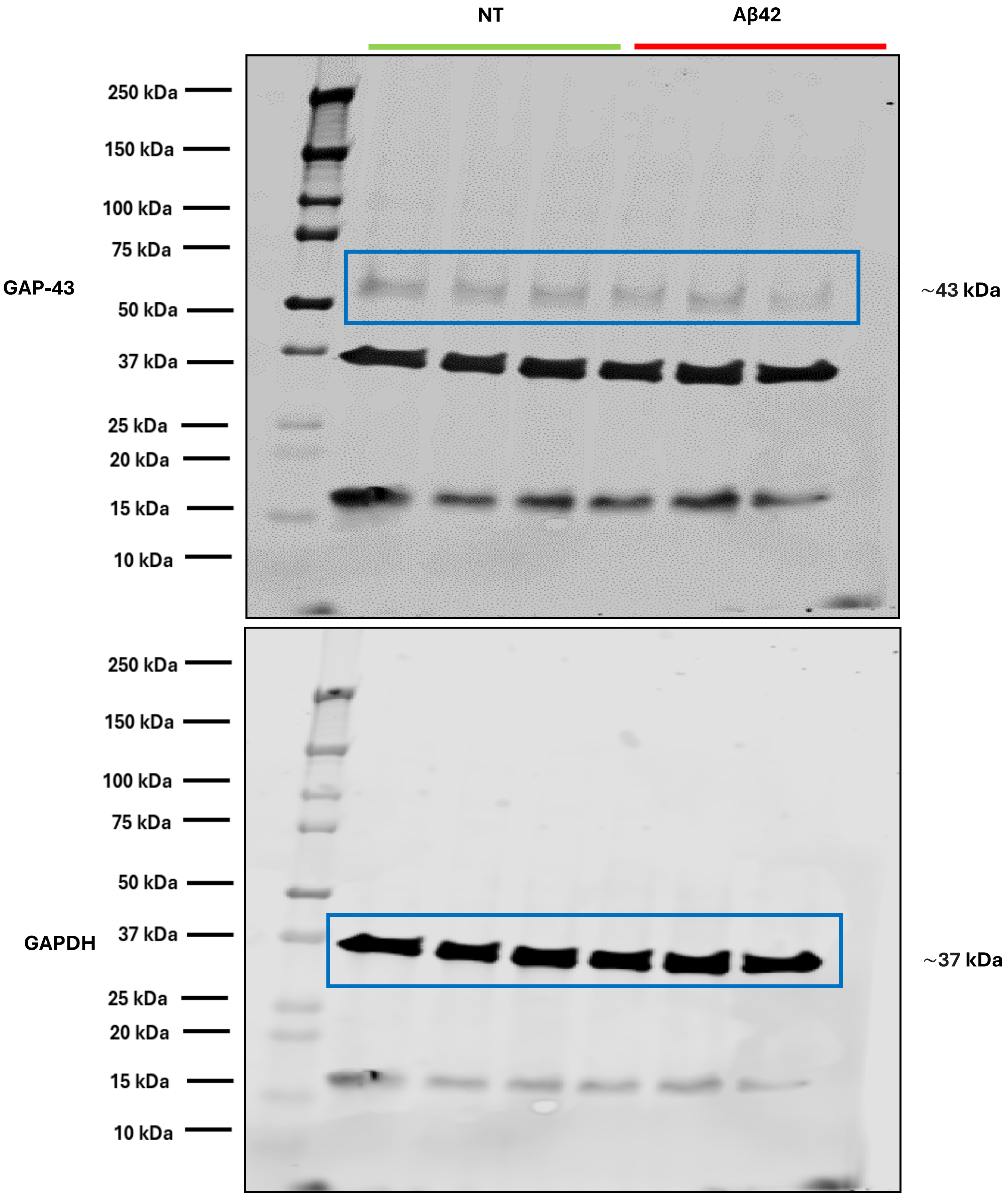
NT
Aβ42
250 kDa
150 kDa
100 kDa
75 kDa
GAP-43
50 kDa
37 kDa
25 kDa
20 kDa
15 kDa
10 kDa
~43 kDa
250 kDa
150 kDa
100 kDa
75 kDa
50 kDa
GAPDH
37 kDa
25 kDa
20 kDa
15 kDa
10 kDa
~37 kDa

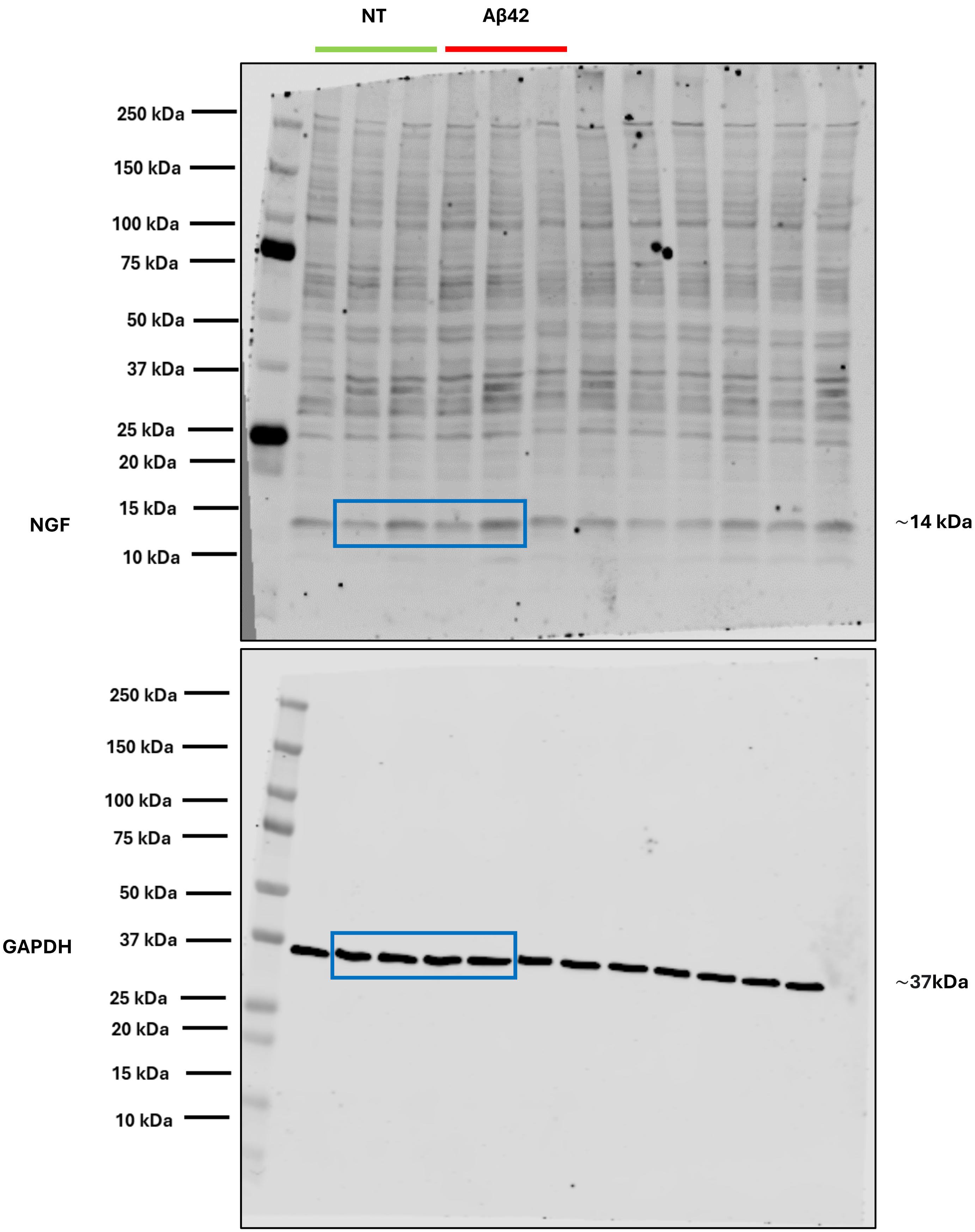
NT
Aβ42
250 kDa
150 kDa
100 kDa
75 kDa
50 kDa
37 kDa
25 kDa
20 kDa
15 kDa
10 kDa
NGF
~14 kDa
250 kDa
150 kDa
100 kDa
75 kDa
50 kDa
37 kDa
25 kDa
20 kDa
15 kDa
10 kDa
GAPDH
~37kDa

Figure 3E

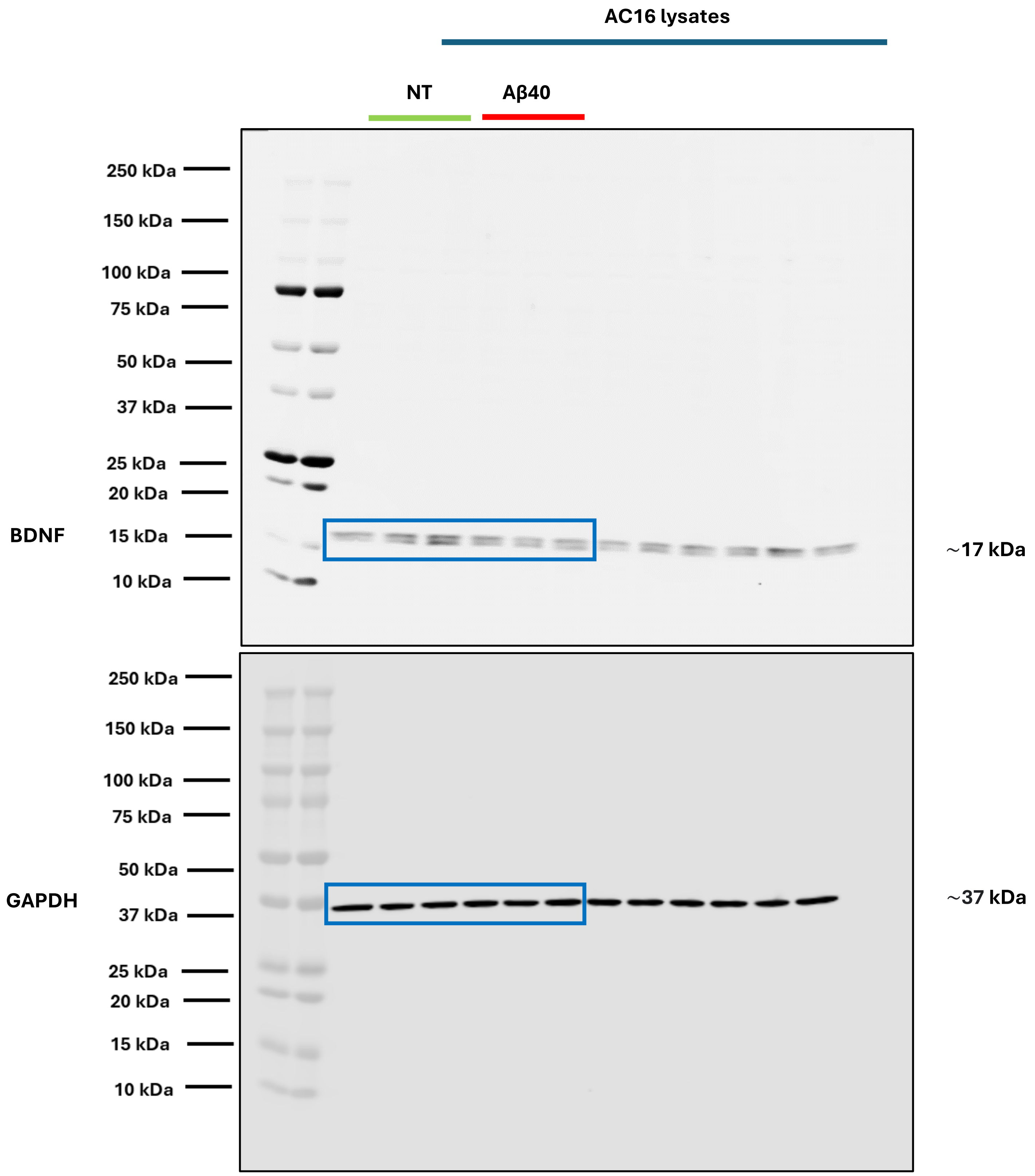
AC16 lysates
NT
Aβ40
250 kDa
150 kDa
100 kDa
75 kDa
50 kDa
37 kDa
25 kDa
20 kDa
BDNF
15 kDa
10 kDa
~17 kDa
250 kDa
150 kDa
100 kDa
75 kDa
50 kDa
GAPDH
37 kDa
25 kDa
20 kDa
15 kDa
10 kDa
~37 kDa

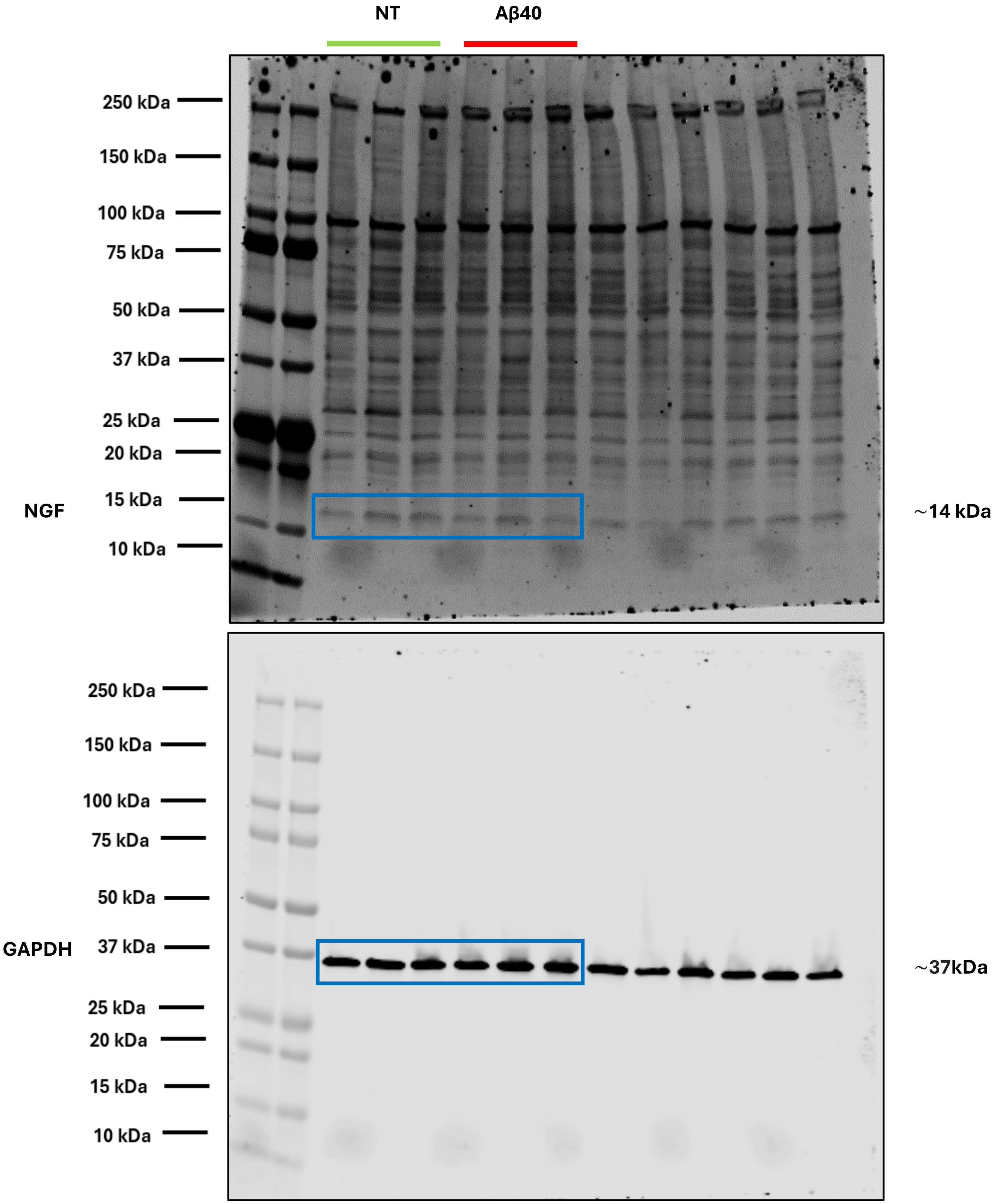
NT
Aβ40
250 kDa
150 kDa
100 kDa
75 kDa
50 kDa
37 kDa
25 kDa
20 kDa
15 kDa
10 kDa
NGF
~14 kDa
250 kDa
150 kDa
100 kDa
75 kDa
50 kDa
37 kDa
GAPDH
25 kDa
20 kDa
15 kDa
10 kDa
~37kDa

Figure 3G

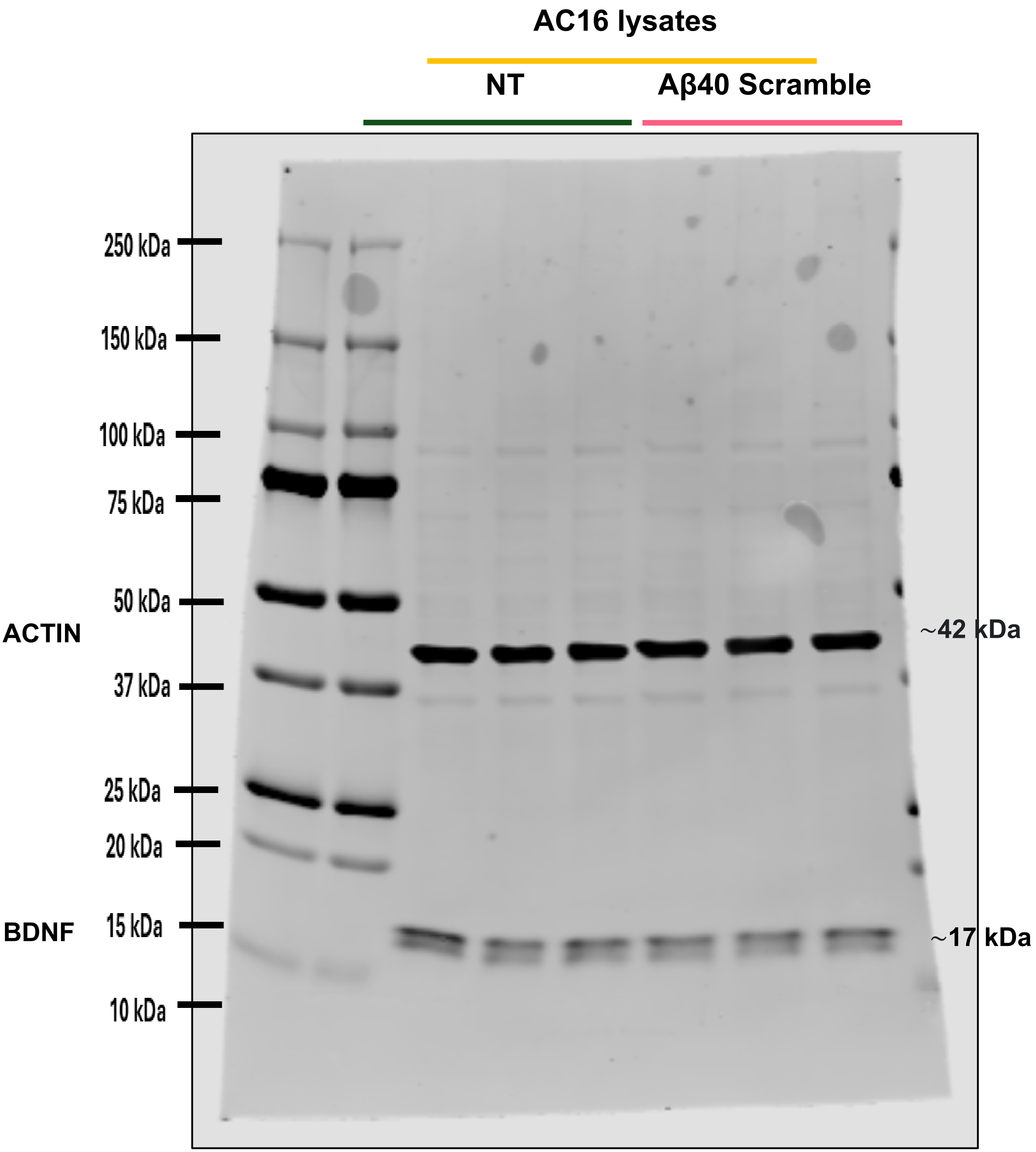
AC16 lysates
NT
Aβ40 Scramble
250 kDa
150 kDa
100 kDa
75 kDa
50 kDa
ACTIN
37 kDa
25 kDa
20 kDa
BDNF
15 kDa
10 kDa
~42 kDa
~17 kDa

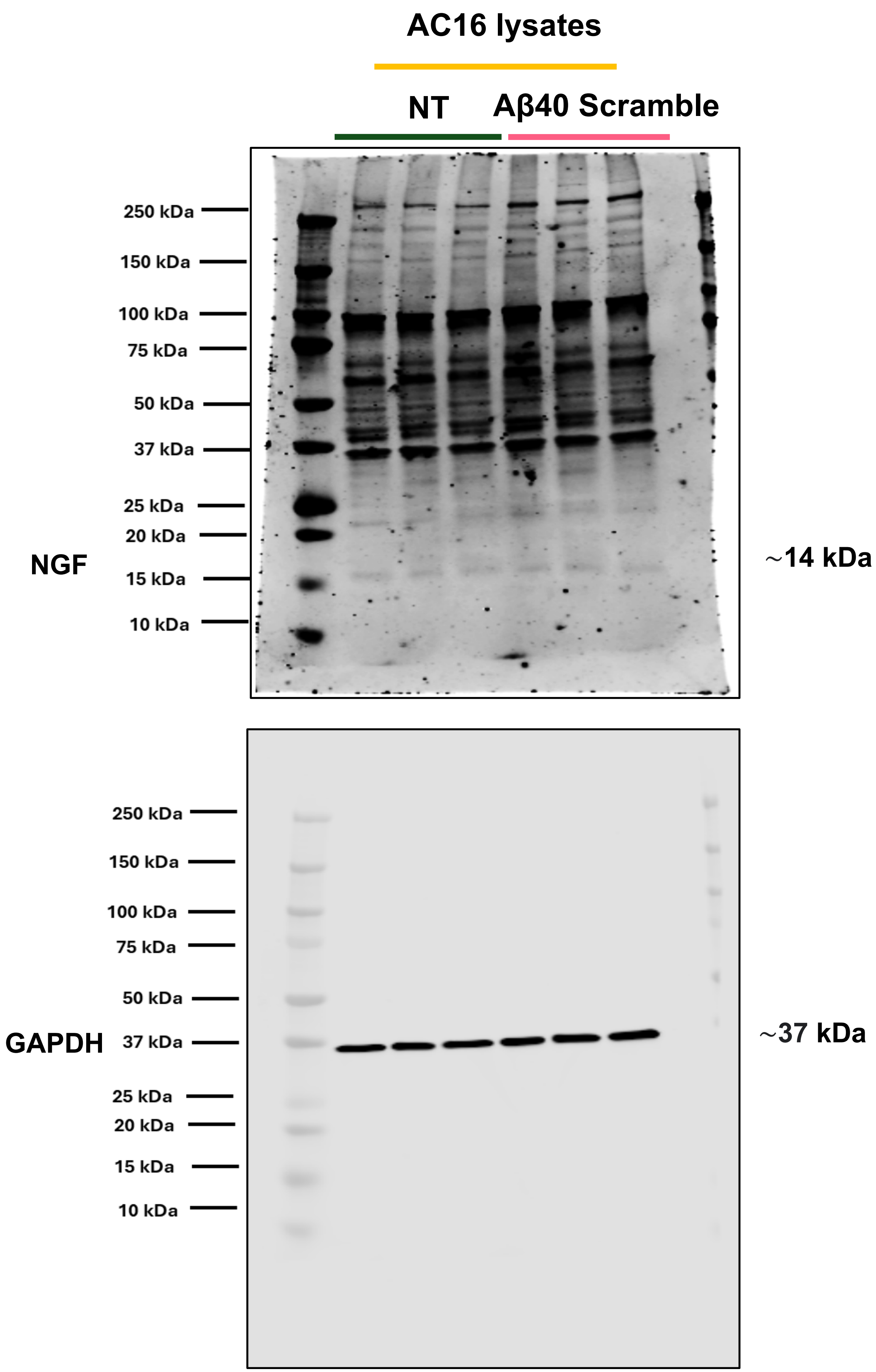
AC16 lysates
NT
Aβ40 Scramble
250 kDa
150 kDa
100 kDa
75 kDa
50 kDa
37 kDa
25 kDa
20 kDa
15 kDa
10 kDa
NGF
~14 kDa
250 kDa
150 kDa
100 kDa
75 kDa
50 kDa
37 kDa
25 kDa
20 kDa
15 kDa
10 kDa
GAPDH
~37 kDa

Figure 4A

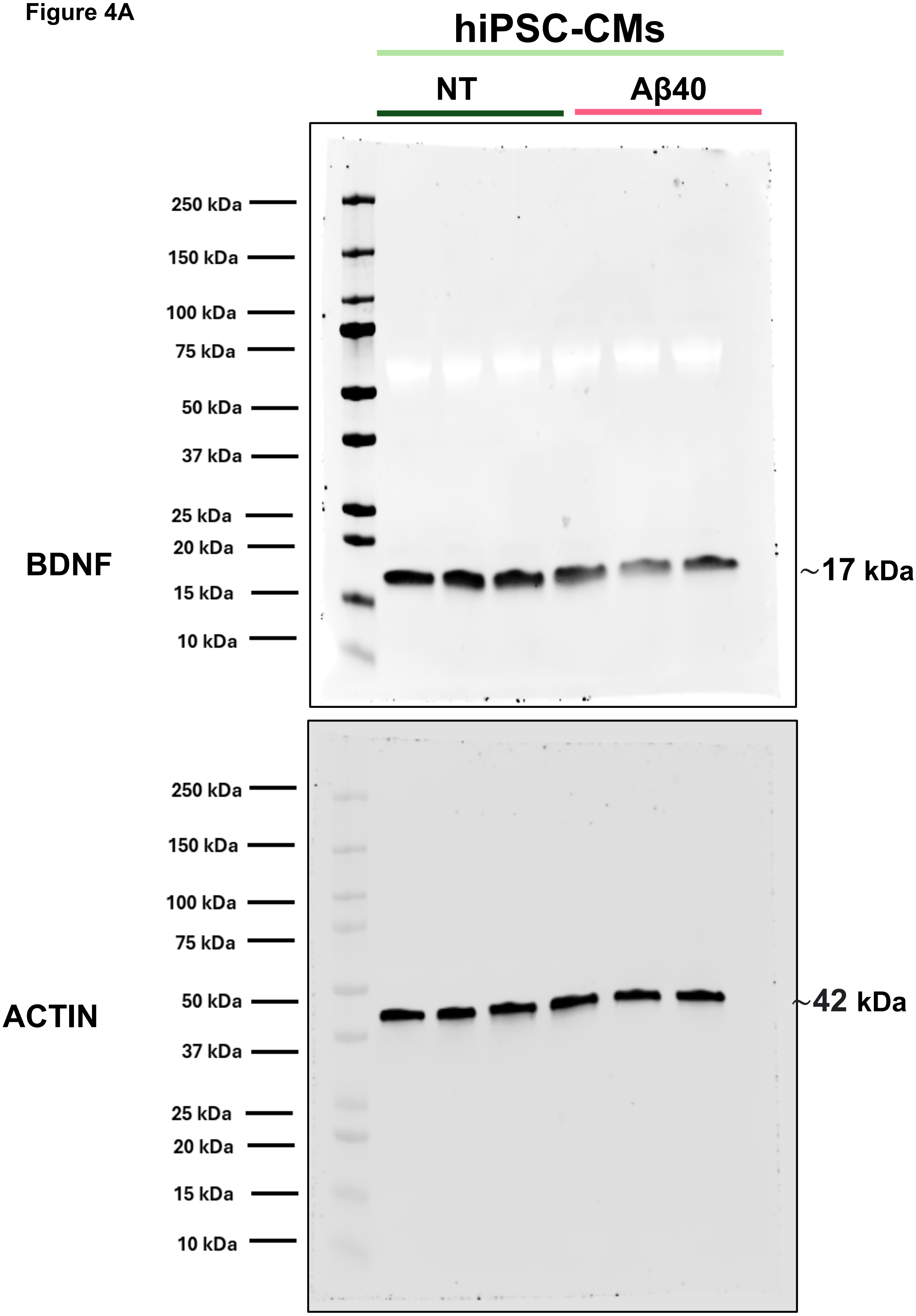

hiPSC-CMs
NT
Aβ40
250 kDa
150 kDa
100 kDa
75 kDa
50 kDa
37 kDa
25 kDa
20 kDa
15 kDa
10 kDa
BDNF
~17 kDa
250 kDa
150 kDa
100 kDa
75 kDa
50 kDa
37 kDa
25 kDa
20 kDa
15 kDa
10 kDa
ACTIN
~42 kDa

Figure 5A

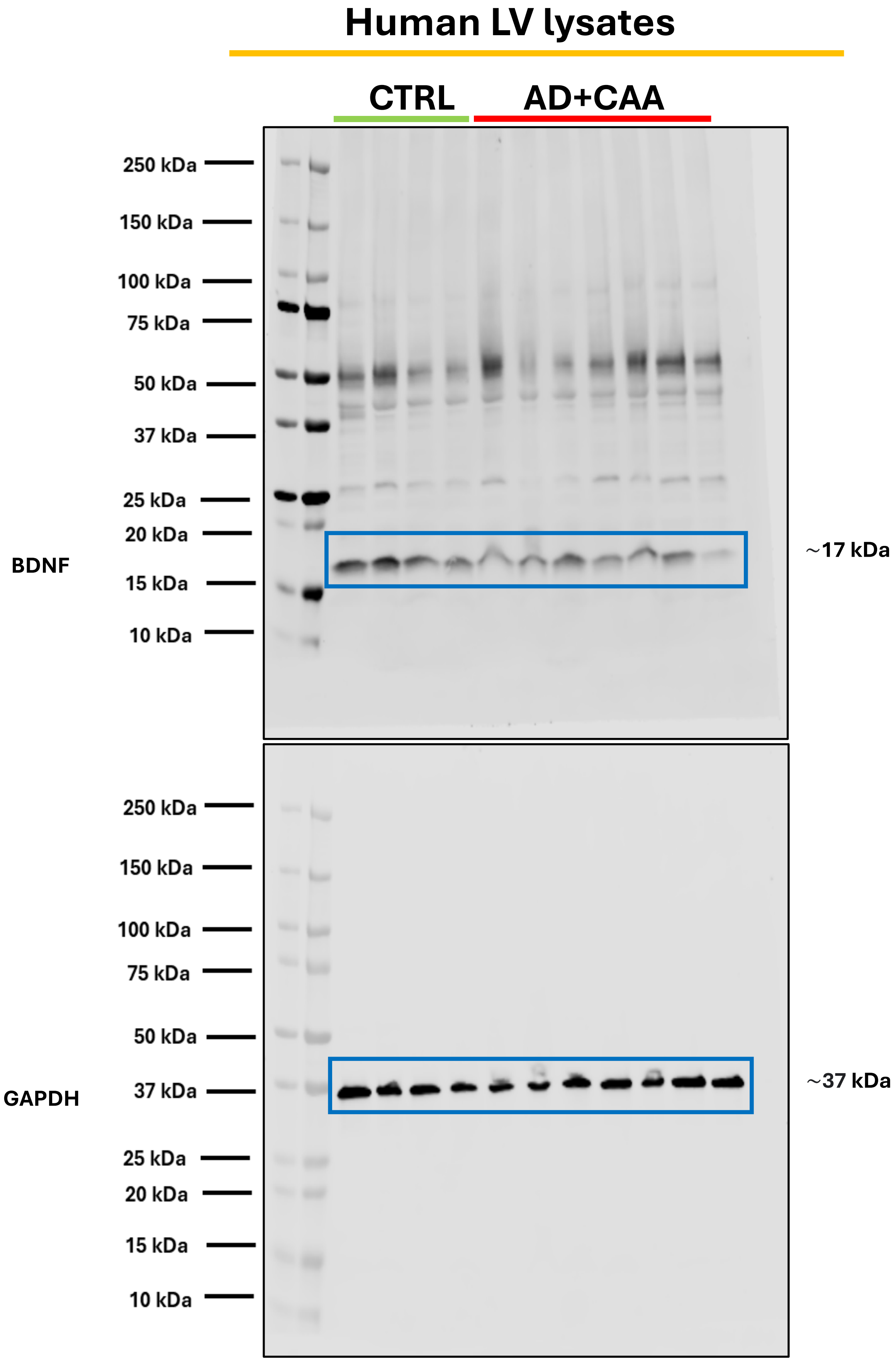

Human LV lysates
CTRL
AD+CAA
250 kDa
150 kDa
100 kDa
75 kDa
50 kDa
37 kDa
25 kDa
20 kDa
BDNF
15 kDa
10 kDa
~17 kDa
250 kDa
150 kDa
100 kDa
75 kDa
50 kDa
GAPDH
37 kDa
25 kDa
20 kDa
15 kDa
10 kDa
~37 kDa

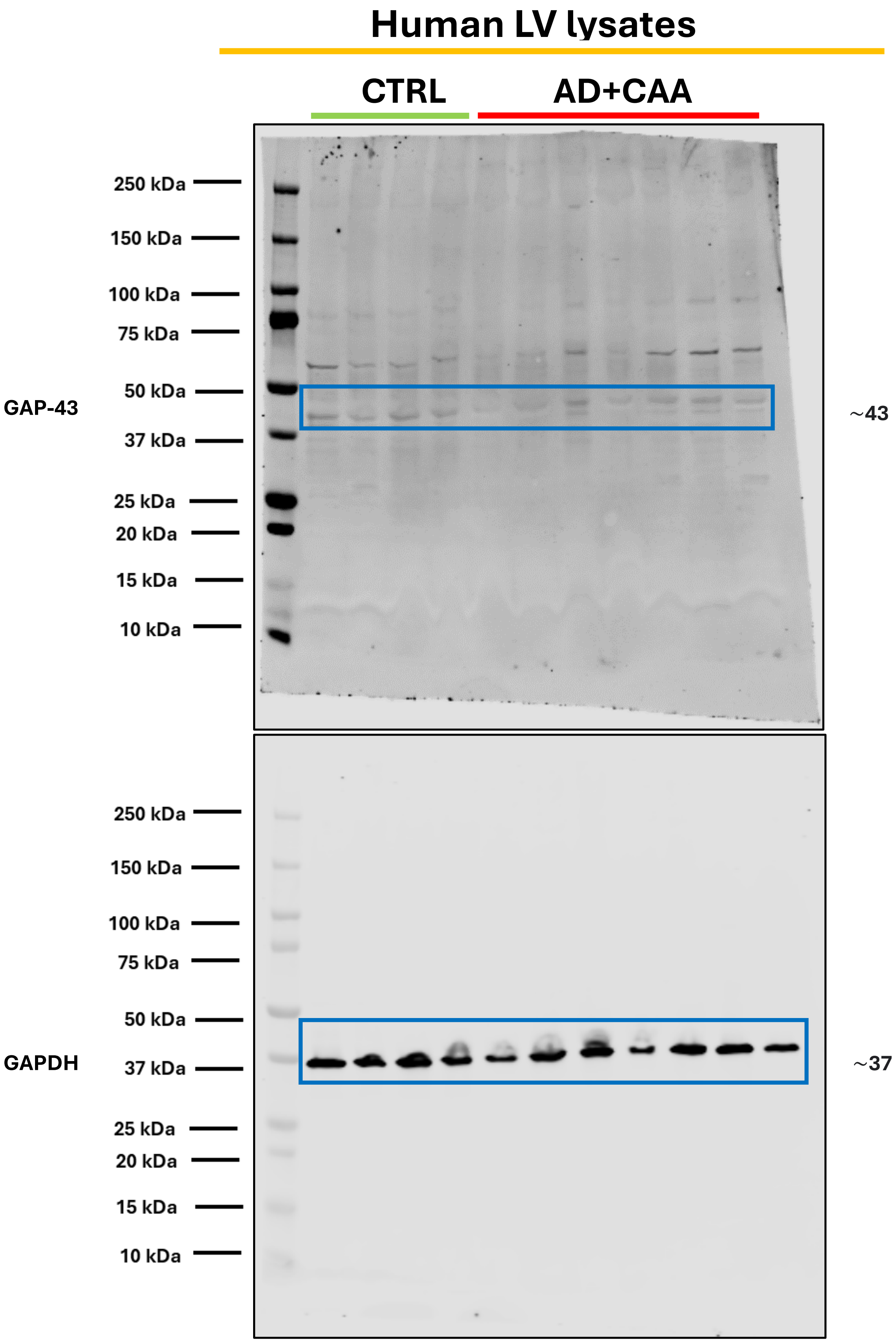
Human LV lysates
CTRL
AD+CAA
250 kDa
150 kDa
100 kDa
75 kDa
50 kDa
37 kDa
25 kDa
20 kDa
15 kDa
10 kDa
GAP-43
~43
250 kDa
150 kDa
100 kDa
75 kDa
50 kDa
37 kDa
25 kDa
20 kDa
15 kDa
10 kDa
GAPDH
~37

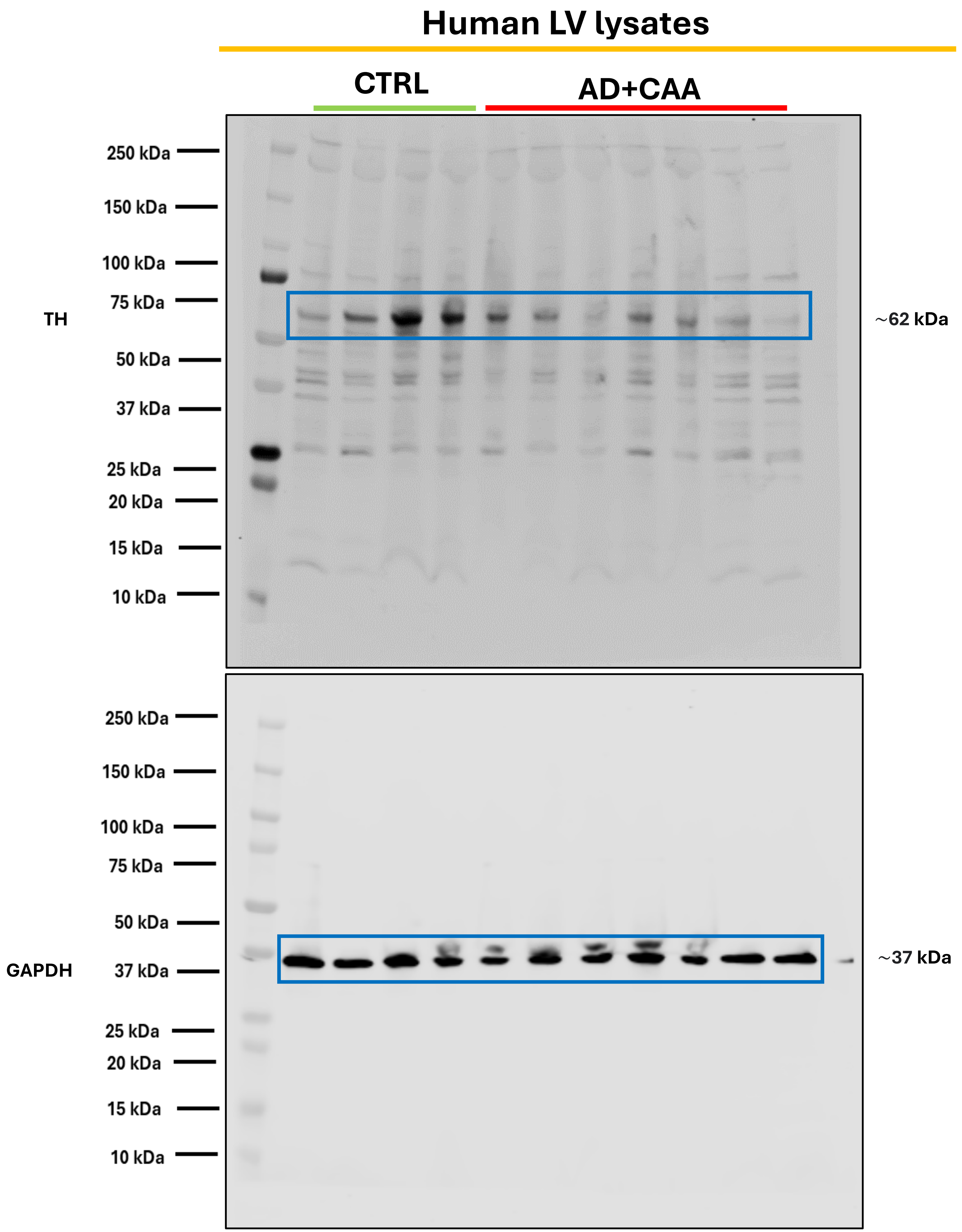

Human LV lysates
CTRL
AD+CAA
250 kDa
150 kDa
100 kDa
75 kDa
TH
50 kDa
37 kDa
25 kDa
20 kDa
15 kDa
10 kDa
~62 kDa
250 kDa
150 kDa
100 kDa
75 kDa
50 kDa
GAPDH
37 kDa
25 kDa
20 kDa
15 kDa
10 kDa
~37 kDa

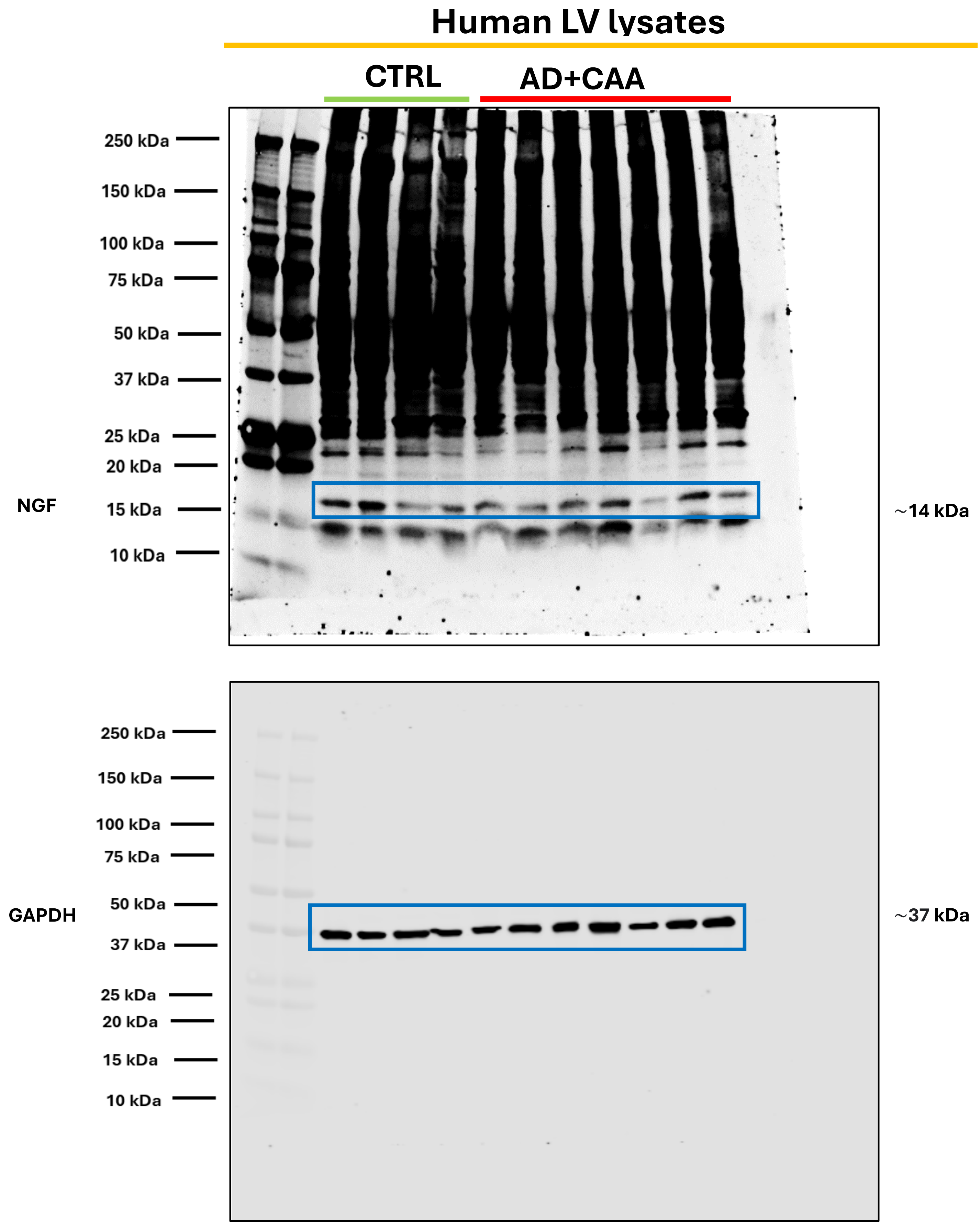
Human LV lysates
CTRL
AD+CAA
NGF
250 kDa
150 kDa
100 kDa
75 kDa
50 kDa
37 kDa
25 kDa
20 kDa
15 kDa
10 kDa
~14 kDa
GAPDH
250 kDa
150 kDa
100 kDa
75 kDa
50 kDa
37 kDa
25 kDa
20 kDa
15 kDa
10 kDa
~37 kDa

Figure 6A

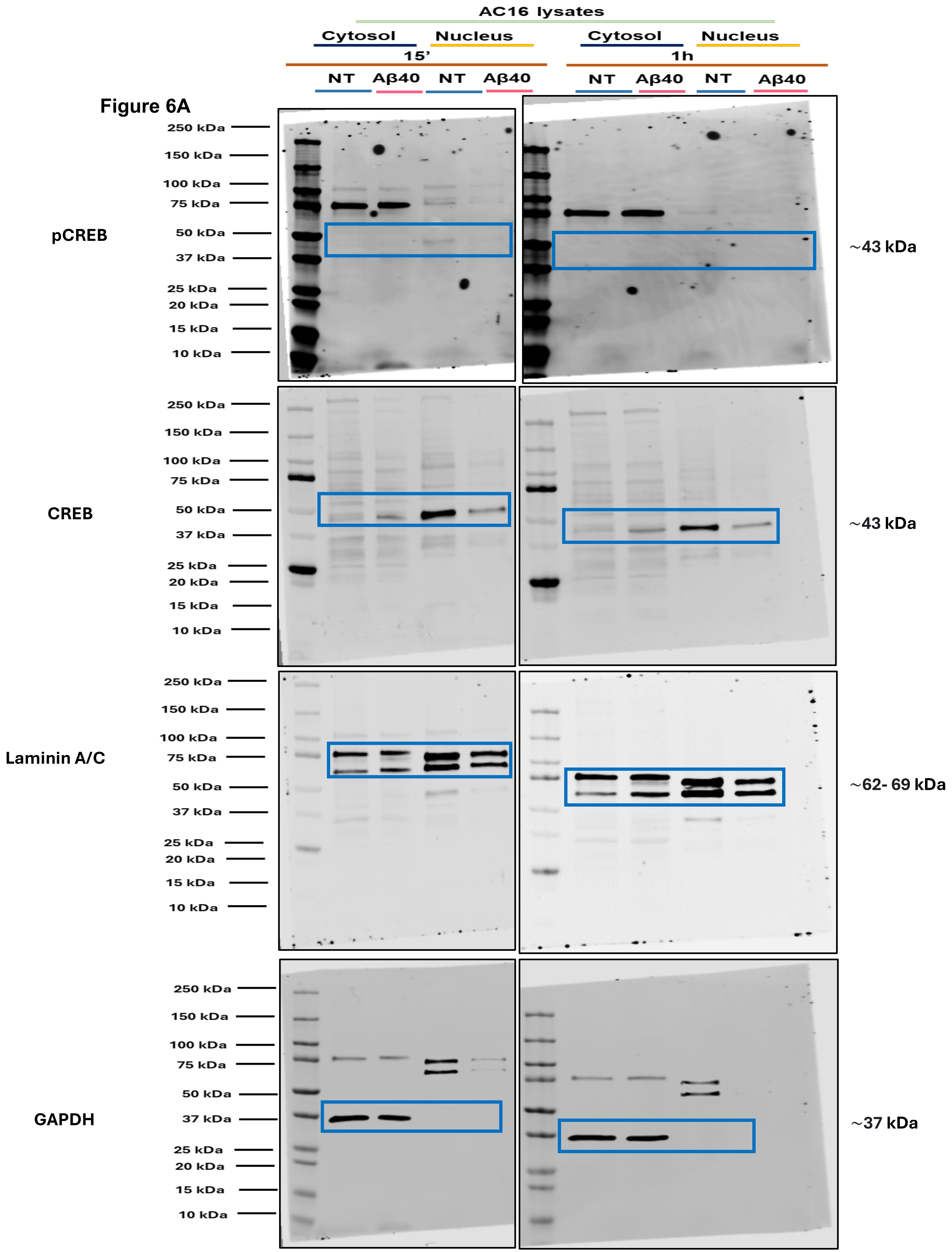

AC16 lysates
Cytosol
Nucleus
Cytosol
Nucleus
15'
1h
NT
Aβ40
NT
Aβ40
NT
Aβ40
NT
Aβ40
pCREB
CREB
Laminin A/C
GAPDH
250 kDa
150 kDa
100 kDa
75 kDa
50 kDa
37 kDa
25 kDa
20 kDa
15 kDa
10 kDa
~43 kDa
~43 kDa
~62- 69 kDa
~37 kDa

Figure 6C

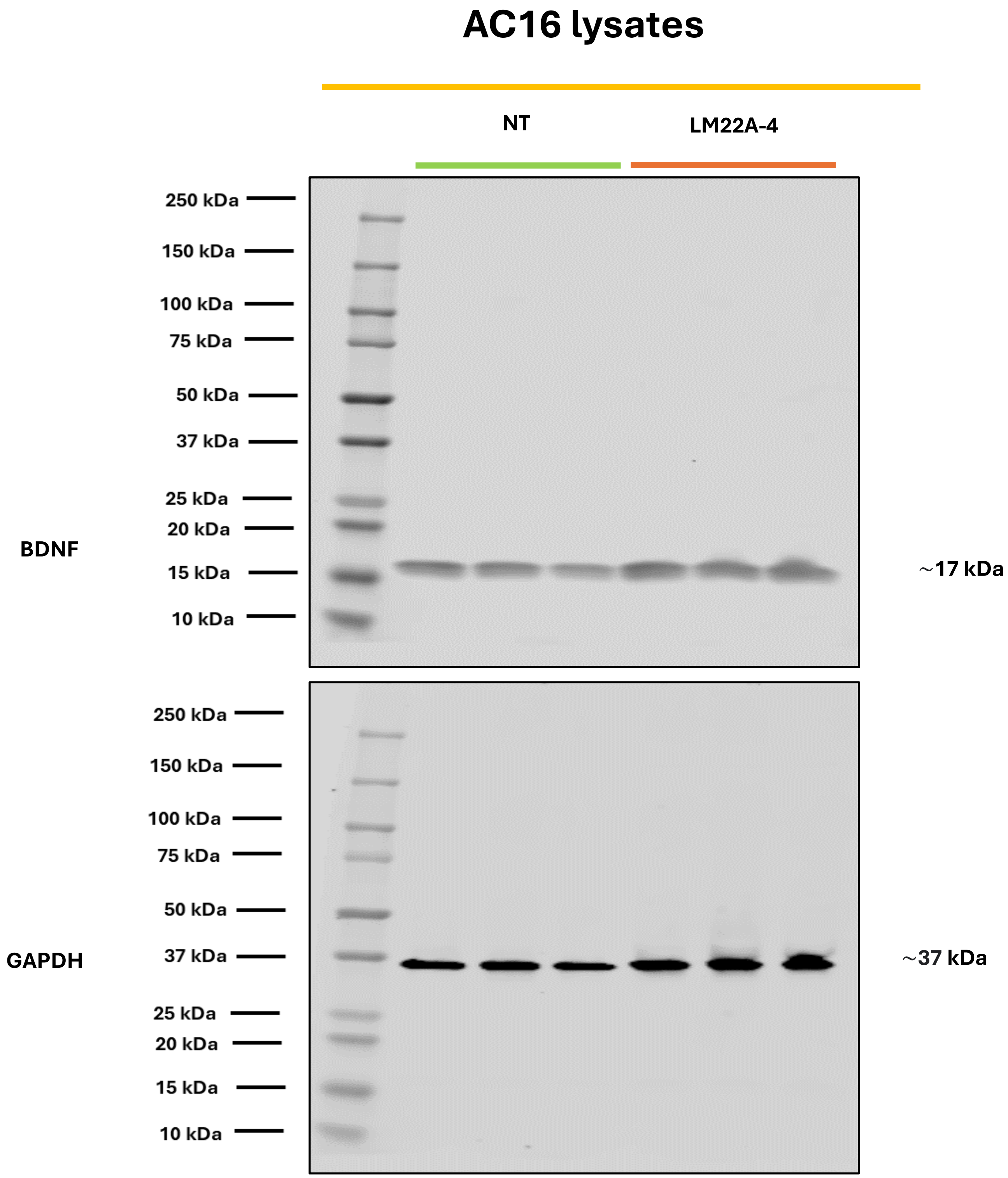

AC16 lysates
NT
LM22A-4
250 kDa
150 kDa
100 kDa
75 kDa
50 kDa
37 kDa
25 kDa
20 kDa
BDNF
15 kDa
10 kDa
~17 kDa
250 kDa
150 kDa
100 kDa
75 kDa
50 kDa
GAPDH
37 kDa
25 kDa
20 kDa
15 kDa
10 kDa
~37 kDa

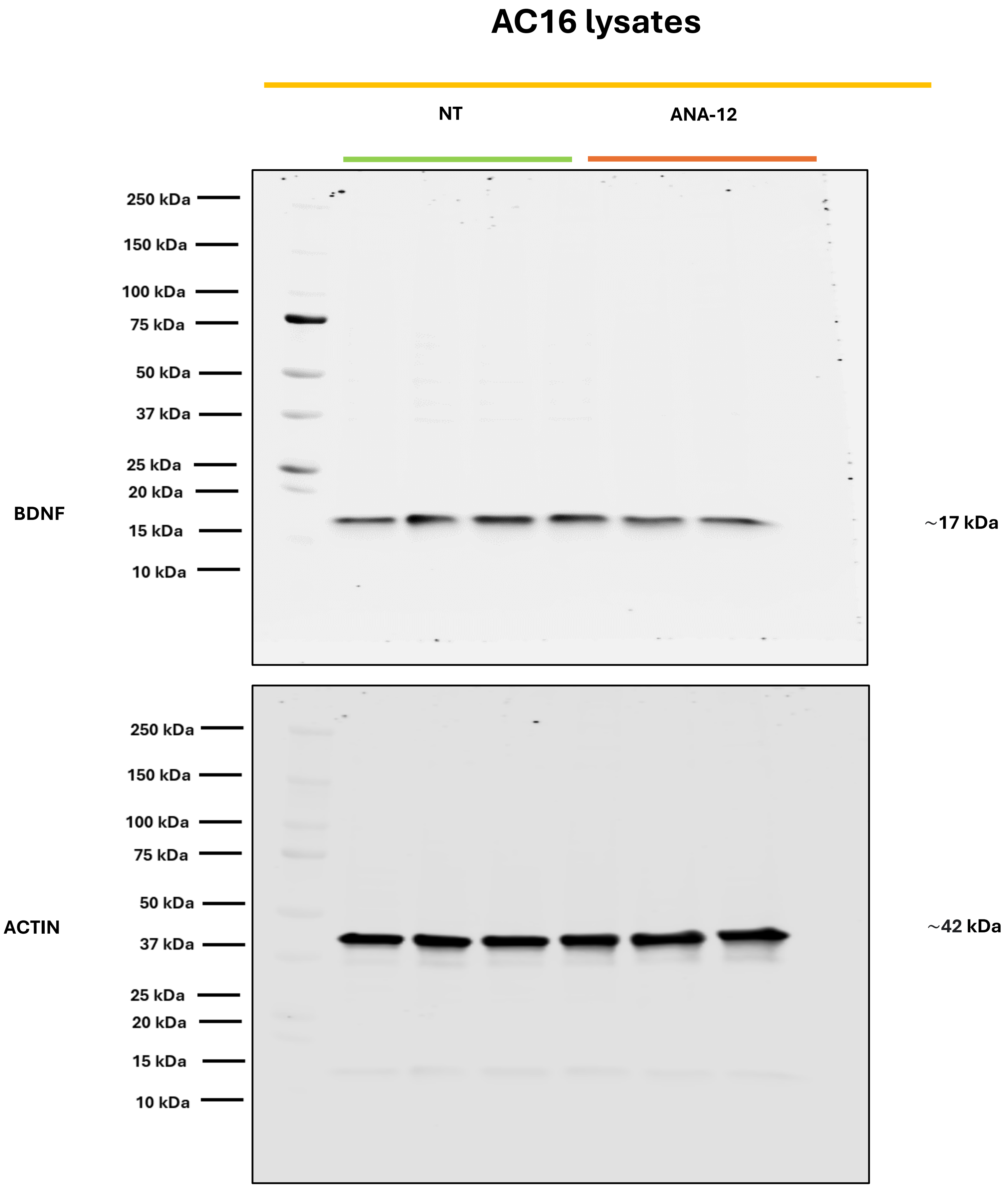
AC16 lysates
NT
ANA-12
BDNF
250 kDa
150 kDa
100 kDa
75 kDa
50 kDa
37 kDa
25 kDa
20 kDa
15 kDa
10 kDa
~17 kDa
ACTIN
250 kDa
150 kDa
100 kDa
75 kDa
50 kDa
37 kDa
25 kDa
20 kDa
15 kDa
10 kDa
~42 kDa

Figure 6F

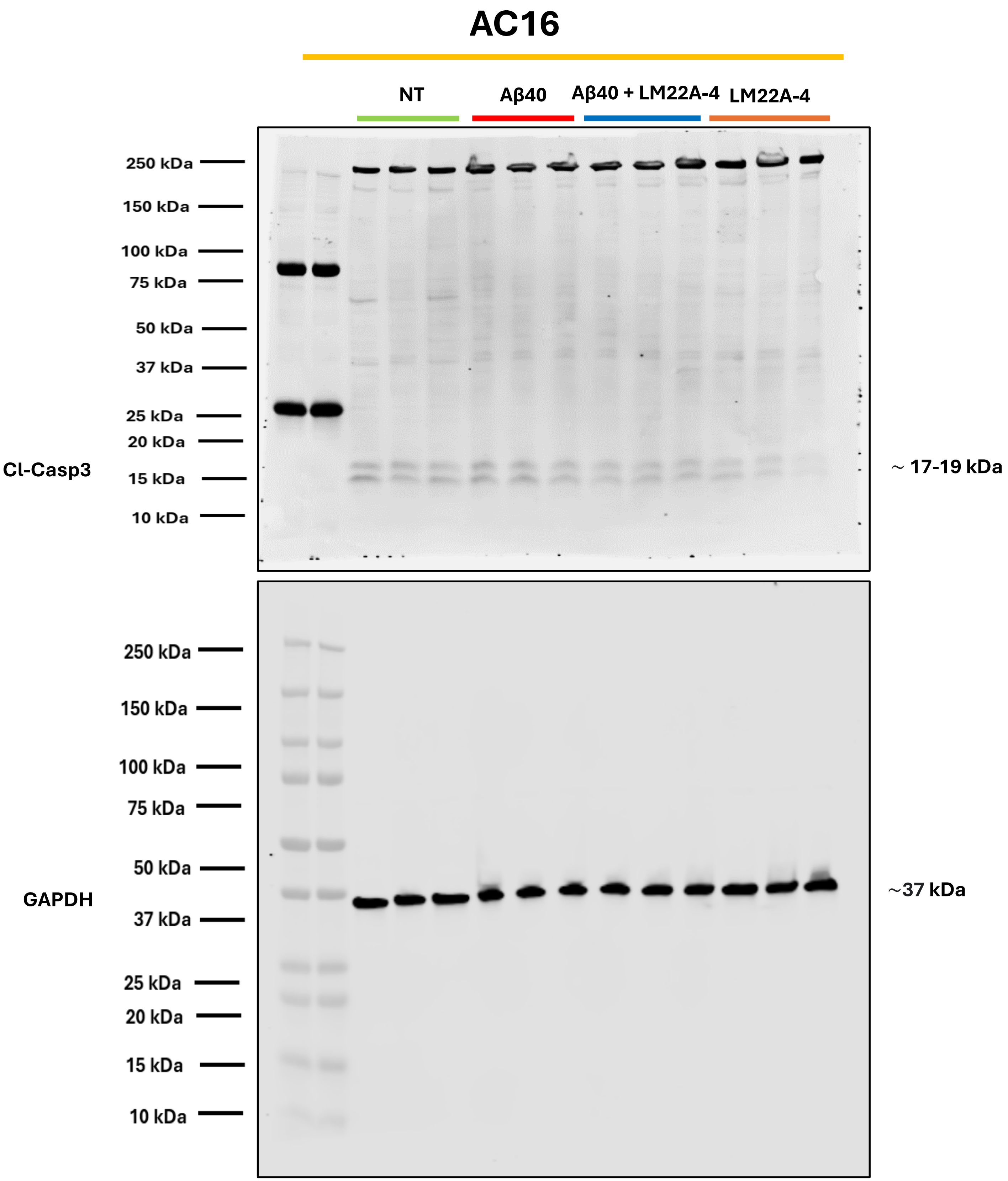
AC16
NT
Aβ40
Aβ40 + LM22A-4
LM22A-4
250 kDa
150 kDa
100 kDa
75 kDa
50 kDa
37 kDa
25 kDa
20 kDa
15 kDa
10 kDa
Cl-Casp3
~ 17-19 kDa
250 kDa
150 kDa
100 kDa
75 kDa
50 kDa
37 kDa
25 kDa
20 kDa
15 kDa
10 kDa
GAPDH
~37 kDa

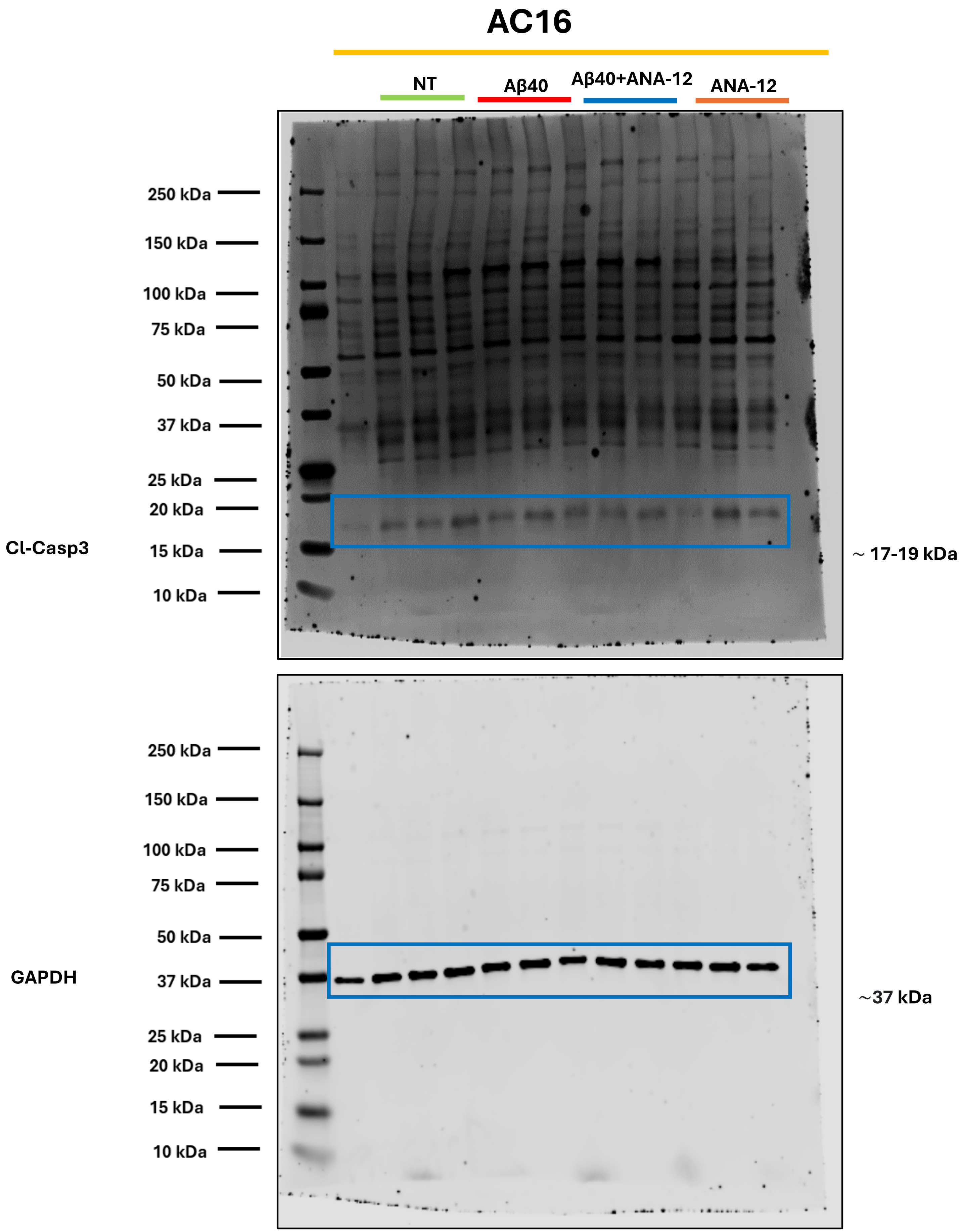
AC16
NT
Aβ40
Aβ40+ANA-12
ANA-12
250 kDa
150 kDa
100 kDa
75 kDa
50 kDa
37 kDa
25 kDa
20 kDa
15 kDa
10 kDa
Cl-Casp3
~ 17-19 kDa
250 kDa
150 kDa
100 kDa
75 kDa
50 kDa
37 kDa
25 kDa
20 kDa
15 kDa
10 kDa
GAPDH
~37 kDa

Supplemental Figure 1

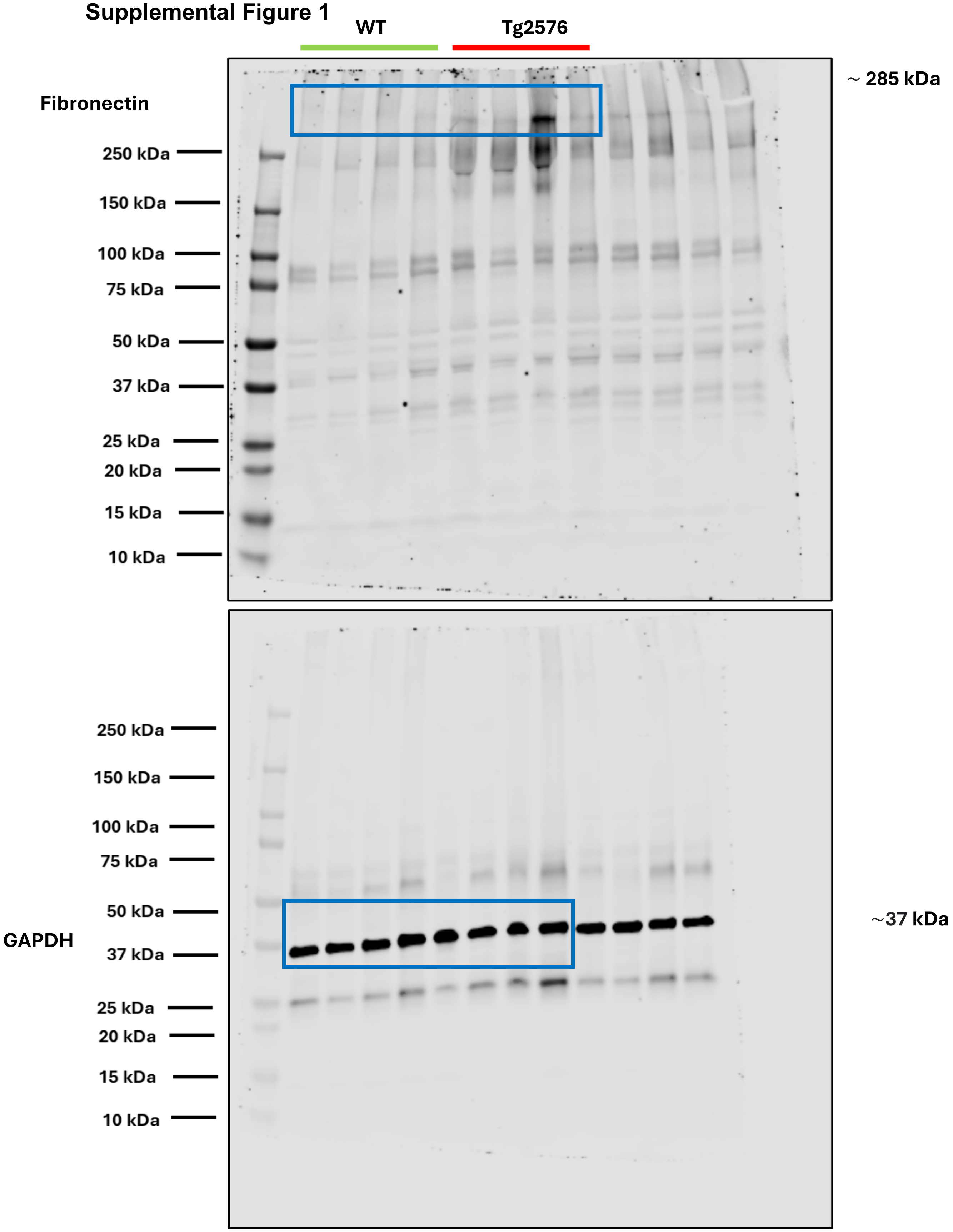

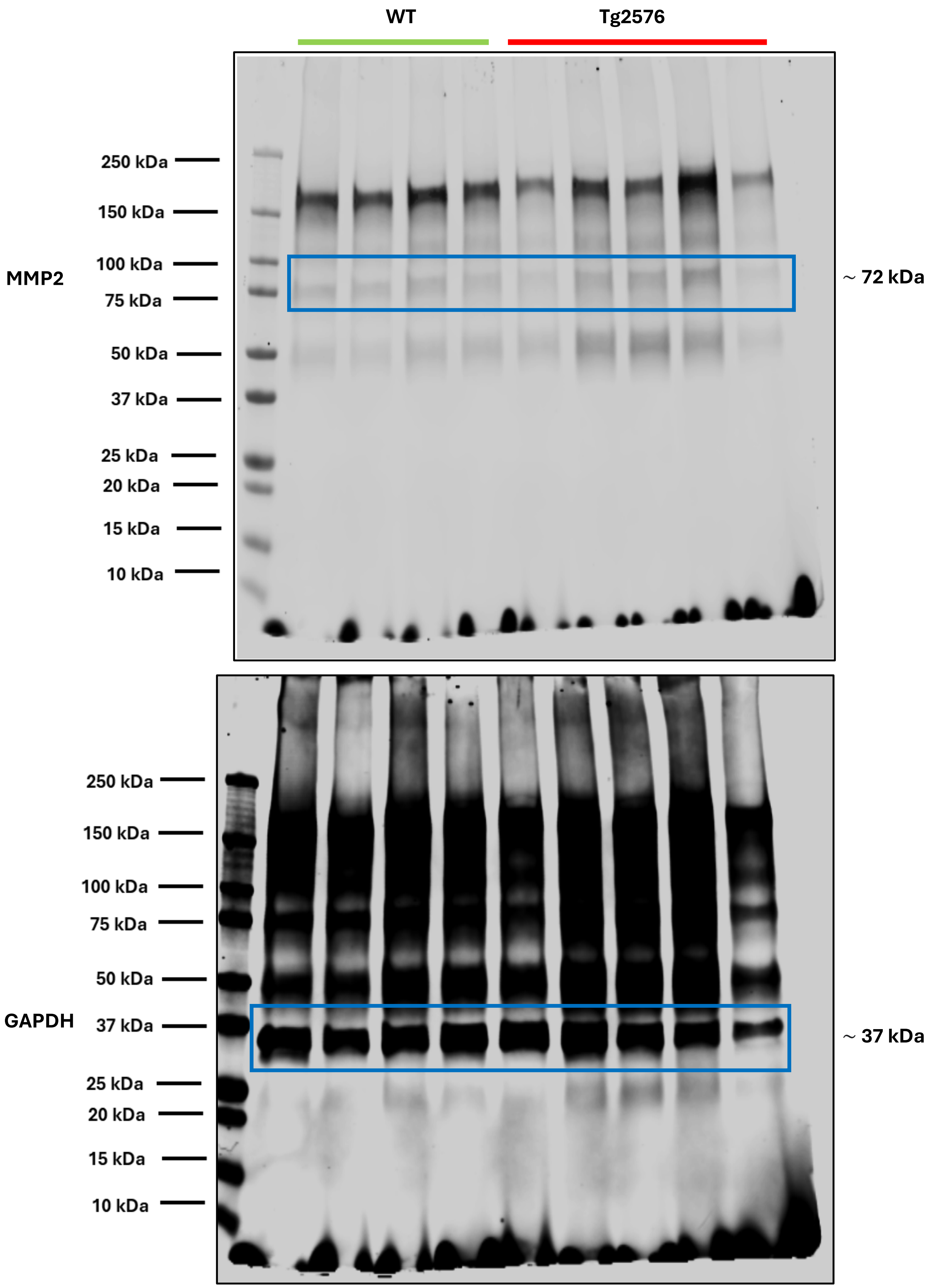
WT
Tg2576
MMP2
250 kDa
150 kDa
100 kDa
75 kDa
50 kDa
37 kDa
25 kDa
20 kDa
15 kDa
10 kDa
~ 72 kDa
GAPDH
250 kDa
150 kDa
100 kDa
75 kDa
50 kDa
37 kDa
25 kDa
20 kDa
15 kDa
10 kDa
~ 37 kDa

Supplemental Figure 6A

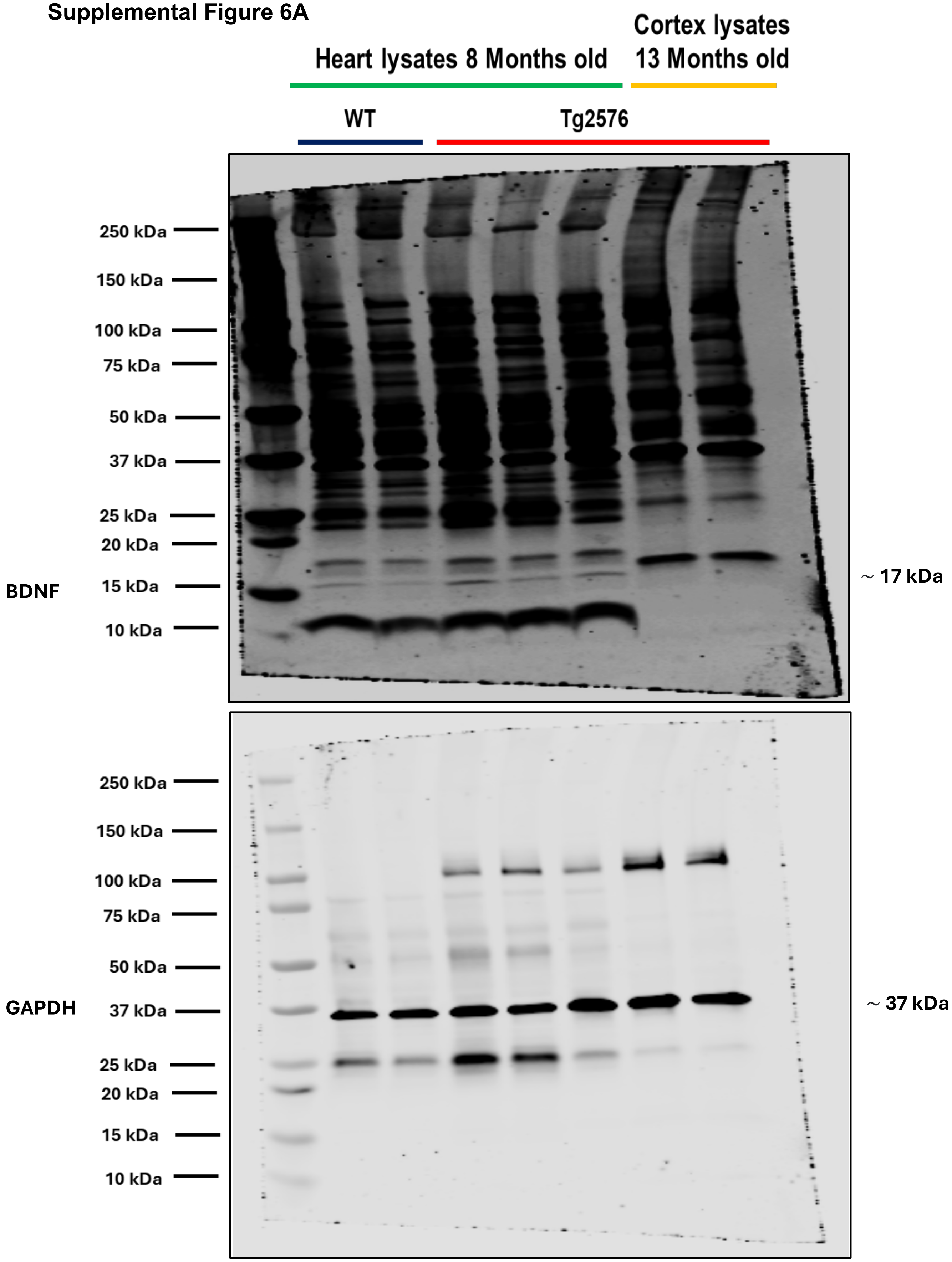

Supplemental Figure 6B

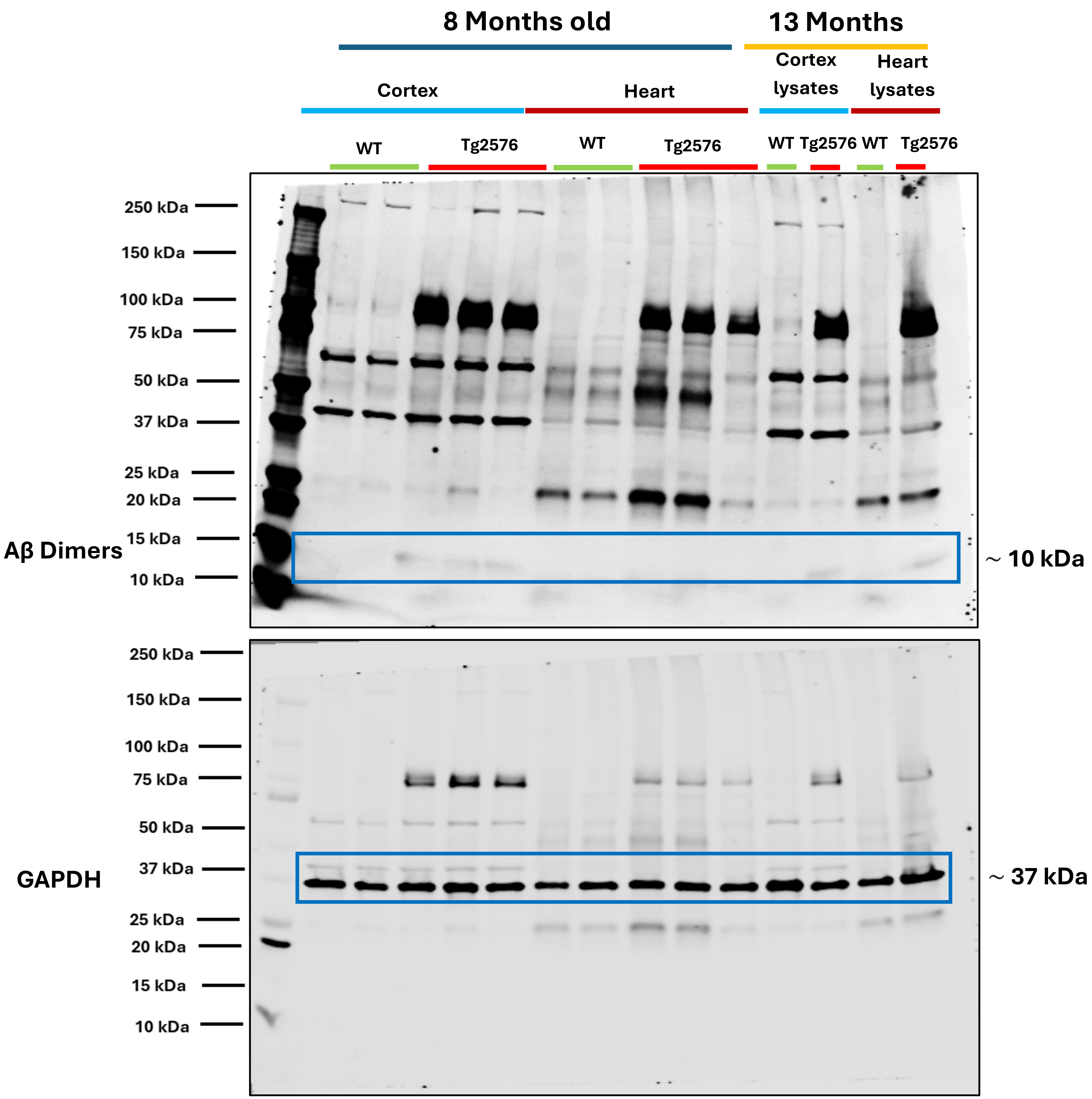

Supplemental Figure 9A

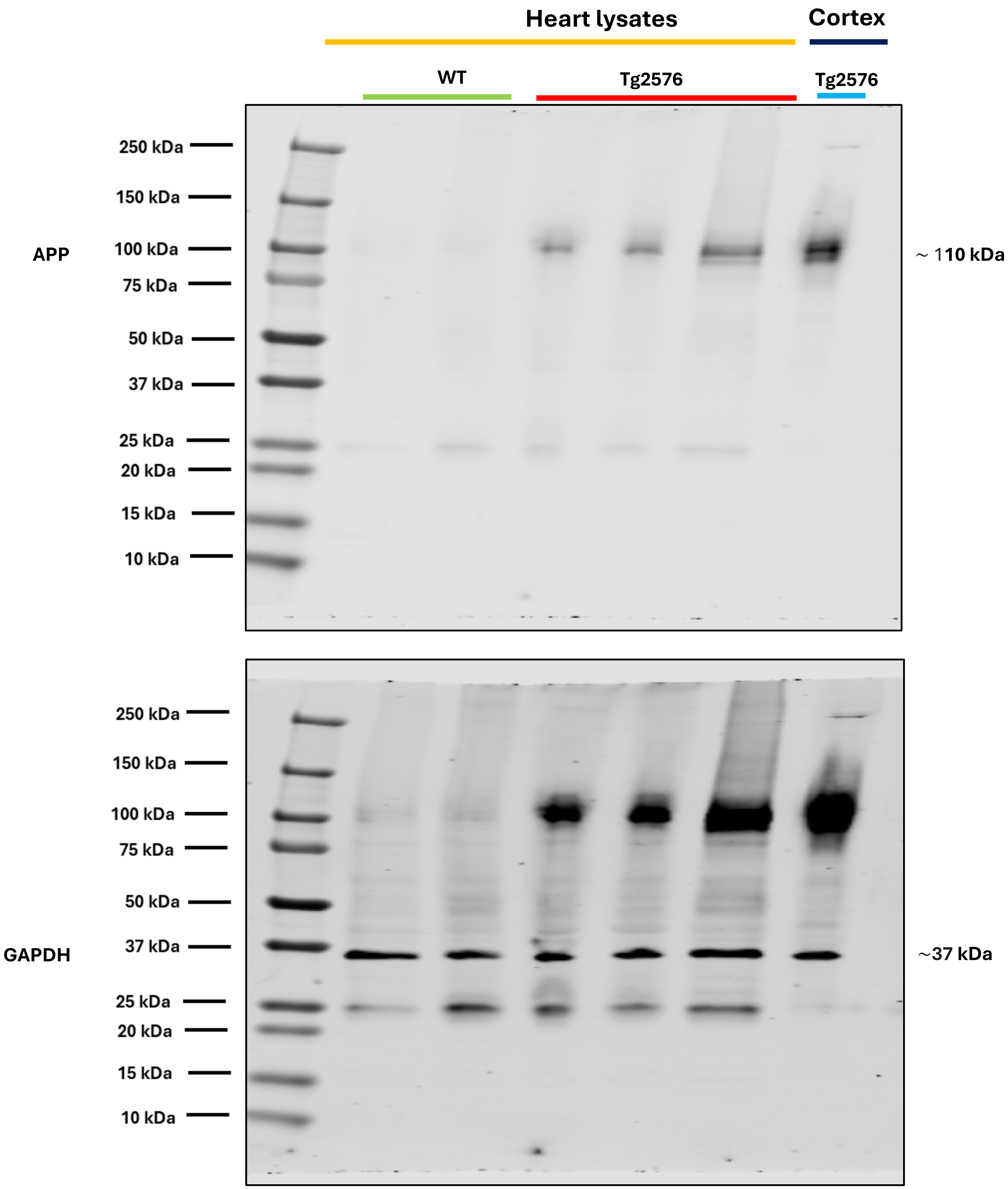

Supplemental Figure 9B

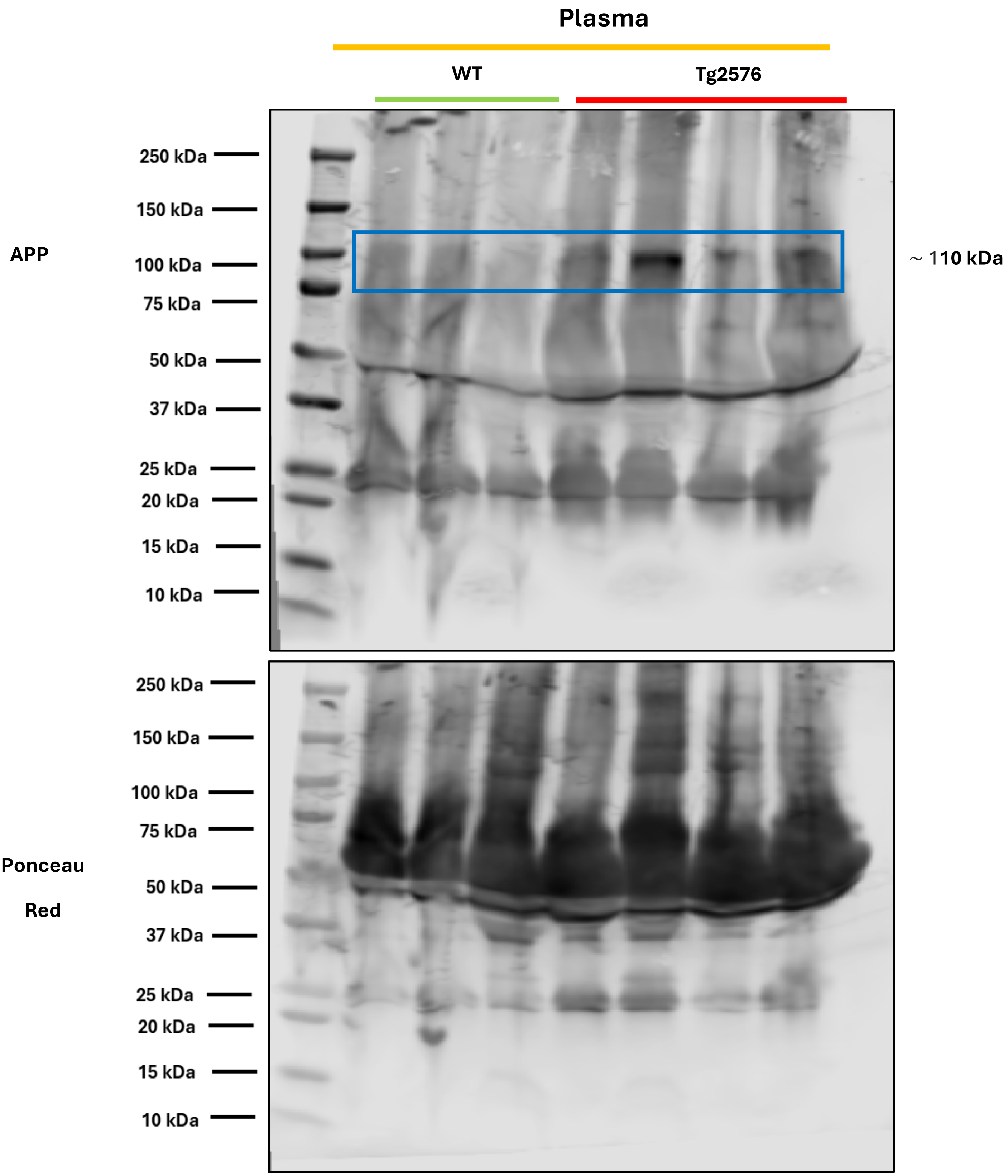

Supplemental Figure 11A

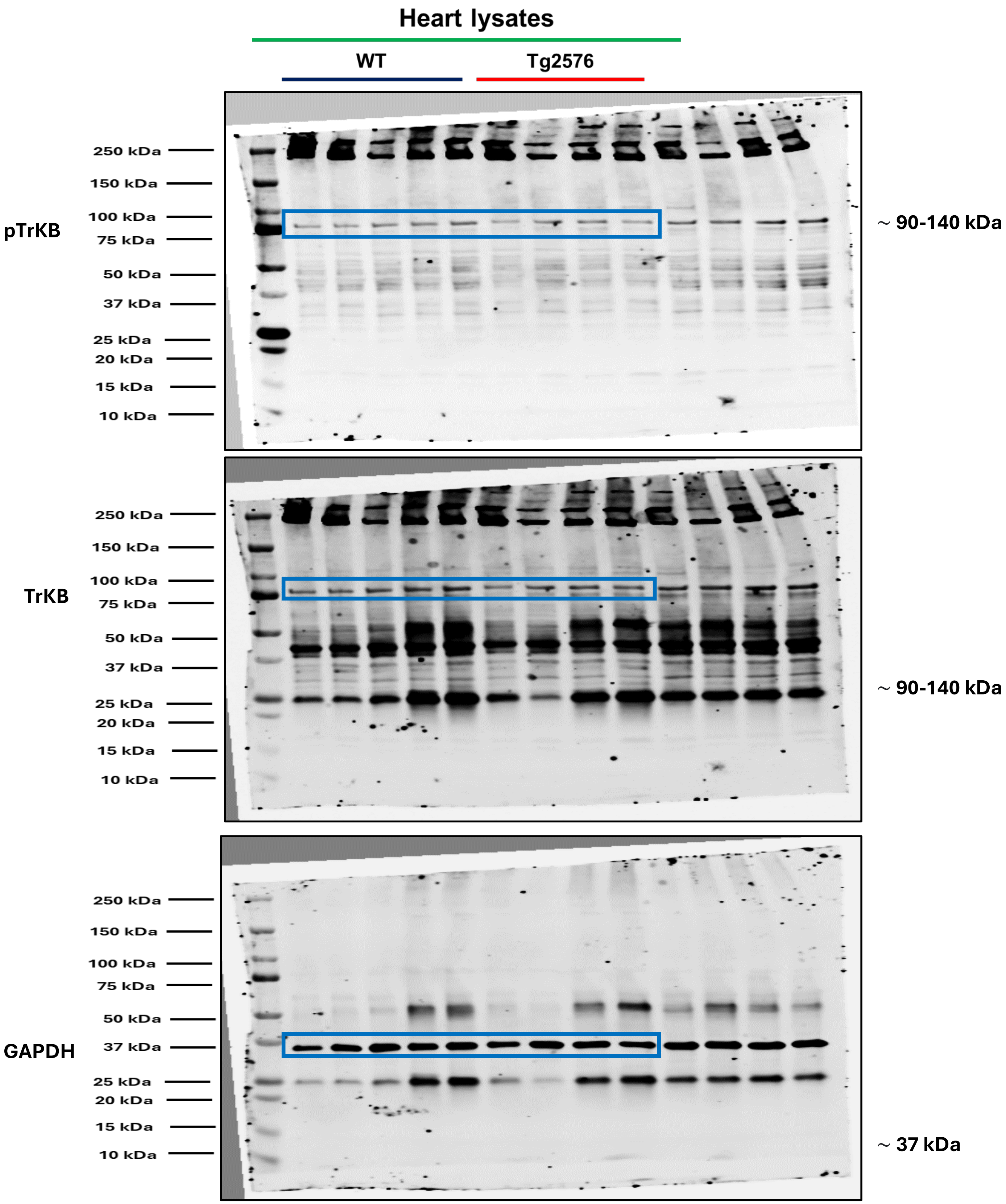

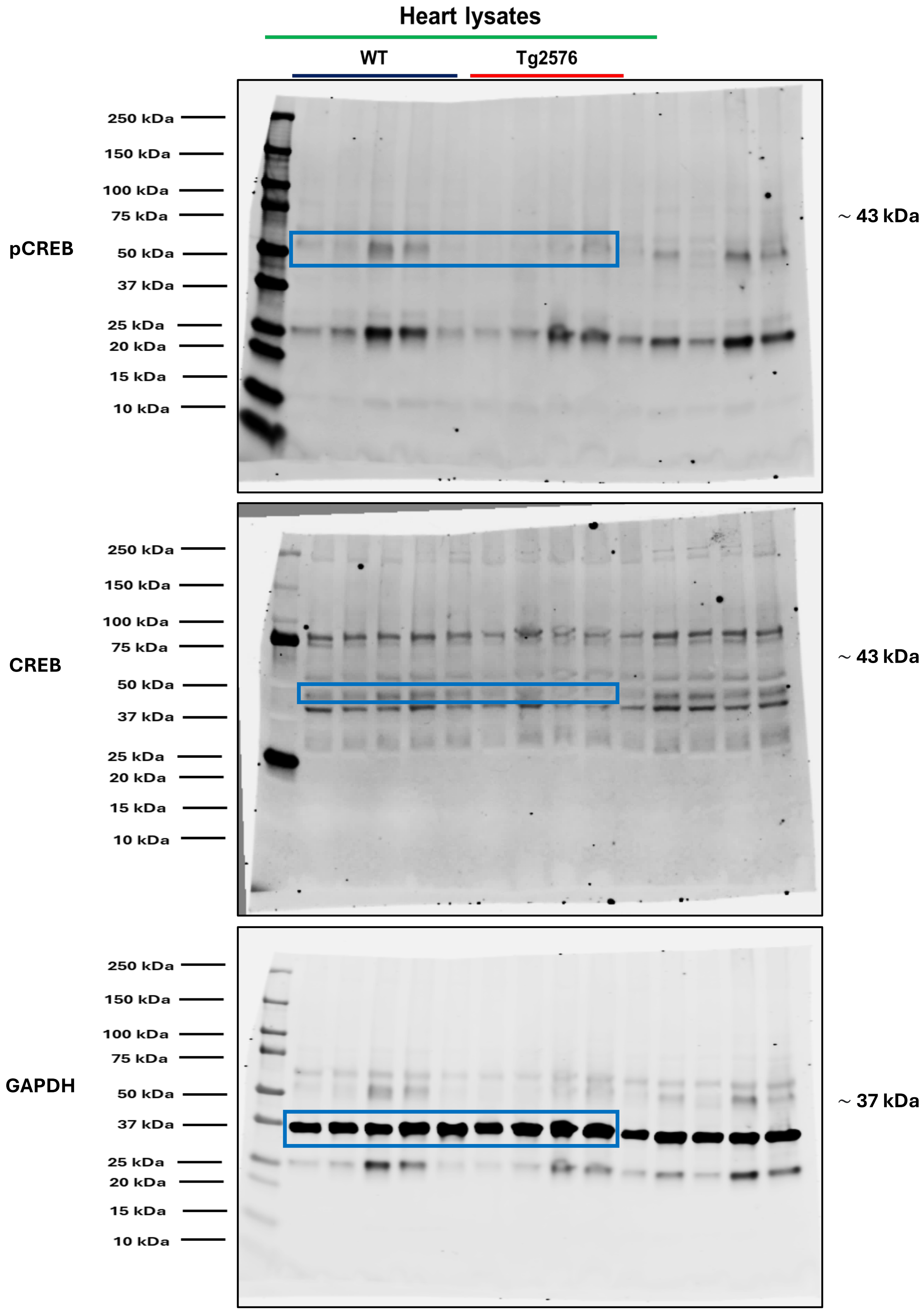

Heart lysates
WT
Tg2576
pCREB
CREB
GAPDH
250 kDa
150 kDa
100 kDa
75 kDa
50 kDa
37 kDa
25 kDa
20 kDa
15 kDa
10 kDa
~ 43 kDa
~ 43 kDa
~ 37 kDa

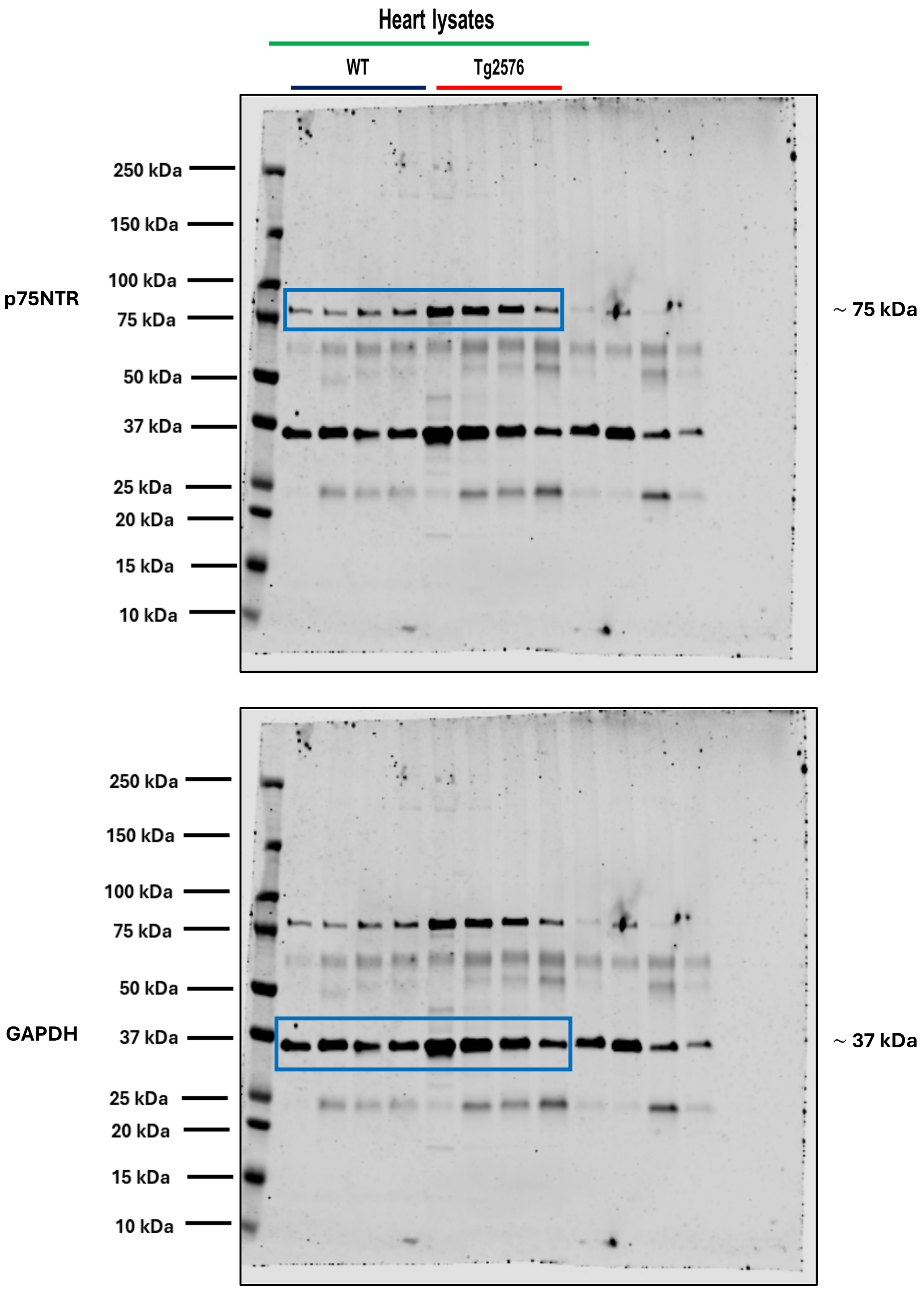
Heart lysates
WT
Tg2576
p75NTR
250 kDa
150 kDa
100 kDa
75 kDa
50 kDa
37 kDa
25 kDa
20 kDa
15 kDa
10 kDa
~ 75 kDa
GAPDH
250 kDa
150 kDa
100 kDa
75 kDa
50 kDa
37 kDa
25 kDa
20 kDa
15 kDa
10 kDa
~ 37 kDa
